# Splice_sim: a nucleotide conversion-enabled RNA-seq simulation and evaluation framework

**DOI:** 10.1186/s13059-024-03313-8

**Published:** 2024-06-25

**Authors:** Niko Popitsch, Tobias Neumann, Arndt von Haeseler, Stefan L. Ameres

**Affiliations:** 1 Max Perutz Labs, Vienna Biocenter Campus (VBC), Vienna, A-1030 Austria; 2 Max Perutz Labs, Department of Biochemistry and Cell Biology, University of Vienna, Vienna, A-1030 Austria; 3 Quantro Therapeutics, Vienna, A-1030 Austria; 4 Vienna Biocenter PhD Program, a Doctoral School of the University of Vienna and Medical University of Vienna, Vienna, A-1030 Austria; 5 Center for Integrative Bioinformatics Vienna, Max Perutz Labs, University of Vienna, Medical University of Vienna, Vienna, A-1030 Austria; 6 Bioinformatics and Computational Biology, Faculty of Computer Science, University of Vienna, Vienna, A-1090 Austria; 7 Institute of Molecular Biotechnology, IMBA, Vienna Biocenter Campus (VBC), Vienna, A-1030 Austria

**Keywords:** Nucleotide conversion sequencing, Metabolic RNA labeling, SLAMseq, RNA-BS-seq, 3′ end sequencing, Spliced read mapping, Read mapping accuracy

## Abstract

Nucleotide conversion RNA sequencing techniques interrogate chemical RNA modifications in cellular transcripts, resulting in mismatch-containing reads. Biases in mapping the resulting reads to reference genomes remain poorly understood. We present splice_sim, a splice-aware RNA-seq simulation and evaluation pipeline that introduces user-defined nucleotide conversions at set frequencies, creates mixture models of converted and unconverted reads, and calculates mapping accuracies per genomic annotation. By simulating nucleotide conversion RNA-seq datasets under realistic experimental conditions, including metabolic RNA labeling and RNA bisulfite sequencing, we measure mapping accuracies of state-of-the-art spliced-read mappers for mouse and human transcripts and derive strategies to prevent biases in the data interpretation.

**Supplementary Information:**

The online version contains supplementary material available at 10.1186/s13059-024-03313-8.

## Background

Nucleotide conversion (NC) RNA sequencing techniques are powerful methods to study post-transcriptional modifications across a wide range of organisms and cell types [1]. In these techniques, RNA is exposed to dedicated nucleotide conversion chemistry and subjected to cDNA library preparation and high-throughput sequencing, ultimately resulting in reads that exhibit zero, one, or more specific NCs. Reads are then mapped to a reference sequence and grouped into labeled (one or more NC) and unlabeled (no NC) reads. Grouped read counts are finally combined to quantitative measures of interest and analyzed/interpreted to gain novel biological insights.

Several NC RNA-seq protocols that monitor a range of RNA modifications but differ in the type and penetrance of NCs have been introduced recently. These include metabolic RNA labeling techniques with low (1–5%) NC rates that are being used to study the cellular rates of RNA synthesis, processing, translation, and decay. Here, cells are subjected to metabolic RNA labeling with the nucleotide analog 4-thiouridine (4sU). Upon RNA extraction and chemical treatment, 4sU is converted into cytosine or cytosine analogs, allowing to distinguish newly synthesized from pre-existing transcripts due to the presence of T-to-C conversions. The fraction of converted reads (FCR; Table 1) per transcript annotation is, for example, used to estimate half-lives of RNA molecules [2–4]. In contrast to metabolic RNA sequencing, RNA bisulfite sequencing (RNA-BS-seq) is an example for a NC RNA-seq protocol with very high (> 98%) conversion rates. This approach enables the mapping of posttranscriptional cytosine methylation that has been proposed to play a role in RNA regulation, structure, stability, translation and, if mis-regulated, also in disease (progression) [5–8]. Here, methylated cytosines are protected from being deaminated into uracil upon bisulfite treatment and methylation rates of 5-methylcytosine (m^5^C) sites are assessed upon sequencing of cDNA libraries by determining the fraction of unconverted reads at any given cytosine site (metR, methylation rate; Table 1).

**Table 1:** Exemplary measures based on the comparison (ratio) of different (NC) read groups

Measure	Simplified formula	Applications
**FCR**: fraction of converted reads	$\frac{\#\text{converted}\_\text{reads}}{\#\text{all}\_\text{reads}}$	e.g., RNA stability measurement (half-life estimation)
**metR:** methylation rate	$\frac{\#\text{unconverted}\_\text{reads}\_\text{at}\_\text{site}}{\#\text{all}\_\text{reads}\_\text{at}\_\text{site}}$	e.g., post-transcriptional RNA methylation
**FMAT:** fraction of mature isoform	$\frac{\#\text{mature}\_\text{isoform}\_\text{reads}}{\#\text{all}\_\text{reads}}$	e.g., RNA splicing kinetics; applied to converted and unconverted reads

Depending on conversion-frequencies, NC RNA-seq datasets are expected to be vulnerable to biases in mapping due to the presence of mismatches that may affect their unique assignment to specific regions in the genome: If, for example, converted reads from a metabolic labeling experiment show considerably lower mapping accuracies than unconverted reads due to increased mismatches to the reference sequence, then resulting FCR values and in consequence derived half-lives would be affected. Accordingly, variations in mapping accuracies for reads with different numbers of m^5^C sites may consequently lead to false-negative and false-positive annotation of m^5^C sites. Thus, to estimate the reliability of these measures, we need to understand how NCs influence the accuracy of mapping reads to their originating genomic location.

Here, we set out to study mapping accuracies of NC reads, focusing on the evaluation of splice-aware read mappers because NC conversion approaches are often applied to problems that benefit from or require spliced read alignments. Examples include the (relative) quantification of different gene isoforms to investigate alternative splicing mechanisms or intron splicing kinetics [9] (by comparing read counts from (NC converted) unspliced (premature) isoforms with counts from fully spliced (mature) isoforms, FMAT; Table 1). RNA-seq quantification based on spliced alignments was furthermore reported to be more accurate when compared to transcriptome mapping and lightweight quasi-mapping approaches [1, 10]. We do, however, also evaluate the impact of NC on transcript quantification by 3′ end mRNA sequencing, an alternative, cost-efficient protocol that does not require spliced read mapping [11].

Generally, read mapping accuracy is influenced by read length and the number of mismatches to the reference sequence (by NCs and sequencing errors) as well as the general genome mappability of the respective genomic sequence. Genome mappability describes the ability of read mappers to accurately place and align reads of a specific length to respective genomic regions. It is largely determined by the repetitiveness of the genome [12]. Highly repetitive regions account for large shares of eukaryotic genomes and are found in non-coding as well as coding regions (Additional file 1: Figure S1 [13, 14]). Their reduced mappability results in misplaced (false-positive, FP) and missing (false-negative, FN) reads and consequently reduced reliability of biological interpretation derived from respective read alignments.

While mappability of unmodified short reads has been intensely studied in the past [12, 14, 15], much less has been done to address mappability of NC reads. A notable exception is bisulfite sequencing where the bisulfite reaction converts unmethylated cytosine to uracil that is ultimately read as thymine. The high conversion efficiency of this reaction leads to reads with high fractions of C-to-T conversions that are most effectively mapped using specially designed alignment tools, e.g., Bismark [16], BSMAP [17] or meRanGs [18]. Most current approaches essentially employ a (partial) three-nucleotide letter (3N) alignment strategy: all cytosines in the reads and the reference sequence are converted to thymines and read mapping is based on the remaining three bases (T, A, G). While this makes 3N mappers insensitive to the number of converted cytosines in the reads, it reduces mappability due to the lower complexity of the mapped sequences and their targets. First approaches to investigate this systematically can be found in Karimzadeh et al. [19], who identified uniquely mappable regions in unconverted and fully bisulfite-converted human and mouse genomes. In [20], the authors present an extension to the mapper Segemehl that employs a hybrid approach, combining a seed-based search on a reduced 3N alphabet with a bisulfite-sensitive semi-global alignment that improved overall mapping sensitivity. More recently, Zhang et al. [21] evaluated their HISAT-3N mapper that implements a generalized 3N alignment strategy, allowing arbitrary NCs, on simulated bisulfite and metabolic labeling data. Besides reporting improved mapping accuracies when comparing to other 3N mappers, they also observed an expected increase in multi-mapped reads but overall similar mapping accuracies when comparing 4N and 3N alignments of unmodified reads.

Here, we extend and generalize these findings by comprehensively assessing mapping accuracies of NC reads under different conditions (low to high conversion rates) and their impact on downstream analyses. For this purpose, we developed *splice_sim*, a specialized RNA-seq simulation and evaluation pipeline that (i) simulates short reads with realistic sequencing errors from arbitrary mixes of (partially) spliced and unspliced isoforms per transcript, (ii) introduces arbitrary NCs with a given rate as well as a configurable set of single-nucleotide variations (SNVs) into these reads, and (iii) creates mixed models of converted and unconverted reads. *Splice_sim* then maps simulated reads using a configurable set of mappers and calculates differential alignments between simulated “truth” and mapper output, enabling a comprehensive evaluation of mapping accuracies under different nucleotide conversion rates.

Using *splice_sim*, we generated deep (100X coverage) simulated metabolic labeling RNA-seq datasets with altering conversion rates (1–10%) for mouse and human transcriptomes and evaluated mapping accuracies of converted and unconverted reads for HISAT-3N [21] and STAR [22], a popular spliced read mapper that does not implement a 3N mapping strategy, for various genomic regions of interest (exons, introns, splice junctions, and whole transcripts). We then evaluated the effects of NC mapping accuracies on decay half-life and isoform mix reconstruction and applied several strategies to correct/improve those measures using our mapping accuracy scores. As a result, we provide comprehensive transcriptome-wide NC mapping accuracy tables for more than 50k mouse and human transcripts each. We repeated our analysis with simulated RNA-BS-seq data (evaluating HISAT-3N, Segemehl and meRanGs, a specialized bisulfite read mapper based on STAR) and evaluated downstream effects on methylation site calling. Finally, we used *splice-sim* to evaluate different analysis strategies for targeted RNA sequencing, such as mRNA 3′ end sequencing, an alternative cost-effective approach for quantifying (NC) mRNA abundances.

Our study demonstrates the negative impact of nucleotide conversion rates on the accuracy to estimate measures with direct biological interpretation and thereby sheds light on the dimension of this problem for real-world experiments. We provide mapping accuracy tables for meaningful biological units of interest (transcripts, exons, introns, and splice junctions) and showcase simple algorithms for improving accuracies in problematic regions or for filtering error prone data sections. Using s*plice_sim*, we identified such regions in numerous members of hallmark gene sets [23], demonstrating their biological relevance (Additional file 1: Figure S1). Finally, we provide users with a simulation and evaluation pipeline that can be used to evaluate existing analysis pipelines/tools or to conduct sophisticated in silico experiments that can be performed for any species and transcript annotation of interest.

## Results

### Splice_sim systematically evaluates the performance of read mappers on NC RNA-seq data

**Fig. 1:**
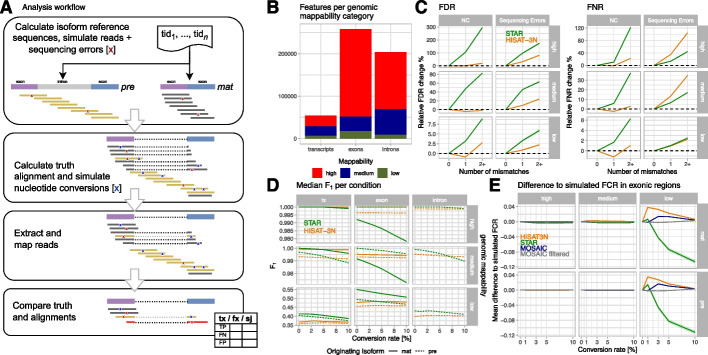
Analysis workflow and NC mapping accuracies for simulated mouse metabolic labeling data. **A** Analysis workflow overview: briefly, we simulated short reads with realistic sequencing error (red X) for premature and mature isoforms, calculated truth alignments, and injected nucleotide conversions with configured conversion rates. Simulated reads were mapped by the evaluated read mappers and resulting alignments were compared to the simulated data. Finally, grouped count tables with true positive (TP), false positive (FP), and false negative (FN) counts per annotation of interest (tx: transcripts, fx: exons + introns, sj: splice junctions) were created and analyzed. **B** Numbers of analyzed **m_big** annotations with high (> 0.9), medium, and low (< 0.2) mean genome mappability. **C** Changes of false discovery (FDR = FP/(TP + FP) and false negative (FNR = FN/(TP + FN)) rates by number of mismatches per read compared to reads without mismatches, stratified by mappability and type of mismatch (either simulated NC or random sequencing errors). The plots show median FDR/FNR and interquartile regions (shaded areas) across three **m_big** replicates for STAR (green) and HISAT-3N (orange) alignments. This analysis included ~ 12B reads originating from premature isoforms and their classification (TP, FP, FN) with respect to whole-transcript annotations. **D** Median F_1_ measure per mapper and originating isoform (pre: premature, mat: mature) for different genomic annotations (tx: whole transcript), stratified by mappability. **E** Mean difference to simulated, exonic FCR (fraction of converted reads) per mapper and for a “mosaic” approach where the mapper with the smallest difference to the simulated value was chosen. The mosaic approach reduces differences to simulated values and when removing exons where none of the two mappers showed good results, reconstruction is nearly perfect (“mosaic filtered,” see main text). Note that a corresponding plot for human data is provided in Additional file 1: Figure S6 for comparison

Using *splice_sim*, we simulated a deep metabolic labeling single-read 100 nt dataset (m_big) covering > 50k GENCODE (https://www.gencodegenes.org/) annotated canonical mouse (mm10) transcripts. For each transcript, we simulated the premature (unspliced) and mature (fully spliced) isoform with a target coverage of ~ 50X per isoform, i.e., ~ 100X overall. We simulated three replicates with five different T/C conversion rates (0,1,3,5,10%, typically observed in metabolic RNA-seq time course experiments) each and mapped the simulated data with HISAT-3N and STAR. *Splice_sim* then assessed true-positive (TP), false-negative (FN), and false-positive (FP) read counts for different annotation sets: whole transcripts, exons, introns, and splice junctions. For the latter, we counted donor overlapping, acceptor overlapping, and spliced reads separately. Details about the counting algorithm are provided in Additional file 1: Supplement; a graphical overview of the analysis workflow is shown in Fig. 1A. Mapping accuracy per annotation was quantified using the F_1_ measure (an accuracy measure that incorporates precision and recall into one single score) for mature and premature isoforms separately. As genome mappability has arguably a major impact on NC mapping accuracy and is widely used to filter data, we grouped genomic annotations into three mappability classes (high, medium, low; cf. the “Methods” section and Fig. 1B) based on the observed mappability distributions.

First, we analyzed over 12 billion mapped reads to quantify the impact of NC on mapping accuracy using STAR and HISAT-3N. As expected, NC and sequencing errors increased false discovery (FDR) and false negative rates (FNR) for both mappers, as read alignment becomes more difficult with increased numbers of mismatches to the originating genomic sequence (Fig. 1C). Simulated T/C nucleotide changes, however, did not strongly affect HISAT-3N data as those were essentially masked out due to the applied 3N mapping approach. We found residual elevated FP rates with increasing conversion rates in HISAT-3N to be caused by reads (~ 2%) mapping to the wrong strand (cf. Additional file 1: Figure S2) and corresponding elevated FN rates to be caused by repetitive regions of the same base composition as the introduced base conversions, likely confusing the repeat index during mapping (Additional file 1: Figure S3). Note that absolute FDR/FNR is dominated by genome mappability which is why the relative increase due to additional mismatches is much smaller for low mappability regions. We then assessed mapping accuracies for different genomic features (Fig. 1D). As expected, mapping accuracy decreases with genomic mappability across all categories. Overall, both evaluated mappers showed high accuracies except for features with low mappability where STAR slightly outperformed HISAT-3N. In accordance with our initial analysis, we observed that STAR’s performance dropped with increasing conversion rates due to increasing mismatches between reads and reference sequence, while HISAT-3N was largely unaffected due to its 3N mapping approach. For both mappers, we observed higher mapping precision than recall (Additional file 1: Figure S4) which indicates that FNs are the main factor for reduced accuracies. We then investigated the effect of reduced mapping accuracies on the fraction of converted reads (FCR), an exemplary measure used in downstream analyses to estimate transcript stabilities (Table 1). We compared exonic FCR values (a plot showing intronic and whole-transcript data is in Additional file 1: Figure S5) derived from mapper specific alignments to the true (simulated) FCR which revealed that both mappers indeed have problems reconstructing this measure in the low mappability segment (Fig. 1E). STAR underestimates the real value while the opposite is true for HISAT-3N, although the latter showed less deviations from the true values. The difference to simulated values is dependent on conversion rates, particularly for STAR. We then implemented a “mosaic” analysis strategy to enhance FCR reconstruction accuracy. In this approach, we selected the FCR values for each interval (e.g., exon) from the mapper that most closely matched the simulated values. This strategy is termed “mosaic” because it involves choosing the most effective mapper for each specific interval, akin to assembling a mosaic where each piece is optimally chosen based on performance. When combining this “mosaic” approach with a filtering strategy that removed transcripts for which none of the mappers returned results close to the simulation (see the “Methods” section), the overall mean FCR approached the simulated (true) value and omitted only ~ 1.3k (~ 8%) of low mappability exons. We concluded that combining STAR and HISAT-3N in a genomic-location-specific manner can enhance the quantitative analysis of NC datasets particularly for *loci* that suffer from low overall mappability.

### Splice_sim instructs mapping approaches in a reduced sequence space

Emerging RNA sequencing approaches that target only selected transcript features are gaining popularity by their ability to multiplex in a cost-effective manner large sample numbers within one library. 3′ end mRNA sequencing [1, 11, 24], for instance, targets not the entire transcript sequence but only its 3′ end (typically the last 200 bp) and considers the resulting counts representative for the whole transcript. In addition, the use of oligo(dT) primers for reverse transcription that binds poly-A tails potentially also enriches for any A-rich region in the transcript body resulting in reads that stem from such “internal priming” events and “pollute” the overall signal thereby reducing achieved mapping accuracies. To showcase how our tool can be used to select an optimal read mapping strategy in such a scenario, we configured *splice_sim* to evaluate 3′ end mapping accuracies and their impact on downstream analyses in a side-by-side comparison with the full transcript sequencing approach. To cover also internal priming events inherent to 3′ end sequencing, we considered two possible extremes: (1) clean amplification of the 200 bp 3′ ends only and (2) simulating reads from the entire transcript in case there is internal priming along the entire transcript (“transcript noise”). In addition, we investigated distinct mapping strategies by mapping to (i) the whole genome, (ii) the transcript sequences, and (iii) their 3′ end sequences. Finally, in all cases, only reads overlapping 3′ end intervals were counted (Additional file 1: Figure S7). When comparing mean genome mappability for 3′ end and whole-transcript annotations, we found the former to be generally higher, irrespective if calculated on the genome level or transcriptome level (Additional file 1: Figure S8A). Overall, the mappability of transcripts and their 3′ ends seems comparable with the most common change being from medium mappable transcripts to high mappable 3′ ends and few extreme cases (e.g., high mappability transcripts with low mappability 3′ ends), see Additional file 1: Figure S8B.

In line with the higher mappability, our simulated 3′ end sequencing data also showed higher mapping accuracies across all conversion rates and mappability classes. When considering FCR estimations, however, full-length sequencing showed the smallest deviation from the simulated FCR, implying that the larger mapping space of the full transcript allows for more robust FCR estimates (Additional file 1: Figure S8C and D). Mapping 3′ end data to the transcriptome showed the worst performance with noticeable differences to simulated values already for high and medium mappability genes. Mapping the same data to the genome in an unbiased way performed clearly better and adding “noise” (i.e., reads from “internal priming” events) even seemed to have a beneficial effect. We speculate that here both converted and unconverted FP reads are mapped at the same ratio as the TP 3′ end reads, therefore making the recall of the FCR more robust despite stemming from FP signal. We concluded that the overall mapping performance in a reduced sequence space does not strikingly aggravate mappability issues; however, there is a robustness trade-off for biological measures such as FCR. This is best mitigated by using a genome-mapping approach that offers more mapping space to reads that would otherwise potentially falsely be assigned to transcript 3′ ends when restricting the mapping space to transcript sequences only.

### A mosaic sequence alignment approach enhances the interpretation of metabolic labeling data with implications for RNA stability measurements

**Fig. 2:**
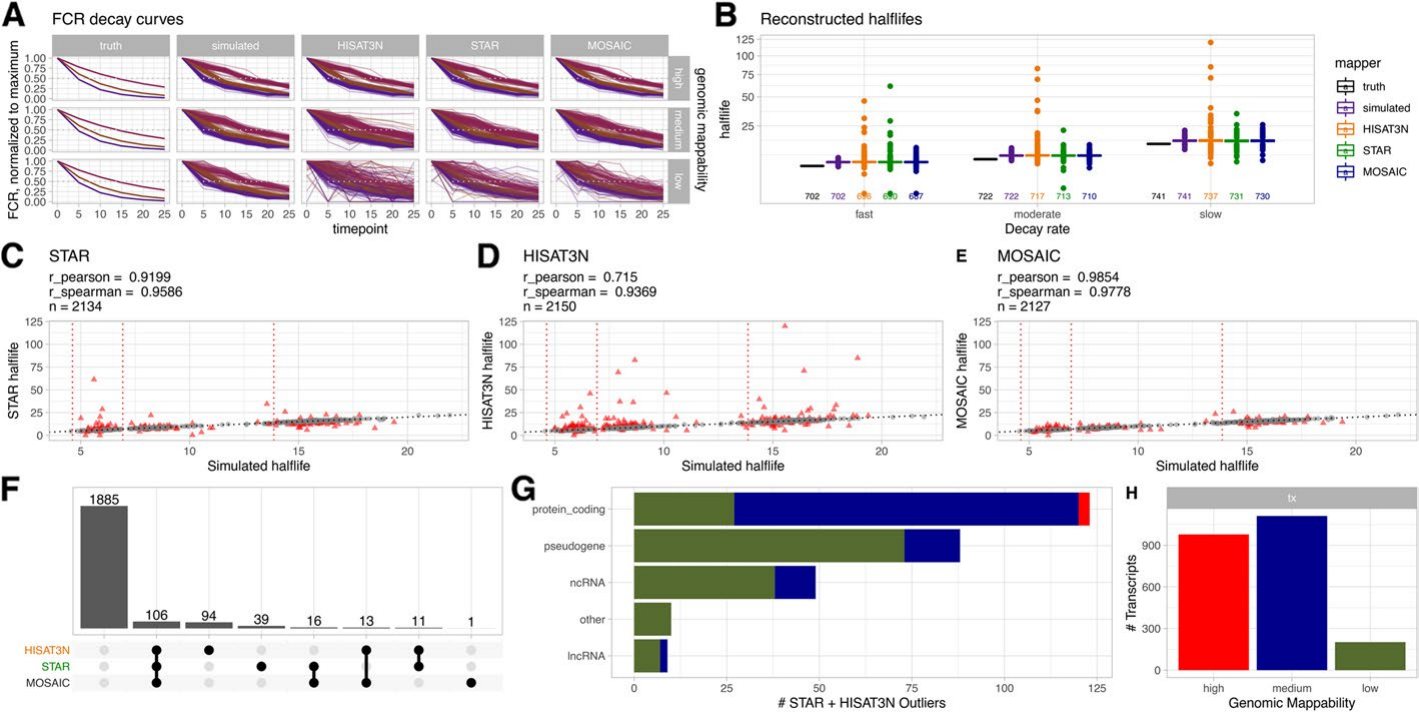
Effect of NC on transcript half-life reconstruction (corresponding plot for introns in Additional file 1: Figure S9). **A** Normalized, mature transcript FCR per time point (arbitrary units) for true, simulated, and mapped data. The truth data models an idealized exponential decay curve for three randomly assigned decay rates (violet: fast/*k* = 0.15, brown: moderate/*k* = 0.1, magenta: slow/*k* = 0.05). FCR for simulated and mapper-specific alignments was estimated as explained in the “Methods” section. The mosaic panel was created by choosing the mapper-based FCR estimate closest to the simulated value per transcript. FCR was normalized to the maximum value across all timepoints. The data reconstructed from the read mapper alignments show increasing noise with decreasing mappability although some clear outliers are also visible in high mappability regions. Grey, dashed lines indicate 50% FCR. **B** Reconstructed half-lives per decay rate. The box plots show a considerable number of outliers for both mappers; numbers of considered transcripts are plotted below the boxes. See main text for a discussion why reconstructed half-lives from simulated data are systematically higher than the true value (black boxes). **C**–**E** Correlations between estimated half-lives from simulated data and STAR, HISAT-3N, and “mosaic” data, respectively. Transcripts with > 10% difference to simulated half-lives were considered outliers and are indicated by red triangles. Theoretical true half-lives per decay rate group are indicated by red dashed lines. **F** Upset plot showing numbers of outliers (including transcripts for which no half-life could be estimated) shared by mapper and mosaic data respectively. **G** Gene types of outliers (in one or both mappers), colored by mappability (see **H** for color code). Most outliers were transcripts of protein coding genes. **H** Number of transcripts per mappability category for the analyzed gene set

Intrigued by the observed NC-dependent FCR differences, we simulated metabolic labeling pulse-chase data to estimate the effects of reduced NC mapping accuracies on the downstream analysis of RNA decay half-life reconstruction [4, 25]. In a metabolic labeling pulse-chase experiment, cells are typically exposed to a nucleotide analog (e.g., 4-thiouridine) for a considerable time span to ensure a fully labeled RNA population. Then, the labeling nucleotide analog is washed out and RNA is extracted at multiple consecutive time points. Extracted RNA is finally exposed to nucleotide conversion chemistry followed by cDNA library preparation and sequencing. FCR per time point is determined and normalized. A decay model (typically exponential decay is assumed) is fitted to these data and half-lives are derived which are interpreted as a quantification of RNA stability. Accordingly, we configured *splice_sim* to simulate unlabeled/labeled (5% T-to-C conversion rate) RNA ratios over multiple timepoints following a simple exponential decay model for three different decay rates (fast, medium, slow). We included ~ 2.3k mature transcripts (of which 2150 were included in this analysis after filtering for minimum transcript length > 100 bp) and their ~ 17 k introns that are expressed in mouse embryonic stem cells (see Additional file 1: Supplement). After simulation, read mapping, and counting, we calculated FCR per transcript/intron and time point and fitted an exponential decay model (FCR~${e}^{t \times -k}$ where *t* is time and *k* is the decay rate constant) to these data and reconstructed half-lives (Fig. 2A, B). Note that estimated half-lives from simulated data are systematically higher than the true value (cf. Fig. 2B). This is because in this analysis, as in a true world scenario, all reads without a found NC were considered to be stemming from “new” (after washing point) RNA, including a considerable number of reads stemming from “old” RNA that have zero conversions just by chance. Although we could have corrected for this in our simulated data (as we know the origin of each individual read), we decided to treat simulated and mapped data the same way to keep them comparable.

Although half-life estimation was robust for most transcripts and introns (Fig. 2 and Additional file 1: Figure S9), we observed a considerable number of outliers with more than 10% difference to simulated half-lives for both mappers in the medium and low mappability segments. Those outliers over- and underestimated simulated half-lives of > 120 protein coding genes (Fig. 2C–G). Again, we applied a “mosaic” approach by choosing FCR values closest to simulated values from mapper-specific data per transcript. This resulted in fewer outliers and smaller differences to simulated half-lives (Fig. 2E). Most outliers (106) were shared; however, a considerable number were found exclusively in HISAT-3N (94) and STAR (39) alignments. The “mosaic” approach removed 144 outliers while adding only one additional one (Fig. 2F). Interestingly, HISAT-3N produced worse fits to the decay model as supported by lower observed Efron pseudo-*R*^2^ values (Additional file 1: Figure S10; the “Methods” section) and consequently also more half-life outliers for this dataset although it showed better overall FCR reconstruction compared to STAR (Fig. 1E). We found comparable numbers of outliers across all three simulated decay rates.

### Intron filtering improves isoform mix estimates of low mappability transcripts

**Fig. 3:**
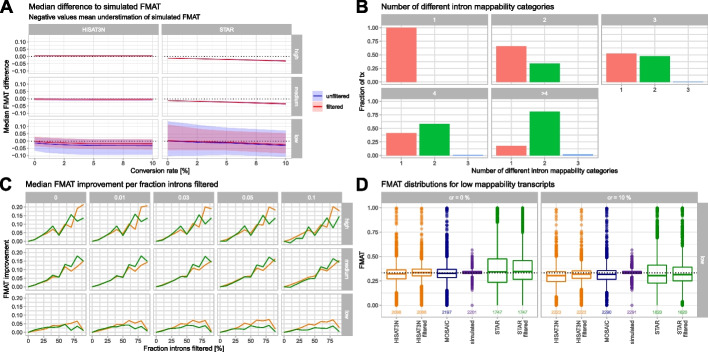
FMAT reconstruction. **A** Median difference to simulated FMAT for unfiltered and intron-filtered data (shaded areas show interquartile ranges); negative values mean underestimation of simulated values. Intron filtering is described in the main text and improves results particularly for HISAT-3N in the low mappability segment. STAR shows larger underestimation with increasing conversion rates indicating difficulties to map spliced reads with more NCs. **B** Fractions of transcripts with different intron mappability categories, stratified by number of introns. Most transcripts with more than 3 introns contain introns from different mappability categories. **C** Median FMAT improvement increases with higher fractions of filtered introns per transcript. HISAT-3N seems to profit more in the low mappability segment. **D** Distributions of simulated, mapper specific, intron-filtered, and mosaic FMAT values for low mappability transcripts and 0 and 10% conversion rates. The dotted black line indicates the theoretical value of ${}^{1}\!\left/ \!{}_{3}\right.$ (cf. the “Methods” section); numbers of observations are plotted below the boxes. Intron filtering and a “mosaic” approach improve FMAT estimations, and the “mosaic” approach recovers data for more transcripts. A respective human metabolic labeling data plot is provided in Additional file 1: Figure S16 for comparison

Alignment of spliced reads is particularly difficult as it needs to take the possibility of (typically large) gaps due to spliced out introns into account and requires the accurate placement of short (sometimes single nt) sub-sequences of reads (anchors) that span over these gaps [10, 26, 27]. This process is expected to be particularly sensitive to additional mismatches introduced by NC. To assess the influence of low-frequency NCs on mapping accuracies of spliced reads, we counted spliced (stemming from mature gene isoforms) and all informative (spliced and donor/acceptor spanning) reads per splice junction (SJ) and calculated fractions of mature isoform reads (FMAT; fraction mature isoform, Table 1) per SJ and transcript, a metric that is typically used in downstream analyses. We observed that differences between mapper-reconstructed and simulated FMAT values increase with decreasing mappability and, for STAR, also with conversion rate (Fig. 3A). Difference to simulated FMAT values correlated negatively with our F_1_ values as expected (Additional file 1: Figure S11). When looking closer at the distribution of FMAT values within genes, we observed that transcripts are often a mosaic of high, medium, and low mappability introns (Fig. 3B). We reasoned that FMAT reconstruction of whole transcripts would benefit from filtering introns with low NC mapping accuracies that pollute the overall signal. We filtered introns based on the observed difference to simulated FMAT (see the “Methods” section and Additional file 1: Figure S12 and 13 for examples) and compared filtered with original FMAT values. Intron filtering decreased differences to the true FMAT values and reduced the overall negative correlation with F_1_ values (Fig. 3A and Additional file 1: Figure S11). Figure 3C shows that the improvement due to intron filtering is highest if large fractions of introns were omitted and that HISAT-3N profits more than STAR in low mappability regions. Consequently, we observed a clear improvement in FMAT reconstruction for filtered data when plotting value distributions of low mappability transcripts (Fig. 3D). Additionally, we again tried a “mosaic” approach by choosing the mapper with the most accurate FMAT value per intron which also improved overall estimations and recovered data for more transcripts compared to the intron filtering approach.

A priori knowledge about true splice junctions considerably improves accuracy of spliced read mapping [28]. To confirm this in our data, we repeated our simulations without passing respective gene model information to the read mappers and found a strong increase in FN spliced reads as well as FP SJ overlapping reads and in consequence a strong underestimation of simulated FMAT values respectively large numbers of introns filtered by our approach (Additional file 1: Figure S14). This underlines the importance of feeding accurate information about known and/or suspected splice junctions to splice-aware read mappers but should also motivate in silico experiments to learn about the expected readout for the detection of novel splice junctions from RNA-seq data. A further analysis of SJ detection in our main dataset unveiled that HISAT-3N, when compared to STAR, recovered a higher fraction of the (passed) known SJ while at the same time also reporting a higher number of novel SJs which are per definition false-positive in our dataset (Additional file 1: Figure S15). Notably, we observed increasing false-positive SJs with increasing conversion rates for both mappers.

### Low mappability regions are hotspots of false cytosine methylation calls

Next, we configured *splice_sim* to simulate RNA-BS-seq data which is characterized by high C-to-T conversions rates (98% in our simulation). We used these data to evaluate mapping accuracies of HISAT-3N and meRanGs, a specialized 3N bisulfite RNA-seq read mapper based on STAR. First, we compared overall mapping accuracies in the presence and absence of NCs and found F_1_ scores similar to our metabolic labeling dataset. Comparison of the two aligners revealed that meRanGs performed comparably to the general-purpose NC aligner HISAT-3N (Additional file 1: Figure S17A). Interestingly, we observed a slight drop in accuracy for HISAT-3N for the NC dataset but no such drop for meRanGs. We speculate that this drop could be due to the ~ 2% HISAT-3N reads mapping to the wrong strand (Additional file 1: Figure S2E). Since we stratified our data using genomic mappability scores (umap), we were curious if stratification by methylome mappability scores as presented in [19] led to better predicted mapping accuracy. For this purpose, we compared umap (general mappability) and bismap (methylome mappability) scores and found a high positive correlation (Additional file 1: Figure S17B). We then quantile-normalized bismap scores before creating equally sized mappability class bins for a direct umap to bsmap comparison. Notably, when calculating ΔF_1_ values between mappability classes, we did not find any striking difference between the two mappability scores and no clear-cut winner when comparing different mappers, features, or mappability classes (Additional file 1: Figure S17C and D). We therefore conducted our analysis using the same (umap-based) genome mappability scores as for the metabolic labeling analysis.

**Fig. 4:**
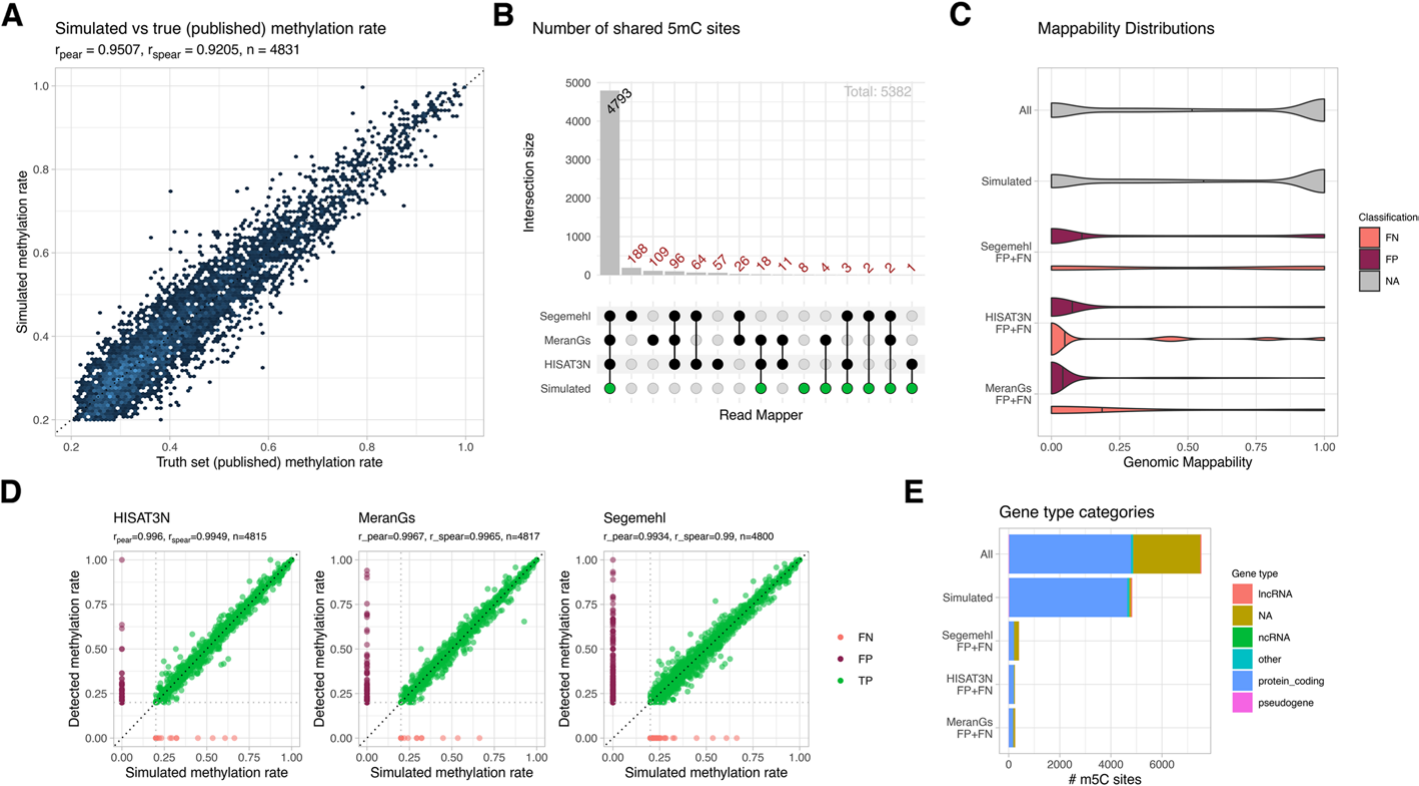
Effect of (NC) mappability on methylation site reconstruction. To estimate the impact of (NC) mapping biases on methylation site calling, we simulated RNA-BS-seq data for transcripts overlapping 4831 published mESC m^5^C sites (see Additional file 1: Supplement for details). **A** High correlation of published (“truth”) and simulated methylation rates. **B** Upset plot showing the number of simulated m^5^C calls recovered from HISAT-3N, meRanGs and Segemehl alignments. Calls were classified with respect to the simulated sites as TP/FP/FN. **C** FP and FN calls were predominantly located in regions with low mappability. **D** Methylation rate correlations for HISAT-3N, meRanGs, and Segemehl. Note that these plots also contain methylation rates for FN (light-red) and FP (dark-red) calls but shown correlation coefficients were calculated from TP calls (green) only. All three mappers produced a significant number of FPs, several of them shared (cf. 4B), as well as a few FN calls. Example calls are depicted in Additional file 1: Figure S18. **E** False calls were located predominantly in protein coding genes

We then set out to measure the effects of mismapped NC reads on calling m^5^C sites in a realistic dataset. For this, we spiked a published set of mESC m^5^C sites with methylation rates (per site) ranging from 20 to 100% into a *splice_sim* dataset with 1910 overlapping transcripts and measured how many of them could be recalled (TP), how many were missed (FN), and how many false-positive (FP) calls we would get. A detailed description of this analysis that included the mappers HISAT-3N, meRanGs, and Segemehl is provided in Additional file 1: Supplement. Overall, simulated and true methylation rates correlated well and most m^5^C sites were re-called in all mapper-derived datasets (4793/4831 = 99.2%, Fig. 4A, B). All mappers did, however, produced considerable amounts of FP and a few FN m^5^C calls, mainly in low mappability regions of protein coding genes (Fig. 4C–E). When inspecting the data, we found that FP and FN m^5^C sites were mainly a result of incomplete (simulated) bisulfite conversion (i.e., not all reads contained a C-to-T NC at a true m^5^C site) and missing FN reads in low mappability regions (Additional file 1: Figure S18), in line with reported experimental artifacts of the BS-seq protocol [29–32]. False positives were called with methylation rates over the whole range (20–100%) which makes such calls not straightforward to filter. Although advanced filtering approaches (e.g., by in silico folding of transcripts and checking for the base-pairing status of potential m^5^C sites [6, 33]) would likely reduce false calls, our analysis clearly shows that regions of low genome mappability are hotspots of false m^5^C calls and should be handled with particular care.

### Genome-specific features impact NC RNA-seq data analysis in different species

Finally, we repeated our analysis with human data (GRCh38, canonical Ensembl genes) using the same configuration parameters as for the mouse data experiments for comparison and reference (Additional file 1: Table S2). Note that when comparing genome mappability distributions between our selected human and mouse annotations, we found slightly but significantly increased mappability for mouse annotations and more human transcripts in the medium mappability category (Additional file 1: Figure S19). We found, however, also less low mappability transcripts in the human annotations. Human exon/intron annotations were slightly shorter/longer respectively when compared to mouse annotations. Overall, the human data showed very similar results when compared to our mouse datasets with differences possibly explained by the abovementioned difference in mappability distributions.

## Discussion

We presented *splice_sim*, a versatile RNA-seq simulation and evaluation framework, and used it for a comprehensive analysis of annotation-based mapping accuracies of regular as well as NC reads that focused on potential effects on downstream analyses. Overall, our analysis revealed that mapping accuracies with and without NC are high (F_1_ > 0.98) for all considered mappers when considering annotations with high/medium genome mappability but substantially lower (F_1_ < 0.55) for low mappability ones, a considerable fraction that includes protein coding as well as regulatory RNAs of biological importance (Additional file 1: Figure S1 [12]). Particularly in regions with low genome mappability, we observed considerable differences in mapping accuracies among groups of unmodified and NC reads that consequently lead to increased error rates in measures based on the comparison of such read groups (e.g., FCR). Other than for many metrics derived from regular RNA-seq data (e.g., relative abundances), NC mapping biases do not cancel out and we demonstrate that they can lead to wrong estimates of downstream measures (transcript half-lives or isoform estimates) which in turn affect biological implications/interpretations.

Our result tables for mouse and human metabolic labeling and RNA-BS-seq data, including raw counts, performance measures, and categorical data, are built on GENCODE annotations and published alongside this manuscript. A summary plot of these tables is provided in Additional file 1: Figure S26: for each transcript, we calculated which of the evaluated mappers showed the best performance with regard to general mapping accuracy, FCR and FMAT reconstruction, reporting both mappers if we observed only small differences and none if we considered the differences to the true values too large for a useful analysis. We demonstrate how our results can guide data cleaning and analysis strategies: using simple approaches such as best mapper selection (e.g., in a “mosaic” approach) or filtering of identified problematic introns, we were able to improve overall accuracy of derived measures and thereby biological interpretability. The demonstrated accuracy gains due to “mosaic” analysis strategies demonstrate that the evaluated mappers differ to some extent in the made mapping errors due to their differing alignment algorithms/approaches, and overall, our evaluation does not render one of them superior to the others. In practice, a “mosaic” analysis strategy, however, requires the generation of (at least) two alignments per dataset thereby increasing analysis costs. As an alternative, intron filtering can be used to improve FMAT predictions as demonstrated. Our datasets furthermore provide information about which transcripts cannot be analyzed with respect to what measures reliably with short read data and should be handled with care or omitted from analysis.

We provide fine-grained precompiled result sets for all annotated transcripts of the GENCODE annotation that are useful for benchmarking read mappers on NC data sets. Users can directly look up how a given mapping tool will perform on their genomic feature of interest and take countermeasures to mitigate bad estimates from problematic regions. Users are also encouraged to apply our software and analysis scripts to calculate mapping accuracy data for alternative model organisms and annotation sets and devise new data cleaning and filtering strategies. While our study considered the entirety of the (theoretically) expressed mouse and human transcriptomes, *splice_sim* is applicable to arbitrary genomes and genomic annotations, thereby also allowing to study NC mapping accuracies in non-coding regions of the genome.

*Splice_sim* can also be used to measure the potential impact of sequencing protocols and analysis pipelines on read mapping accuracies and downstream measures, thereby helping to develop best practices for experiments and data analysis. *Splice_sim* was, for example, used to study and quantify the influence of read length on mapping accuracy (Figure S29), which showed that longer reads increase mappability along all categories but show the same characteristics as their shorter counterparts. In another analysis, we compared datasets with and without passing known splice sites to the read mappers and confirmed the large impact of this knowledge on spliced read mapping accuracy (Fig S13-15). We also demonstrated that 3′ end sequencing is a valid alternative to whole-transcript sequencing, which has advantages for certain applications, such as FCR estimation, and showed that it is beneficial to map 3′ end reads to the whole genome to get most accurate FCR estimations (Fig S7-8). Finally, we demonstrated that low mappability regions are hotspots of false m^5^C calls that are not straightforward to filter based on measured methylation rates (Fig. 4). Besides experimental optimizations of RNA-BS-seq protocols, *splice-sim* can thus help to establish accurate and reproducible sets of true m^5^C sites in mammalian transcriptomes which were reported with large variation (ranging from < 100 to > 10 k sites) in current literature [7].

## Related work

*Splice_sim* distinguishes itself from previous RNA-seq simulators by its ability to simulate mixtures of regular and NC reads and its evaluation module that provides users with detailed mapping accuracy assessments and additional resources (such as read highlighting and BAM files containing misaligned reads) that are useful for subsequent processing/analyses [34, 35]. *Splice_sim* supports arbitrarily complex isoform mixes, comparable to Polyester [36], and uses ART [37] for the actual simulation of (unmodified) high-throughput sequencing reads, but this module could easily be replaced by alternative RNA-seq simulators (e.g., Camparee [38], BEERS [39], or RSEM [40]). Notably, splice*_sim* quantifies mapping accuracies for entities of direct biological interpretation (e.g., exons and introns) instead of general genomic regions as most previous work on genome mappability to which our work is complementary [15, 19, 41].

## Limitations

There are some limitations to our chosen approach for estimating mapping accuracies: first, our method is based on simulations as it is obviously unfeasible to iterate all possible NC reads which could lead to biases due to stochastic effects. We are, however, confident that stochastic effects would be rather small based on our analysis of three replicates that showed very high correlation (Additional file 1: Figure S22).

Second, for our main dataset (***m_big***), we configured *splice_sim* to simulate one transcript for each annotated mouse gene with similar read coverage which also means that reads from all transcripts can be mismapped and potentially contribute to false-positive counts. Our results should thus be interpreted as worst-case scenarios in this regard, and we encourage users to repeat our analysis with a configuration that better reflects transcript expression levels in their cell-type of interest (e.g., estimated from standard RNA-seq data) to avoid this bias. Comparing our data to a smaller dataset containing only transcripts that are actively transcribed in mESC, however, showed high correlation and only small differences that could be attributed to inflated FP counts (Additional file 1: Figure S23). We also investigated what fraction of FP reads change their labeling status (i.e., from labeled to unlabeled or vice versa) due to misalignment that introduces/masks NCs but found this to be a minor problem (Additional file 1: Figure S24).

Third, *splice_sim* is currently based on single-end reads only, a configuration that is widely considered as a cost-effective option for standard and NC RNA-seq experiments. Nevertheless, paired-end data would have two central advantages in the discussed experimental settings: first, it would arguably improve overall mappability as both mates would contribute to overall (fragment) mappability. Second, overlapping mates could be used to correct for sequencing errors in the overlapping regions [42]. We therefore plan to extend our software to support such scenarios in the future.

Fourth, *splice_sim* employs Bernoulli processes for simulating NC, a method we deem suitable for RNA-BS-seq and SLAM-seq data (see Additional file 1: Figure S30). Different simulation approaches might, however, be necessary when investigating sequence-context specific NC patterns (e.g., A-to-I RNA editing mediated by the ADAR enzyme family [43]), complex mutational signatures as found in FFPE-derived DNA [44], or extensive depurination and cytosine deamination as observed in ancient DNA [45]. To enable researchers to implement more sophisticated and domain-specific simulation methods, we have designed the NC simulation component of *splice_sim* as a versatile Python method. This method, providing access to genomic coordinates and configuration parameters of simulated reads, is readily adaptable for diverse and domain-specific simulation needs.

## Conclusions

Our study demonstrates how minor differences in mapping accuracy between regular and nucleotide-converted reads may cause considerable numbers of outliers and false calls in downstream measures with direct biological interpretation, such as RNA stabilities or post-transcriptional methylation site calls. We offer a comprehensive simulation and evaluation framework along with simulated datasets and respective analyses that quantify these mapping biases with genomic mappability and selected read mapper being main determinants. Our findings not only elucidate the extent of this issue but also aid in developing effective mitigation strategies, and we demonstrate how a simple “mosaic” selection strategy based on mapper ensemble data can enhance overall data accuracy.

Our simulation and evaluation pipeline, along with the accompanying datasets, can directly be used for filtering and/or correction of experimental data as documented, thereby improving overall accuracy and reliability of derived biological interpretations. *Splice_sim*’s applications encompass a range of functionalities: (i) assessing the effects of nucleotide conversions and read lengths on mapping accuracies, (ii) evaluating read mapper configurations and sequencing approaches (such as whole transcriptome versus 3′ end sequencing), (iii) refining data quality by excluding low-accuracy transcripts, exons, or introns, (iv) employing a “mosaic” approach to select data based on optimal mapping accuracy, and (v) generating test datasets for new methodological developments in nucleotide conversion (NC) research. We also provide an R Markdown (Rmd) script with the *splice_sim* source code to demonstrate these applications.

## Methods

*Splice_sim* is implemented by a set of Python and R scripts that are orchestrated by nextflow pipelines [46]. The complete software stack is bundled in a Docker container to increase reproducibility and usability. A detailed description of *splice_sim* is provided in Additional file 1: Supplement (Additional file 1: Figure S20 and 21). Briefly, it simulates short reads with realistic sequencing errors for a set of configured transcript ids and isoforms and injects NCs with given conversion rates and configurable sets of SNVs with given variant allele frequencies (VAF). For our main evaluation dataset (***m_big***), we simulated 1:1 ratios of premature (unspliced) and mature (fully spliced) isoforms for > 50k mm10 (GRCm38) transcripts with five different conversion rates (0, 1, 3, 5, 10%). We simulated three replicates and mapped the reads with STAR and HISAT-3N. Mapped reads were classified as true positive (TP), false positive (FP), or false-negative (FN) with respect to given genomic features of interest (exons, introns, full transcripts, and splice junctions) by comparing to the simulated data (see Additional file 1: Supplement for a detailed description of this procedure). Resulting count tables were grouped by read mapper, conversion rate, annotation feature id, originating isoform, reads with at least 1/at least 2 NCs, and reads with at least 1/at least 2 simulated sequencing errors. Data was annotated with additional meta-data (e.g., GENCODE gene types) as required and analyzed in RStudio v2022.02.1. For estimating genome mappability per feature, we downloaded umap mm10/hg38 single-read k24 tracks from https://bismap.hoffmanlab.org and calculated mean values over annotation feature intervals. Features were then classified into three mappability categories: high (mean value > 0.9), low (< 0.2), and medium.

Mapping performance per annotation (transcript, exon, intron, splice-junction) was measured by precision ($\frac{\text{TP}}{\text{TP}+\text{FP}}$), recall ($\frac{\text{TP}}{\text{TP}+\text{FN}}$) and accuracy using the F_1_-measure: ${F}_{1}=2\times \frac{\text{precision}\times \text{recall}}{\text{precision}+\text{recall}}$ = $\frac{2\times \text{TP}}{2\times \text{TP} + \text{FP} + \text{FN}}$. Fraction of converted reads per annotation was defined as the ratio between NC containing reads and all reads, $\text{FCR}=\frac{\#\text{converted}-\text{reads}}{\#\text{all}-\text{reads}}$. All reads with at least one NC were considered converted. Fraction of mature isoform per transcript was calculated as

$$FMAT=\frac{\#mature-isoform-reads}{\#mature-isoform-reads+\#premature-isoform-reads}=\frac{\Sigma \#spl-reads}{\Sigma \#spl-reads + \Sigma \#don-reads + \Sigma \#acc-reads}$$

where *spl*, *acc*, and *don* are intron splicing, donor overlapping, and acceptor overlapping reads respectively. Given the configured 1:1 ratio between mature and premature isoforms, we expected a theoretical FMAT value of ${}^{1}\!\left/ \!{}_{3}\right.$ and recovered a mean value of 0.334 from our simulated data (cf. Fig. 3D). Intron filtering per transcript was implemented as follows: we first sorted introns by decreasing FMAT difference to the simulated data and consecutively filtered introns with a difference greater than 10% until no more such introns were available or until removal of the next intron would lead to less than 100 remaining informative (spl + don + acc) reads for this transcript.

For the RNA decay experiments, we simulated mature (for transcript decay rates) and premature (for intron decay rates) isoforms for six timepoints with arbitrary units and 5% T/C conversion rate. The fraction of labeled and unlabeled RNA per time point was configured in a way that the resulting FCR values follow a simple exponential decay model $\text{FCR}\sim {e}^{t \times -k}$ where *t* is time and *k* is the decay rate constant. We simulated data for ~ 2.3k transcripts with three randomly assigned decay rates (fast/*k* = 0.15, moderate/*k* = 0.1, slow/*k* = 0.05), mapped the reads, annotated T-to-C conversions in the BAM files, and extracted reads with at least one T-to-C conversion to new BAM files. We then counted reads per genomic annotation with featureCounts [47] for complete and T-to-C-only alignments, calculated FCR per transcript and intron, and fitted the data to the exponential model in R. A more detailed description of this procedure is provided in Additional file 1: Supplement. Resulting half-life estimates were compared to the theoretical and simulation-derived values and we compared goodness-of-fit between mappers by Efron’s pseudo-*R*^2^ values that were calculated as $1-\frac{rss}{tss}$ where *rss* is the sum of the squared model residuals and *tss* is the total variability in the dependent variable (Additional file 1: Figure S10).

For the human dataset (**h_big**), we used the same configuration as for **m_big** and simulated data for all GENCODE v39 (GRCh38) transcripts annotated with the Ensembl canonical annotation tag.

For the RNA-BS-seq analysis, we simulated a dataset (**m_big_bs**) containing the same transcripts as **m_big** and 98% C-to-T conversions. For measuring m^5^C calling accuracy, we simulated a smaller dataset (**m_small_bs**) with 1910 transcripts that overlap with a set of published methylated m^5^C sites called from mESC total polyA RNA-seq data (https://pubmed.ncbi.nlm.nih.gov/28077169, GEO project GSE83432 [33]). Published m^5^C sites were spiked into the dataset with given methylation rates; simulated reads were mapped with MeranGs, HISAT-3N, and Segemehl and filtered for reads with mapping quality ≥ 20. Finally, methylation status was called with meRanCall [18] and compared to the truth set. A more detailed description of this analysis is provided in Additional file 1: Supplement.

For the 3′ end analysis, we used the comprehensive full-length transcript dataset (**m_big**) as reference and simulated several 3′ end datasets, taking the last 200 nt ranging from the transcript 3′ ends with different noise levels (see Additional file 1: Figure S7). We then calculated count tables for all scenarios and benchmarked them against the full-length reference set for mapping accuracy and FCR estimation. Applying 3′ end sequencing for FMAT estimation was omitted as only few evaluated 200 nt 3′ ends span a splice-junction. A more detailed description of the 3′ end simulation routine is provided in Additional file 1: Supplement.

Note that we also evaluated the effect of mapping quality filtering (as often seen in bioinformatics analysis pipelines) on estimated mapping accuracies and observed a strong decrease in the low mappability segment as expected as filtered reads are treated as FN by our pipeline (Additional file 1: Figure S25). In high/medium mappability segments, we observed small but noticeable accuracy decreases. Note that *splice_sim* is by default returning counts for unfiltered and mapping quality (MQ > 20) filtered alignments.

Resource benchmarks documenting required computational resources and expected runtimes of our pipeline are provided in Additional file 1: Figure S27 and 28.

## Supplementary Information

Additional file 1: Supplementary Figures and Tables.

Additional file 2. Review History.

## Data Availability

The source code of *splice_sim* is available on GitHub at https://github.com/popitsch/splice_sim under the GPL-3.0 license, and the corresponding execution environment is wrapped in a Docker image available at https://hub.docker.com/repository/docker/tobneu/splice_sim. Mapping accuracy tables, evaluation result files, and pipeline configuration files of all simulated datasets as well as the used analysis scripts are deposited in Zenodo (10.5281/zenodo.11196570 [48]).
