# Supplementary material for: Splice_sim: a nucleotide conversion-enabled RNA-seq simulation and evaluation framework
Source: Genome Biol. 2024 Jun 25;25:166. doi: 10.1186/s13059-024-03313-8 (PMC11514792; doi:10.1186/s13059-024-03313-8)
Supplement: Supplementary file 1 — Additional file 1: Supplementary Figures and Tables. [file 13059_2024_3313_MOESM1_ESM.pdf]

Splice_sim: a nucleotide-conversion enabled RNA-seq simulation and evaluation framework

Supplement

This document contains additional information and figures in support of the analyses conducted in the main manuscript. The following sections provide detailed descriptions of how the various datasets were created and contain quality control as well as supplementary plots that are referenced throughout the main manuscript.

Supplementary Figures 1

Splice_sim 19
- Simulation pipeline 20
- Evaluation pipeline 24

Evaluation Datasets 27
- Mouse nucleotide labelling datasets 28
- RNA-BS-seq analysis 33
- Decay simulations 34
- 3'end sequencing analysis 35
- Human nucleotide labelling dataset 35

Results files 36

Resource benchmarks 37

Supplementary Figures

All following supplementary figures were calculated from the replicated mouse ***m_big*** dataset (see below) unless otherwise indicated in the figure caption.

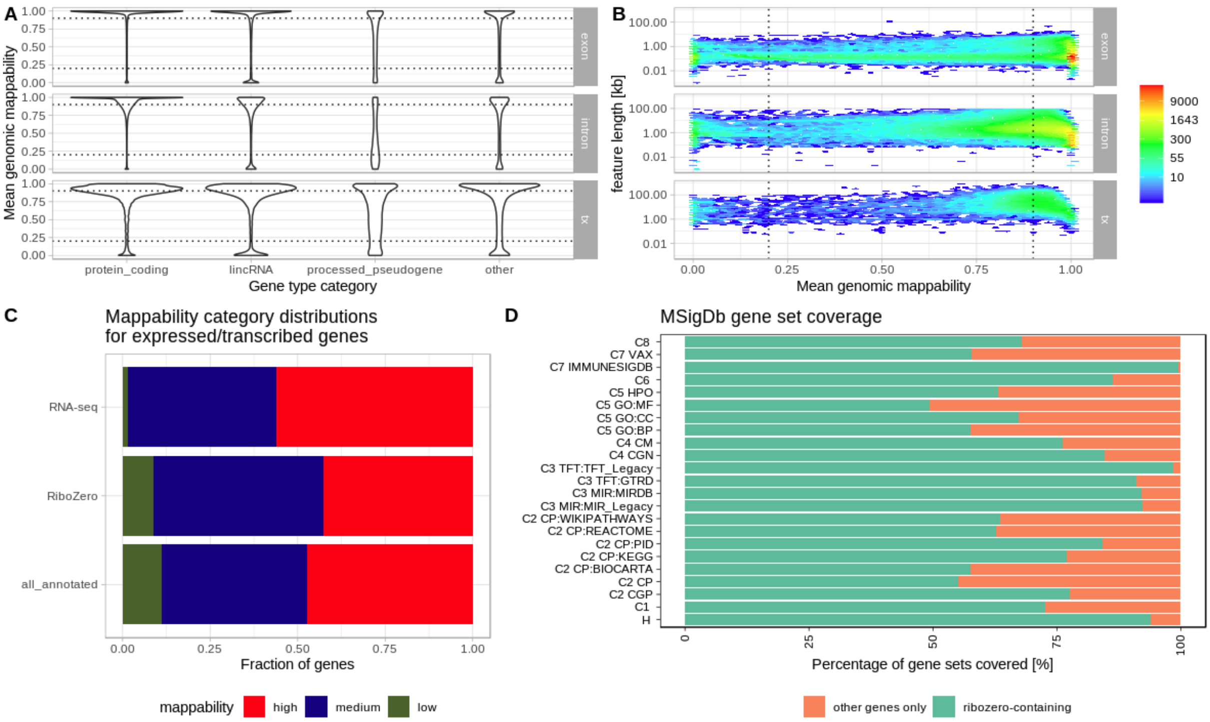

Fig S1: **Regions of reduced genomic mappability occur across all transcript classes and include a significant fraction of (biologically relevant) expressed genes. A** Mean genomic mappability for the mouse mm10 genome per gene type category as annotated in GENCODE, stratified by genomic feature (tx: whole transcript intervals). Dotted lines indicate our selected cutoffs for mappability categories high (>0.9), medium and low (<0.2). **B** Mean genomic mappability vs feature length for protein coding genes. **C** Distribution of mappability categories for expressed genes (TPM>10) in (i) regular RNA-seq data (TPM value tables downloaded from EBI ExpressionAtlas project E-MTAB-6798; plot shows mean category fractions over 94 datasets), (ii) Ribo-zero RNA-seq data from mESC cells, (iii) all annotated transcripts used in this study. Note that ribo-zero data contains a considerably higher fraction of low and medium mappability transcripts (>50%). **D** Fraction of gene sets per MSigDB [1] collection (e.g., H: 'hallmark gene sets') that contain at least one gene from the set of low/medium mappability genes from our RiboZero dataset shown in panel C. The figure shows that a majority of annotated gene sets (e.g., genes annotated in KEGG pathways) contain low and medium mappability genes demonstrating their biological relevance.

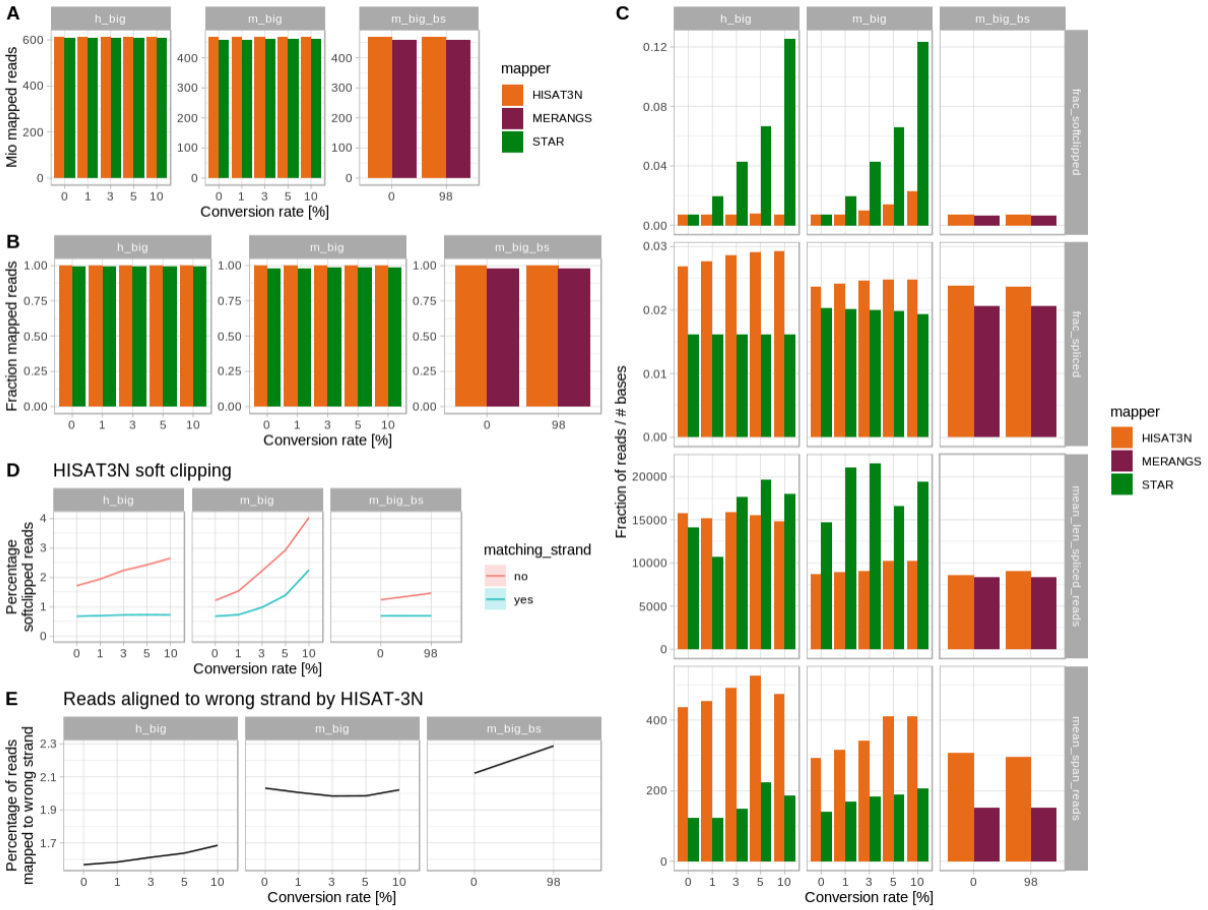

Fig. S2: **Differing spliced read mapping statistics of 3N and 4N mappers. A+B** Mapped reads and fraction of mapped reads per mapper and conversion rate for **m_big** (mean values over three replicates), **h_big** and **m_big_bs**. **C** Splicing statistics per conversion rate and mapper: *frac_softclipped*: fraction of reads with at least one soft-clipped base; *frac_spliced*: fraction of spliced reads; *mean_len_spliced_reads*: mean length of longest N-CIGAR-block per spliced read; *mean_span_reads*: mean (alignment_end - alignment_start +1); These measures were collected per chromosome and replicate and then averaged. STAR makes strong use of soft clipping and the respective read fraction increases with increasing conversion rate as expected due to the increasing number of mismatches. To some extent, this effect can also be observed for HISAT-3N alignments which can partially be explained by reads mapping to the wrong strand (in which case the NC are not properly masked, see panels D+E) but also due to soft-clipping NC bases after 3N mapping at the very read ends. **D+E** The percentage of HISAT-3N soft-clipped reads among reads mapped to the wrong strand is indeed considerably higher (**D**), however, those reads account only for ~2% of all mapped reads (**E**).

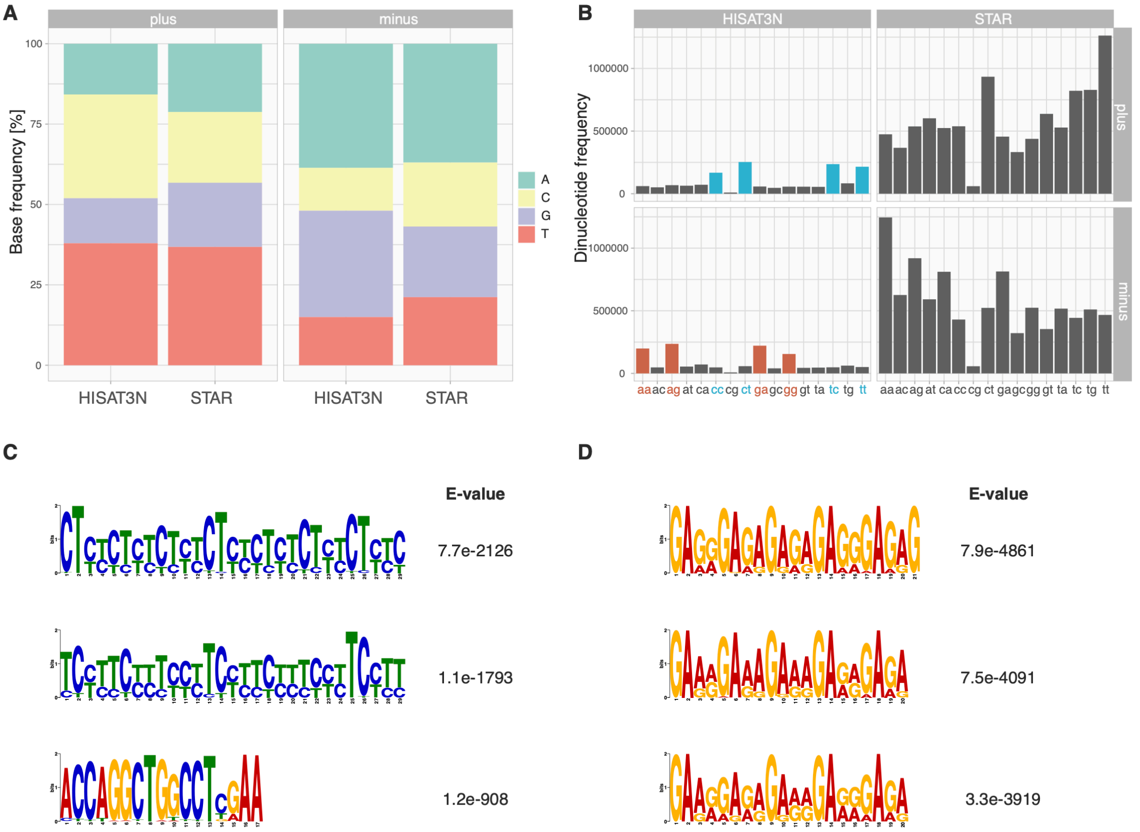

Fig. S3: **Repetitive NC stretches cause false-negative read pileups in 3N alignments.** Regions with a coverage of at least 5 false-negative reads were extracted for all mappers at 0% and 10% conversion rates. Regions exclusive to 10% conversion rate data were extracted by subtracting the 0% control regions and separated by strandedness (HISAT-3N: 15,870 forward and 14,010 reverse strand regions; STAR: 86,616 forward and 86,514 reverse strand regions). **A** Base composition of the extracted regions, note the TC/AG bias for HISAT-3N. **B** Dinucleotide composition for the extracted regions, note the elevated frequencies for all dinucleotides involving TC (turquoise) and AG (red) in HISAT-3N. **C+D** Top 3 motifs called by MEME [2] and their E-values for HISAT-3N forward (C) and reverse (D) strand regions >8 bp.

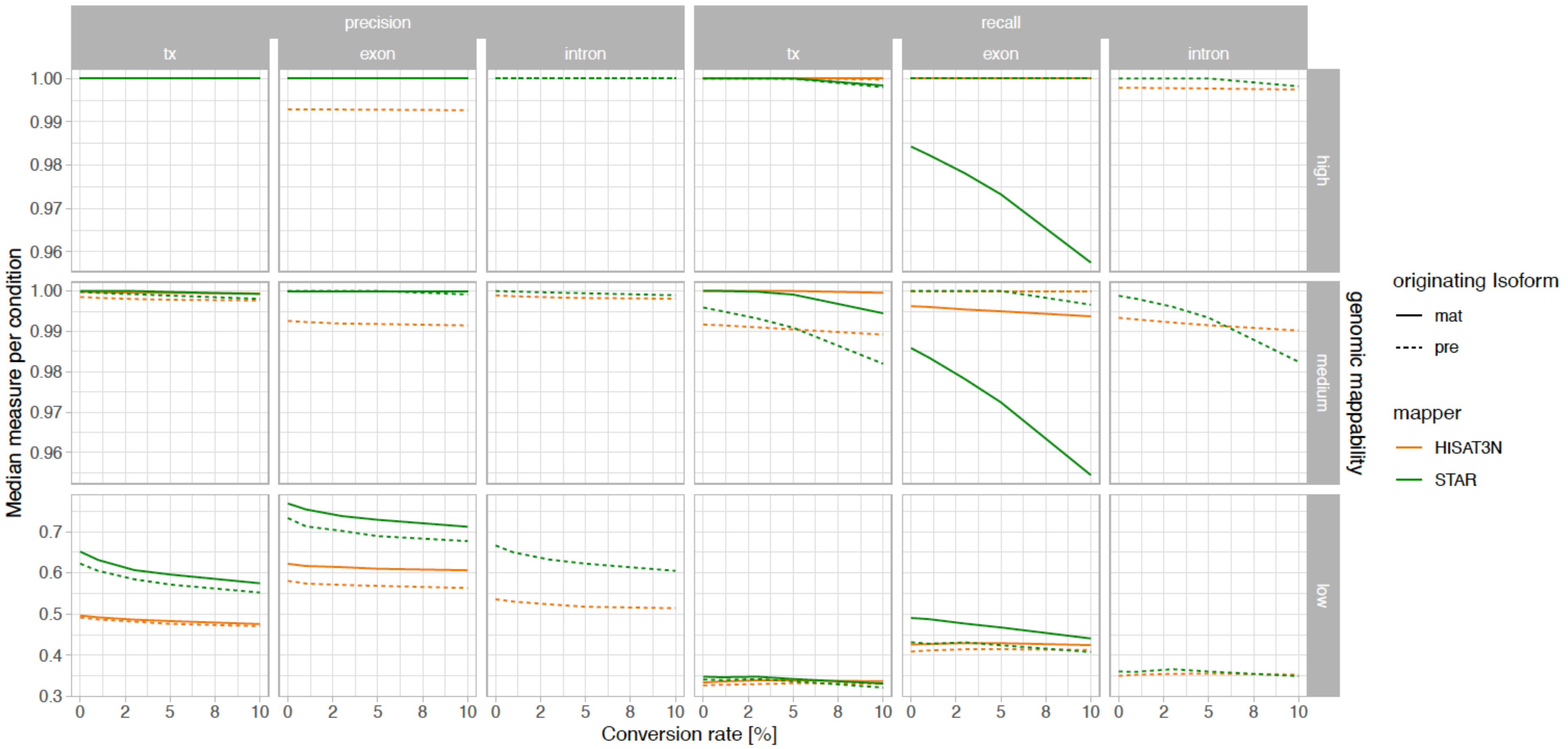

Fig. S4: **Reduced recall is the main driver for reduced mapping accuracy in NC datasets.** Median precision and recall per mapper for different genomic annotations (tx: whole transcript), stratified by originating isoform (pre: premature, mat: mature) and genomic mappability. Plots were created from pooled ***m_big*** data.

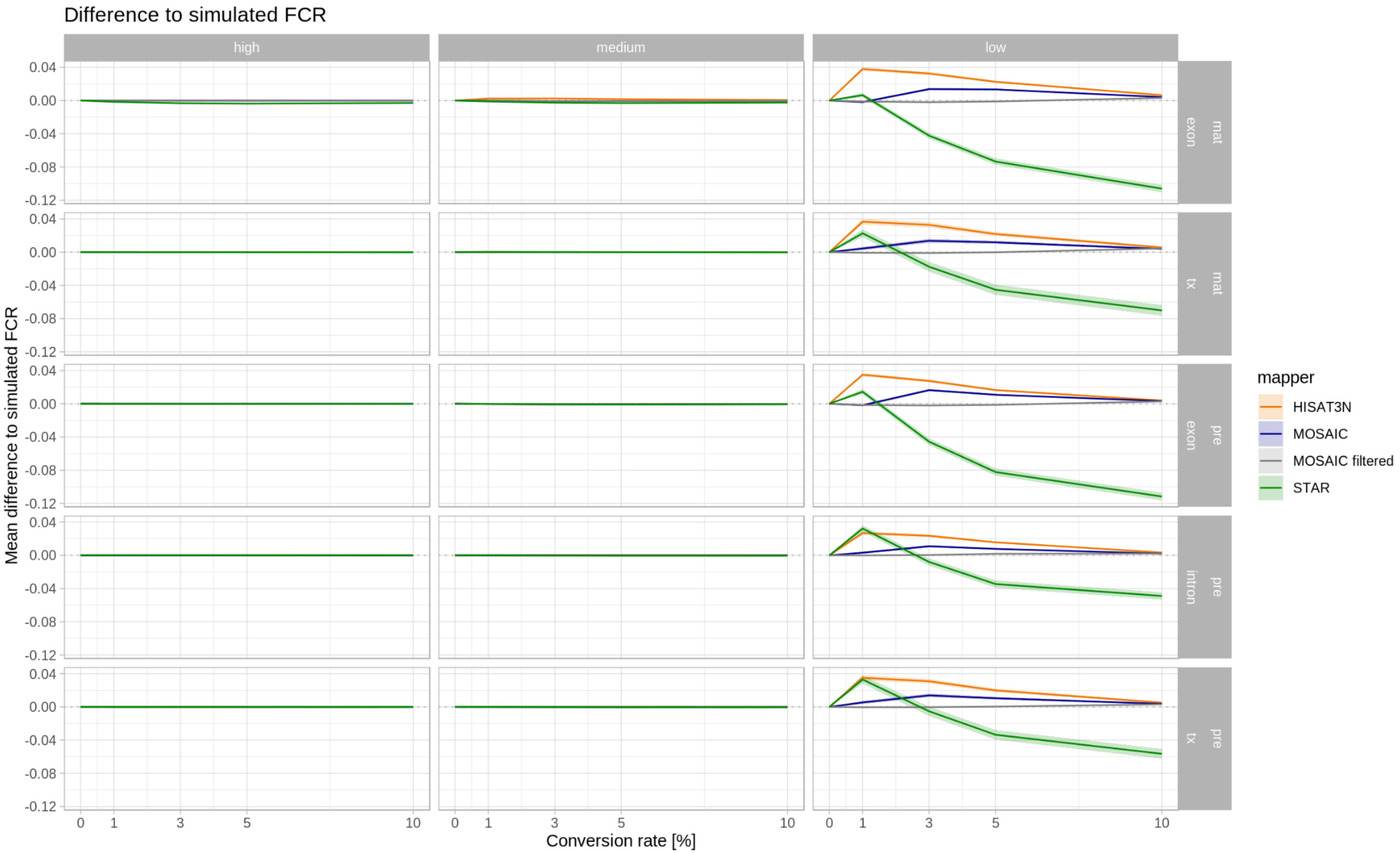

Fig. S5: **Higher error contribution of exonic than intronic FCR estimates.** Mean difference to simulated FCR values (corresponding to main Fig. 1E) for exons, introns and whole transcripts.

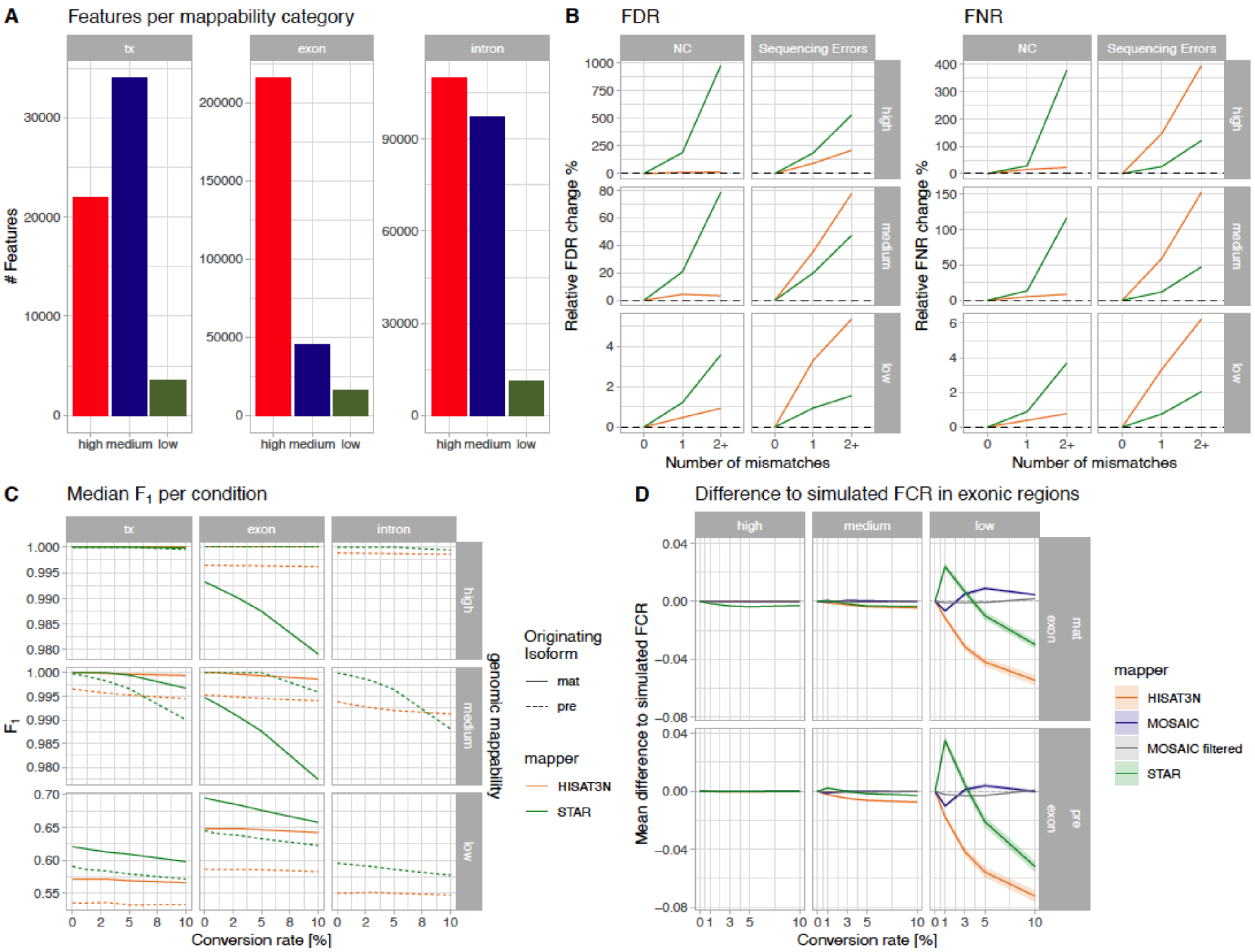

Fig. S6: **Mapping accuracies of the human transcriptome confirm a direct translation of mouse transcriptome findings.** NC mapping accuracies for simulated human nucleotide labelling data (***h_big***, not replicated), analogous to main Fig. 1

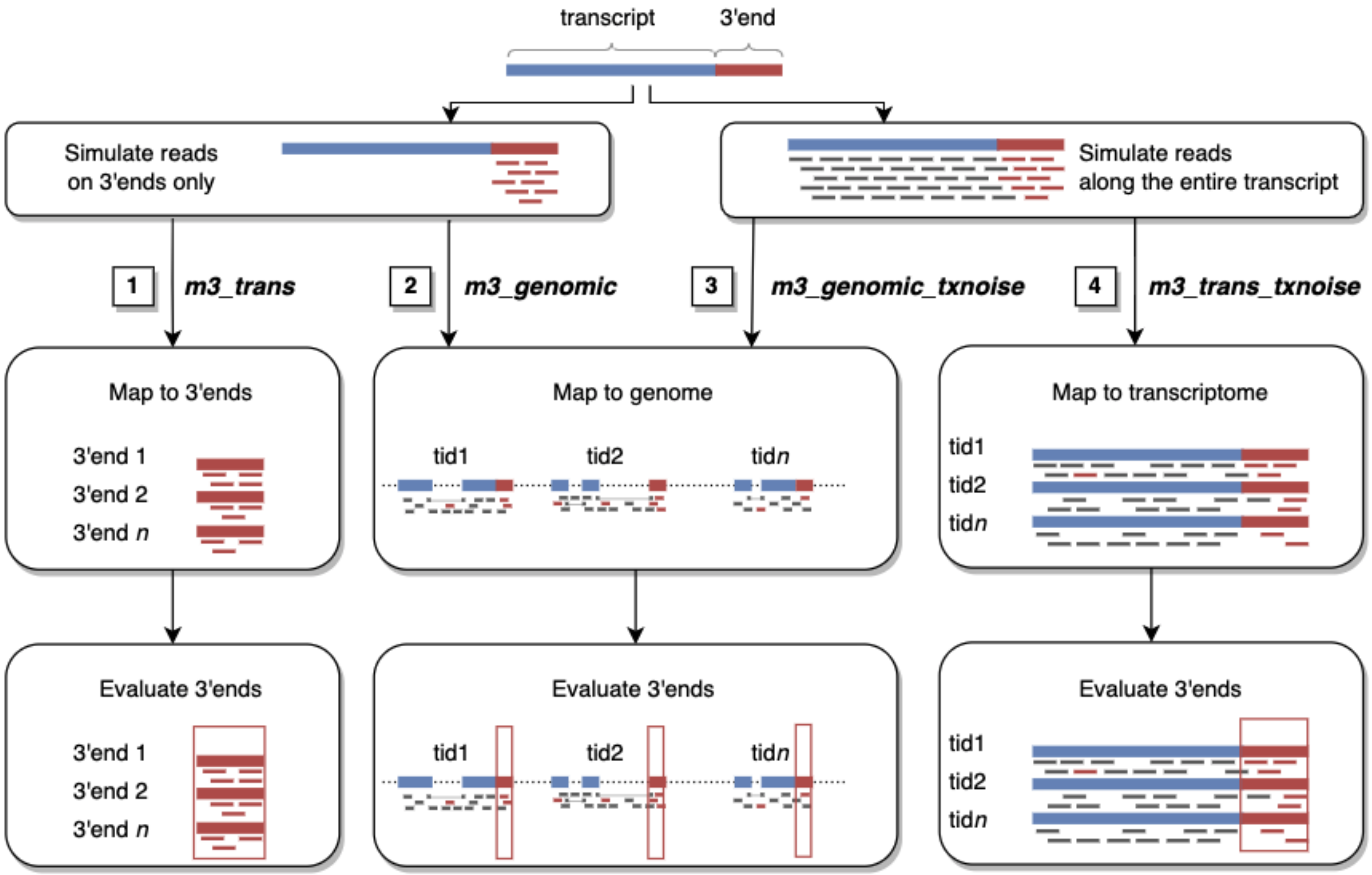

Fig. S7: Block diagram depicting four selected 3'end sequencing evaluation approaches. We either simulated solely 3'end reads (1+2) or assumed complete off-target priming reads from the entire transcript (transcript noise, 3+4). Reads were then mapped either to 3'end sequences (1), whole transcript sequences (4) or the reference genome (with and without transcript noise, 2+3). Final count tables were calculated from 3'end intervals only.

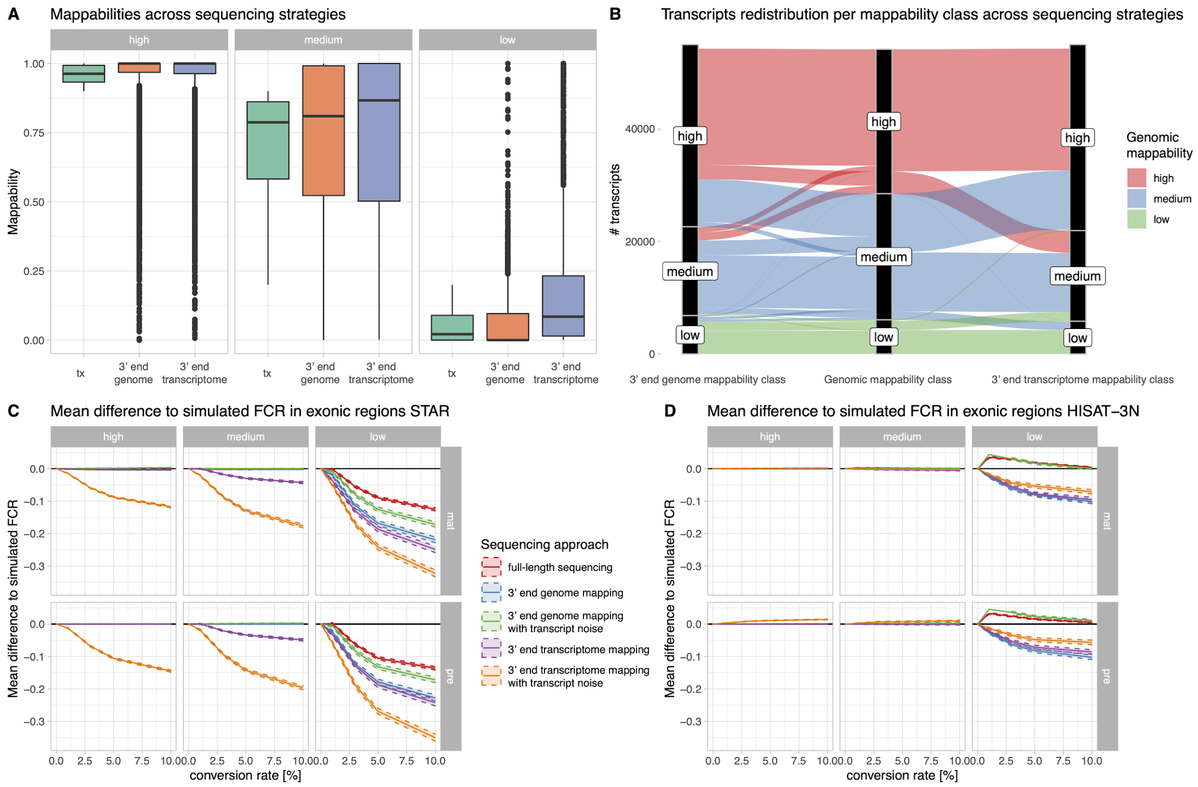

Fig. S8: **The reduced sequence space of 3' end sequencing leads to less robust FCR estimates despite higher mappability. A** Mean genome mappability per genomic annotation (whole transcript, genomic and transcriptomic 3'end intervals), stratified by whole-transcript mappability segment. **B** Comparison of whole-transcript mappability with corresponding 3'end-interval mappability: Full-length transcript mappability class distribution is shown in the centre, the corresponding redistribution of mappability classes when using the genomic 3'end mappability is shown on the left, when using the 3'end transcriptomic mappability is shown on the right. A considerable number of medium mappable transcripts have highly mappable 3'ends while a smaller proportion of high mappable transcripts showed only medium mappable 3'ends. For low mappable genes, there is little exchange happening and overall we hardly observe any extreme swaps from low to high mappability classes and vice versa. **C+D** Mean difference to simulated FCR for all 3'end sequencing approaches with and without transcript noise in exonic regions for STAR and HISAT-3N respectively. Both quantifications for mature and premature transcripts are shown, stratified for mappability and with increasing conversion rates. All calculations based on the **m_big** 3'end sequencing datasets.

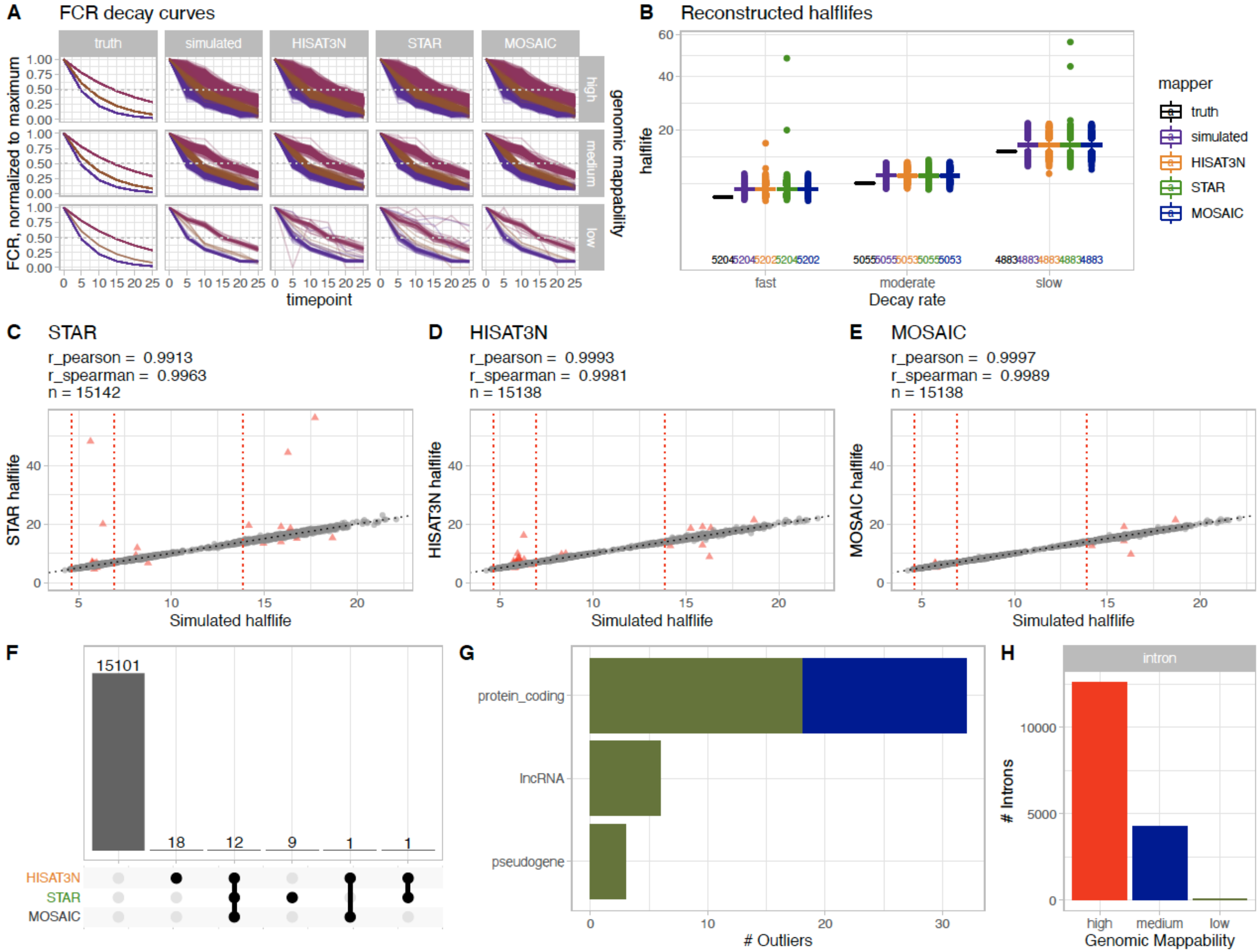

Fig. S9: **Intronic half-life calculations.** Same analysis as in main Fig. 2 but conducted on all 16,995 introns in this dataset of which ~15,100 were recovered. Half-life reconstruction for introns was more accurate than for whole transcripts with only few outliers which can be explained by their higher genomic mappability as shown in panel H**.**

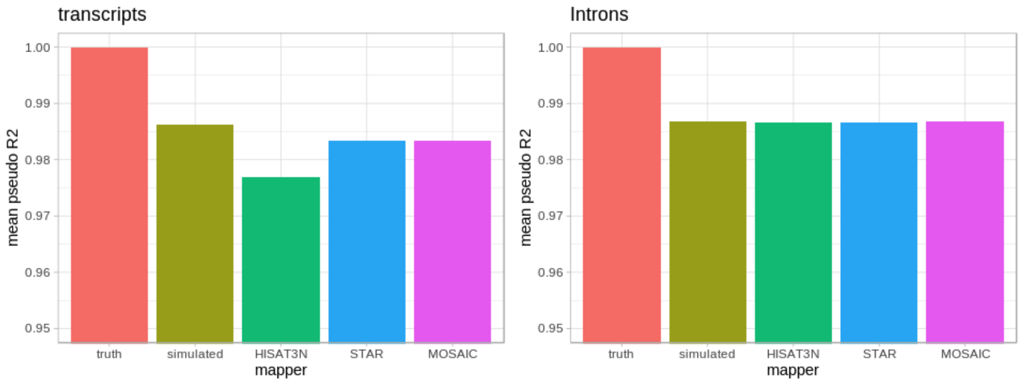

Fig. S10: **Better FCR reconstruction does not necessarily translate into better model fits.** Mean Efron pseudo-R^2 values per mapper, calculated from model residuals after fitting mapper-specific FCR values to a simple exponential decay model (see Methods) as shown in main Fig. 2A (transcripts) and Fig. S9 (introns). Lower values indicate worse model fits.

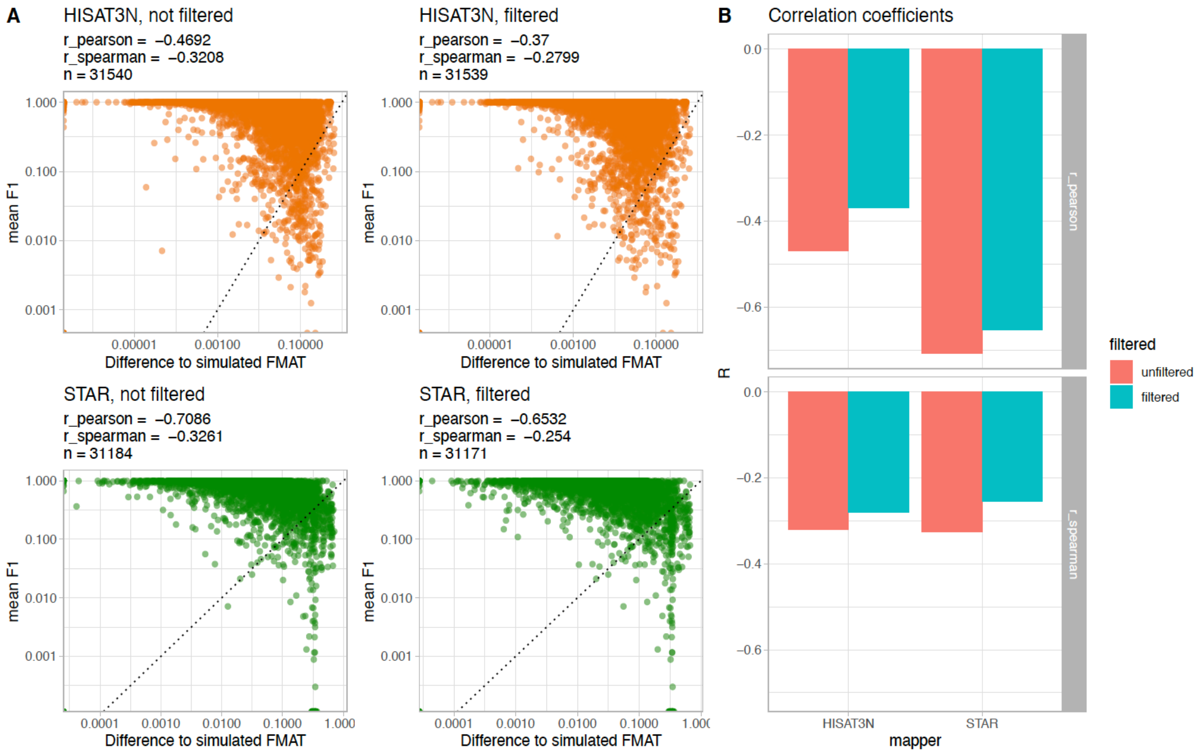

Fig. S11: **Poor FMAT estimates correlate directly with mapping accuracy. A** Negative correlation between mapping accuracy F_1 values and difference between simulated and reconstructed FMAT for unfiltered and intron-filtered data (see main text). **B** Pearson and spearman R values show the negative correlation and its reduction due to intron filtering as F_1 values get closer to the simulated values.

A

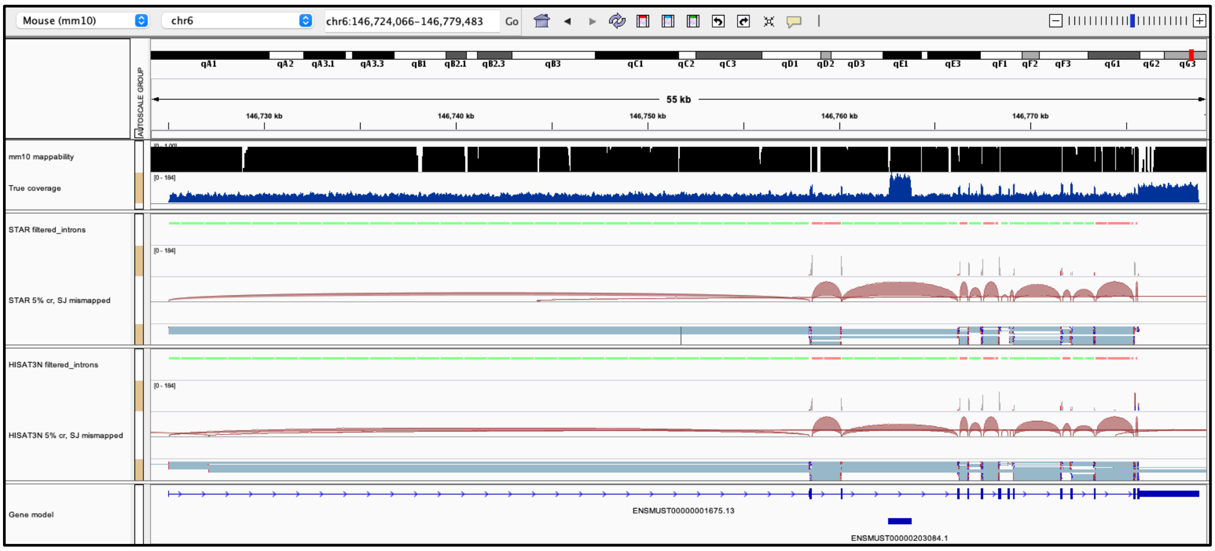

B

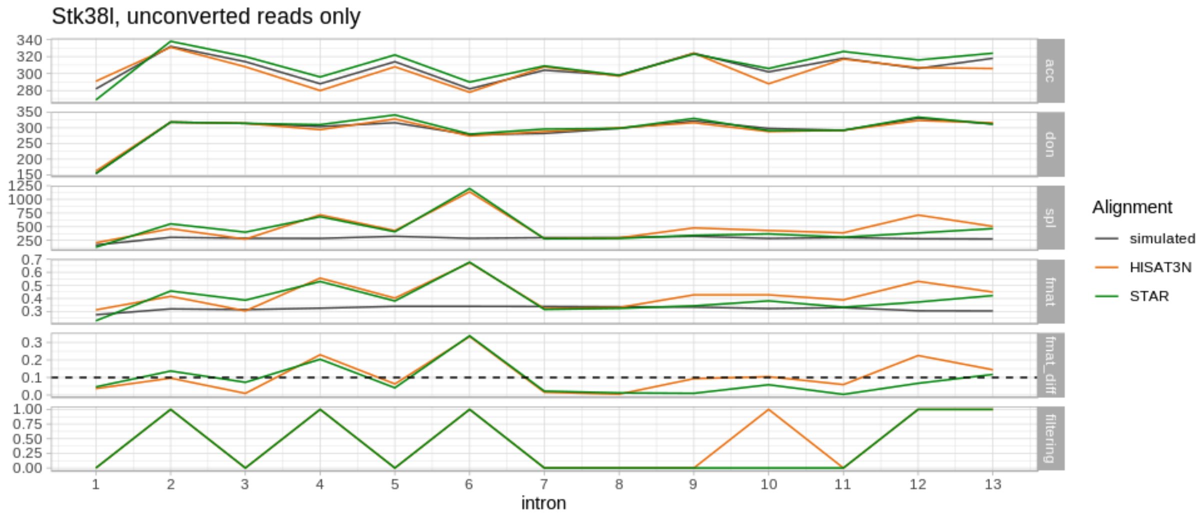

Fig. S12: Exemplary IGV screenshot showcases *splice_sim*'s intron filtering strategy to improve FMAT estimates for *Stk38l* (serine/threonine kinase 38 like). **A** IGV screenshot showing the following tracks (from top to bottom): mm10 genome mappability (black); coverage of the simulated alignment (blue); filter status of introns calculated from the STAR BAM without NC conversions. Filtered introns are depicted in red colour; BAM file with coverage, splice-junction and read track calculated from all mismapped (FN, FP) SJ reads from the STAR alignment with 5% NC conversions. FP reads are coloured red, FN reads light grey; same two tracks for HISAT-3N; gene model showing Stk38l and a hosted (unspliced) gene (Gm44270). **B** Line plot showing selected measures per intron and mapper, calculated from unconverted reads only. From top to bottom: number of acceptor-spanning reads, number of donor-spanning reads, number of spliced reads, simulated/reconstructed FMAT values; difference between simulated and found FMAT. Our cut-off for intron filtering is depicted by the dashed line; filter status per intron (1=filtered) that corresponds to the BED file tracks shown in panel A.

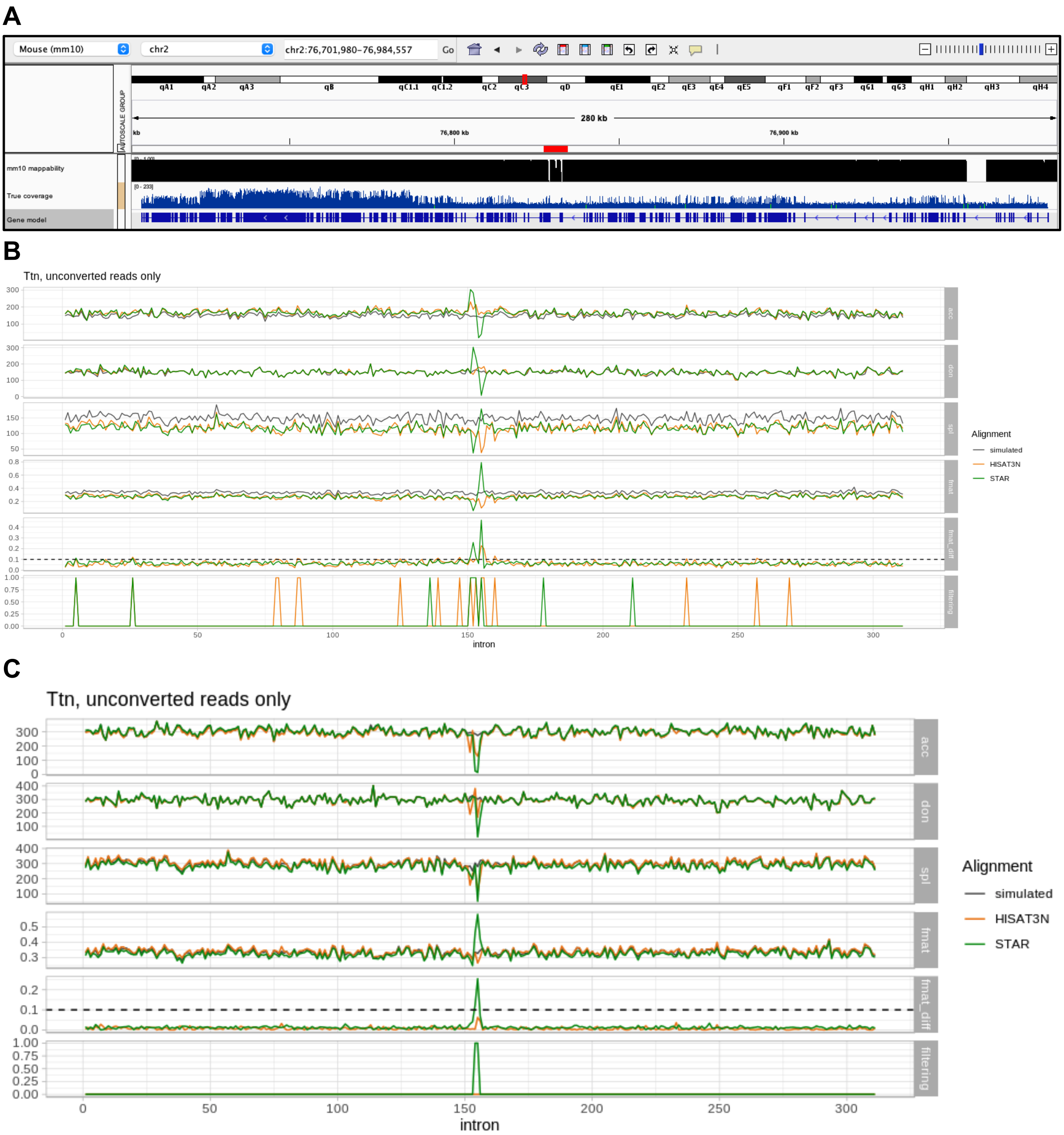

Fig. S13: **Knowledge of splice-junctions is essential to accurately map reads in the long and strongly spliced gene *Titin*.** Exemplary FMAT analysis of *Titin* (Ttn) showing the gene model (**A**) and line plots as in Fig S12 that were calculated from read alignments created without (**B**) and with (**C**) known splice junctions being passed to the respective read mappers. Ttn is a large gene with >300 introns and several alternatively spliced isoforms that play differing roles in skeletal and cardiac muscles. Its genomic region is largely unique, a notable exception being two nearly identical sequence segments right in the middle of the gene that differ only by a single nucleotide insertion and result in low genome mappability (red segment in IGV screenshot **A**). Spliced reads are strongly underestimated in panel **B,** ultimately leading to many filtered introns. Panel **C** shows only small differences to the simulated FMAT except for the mentioned low-mappability regions that is consequently filtered in data derived from STAR alignments.

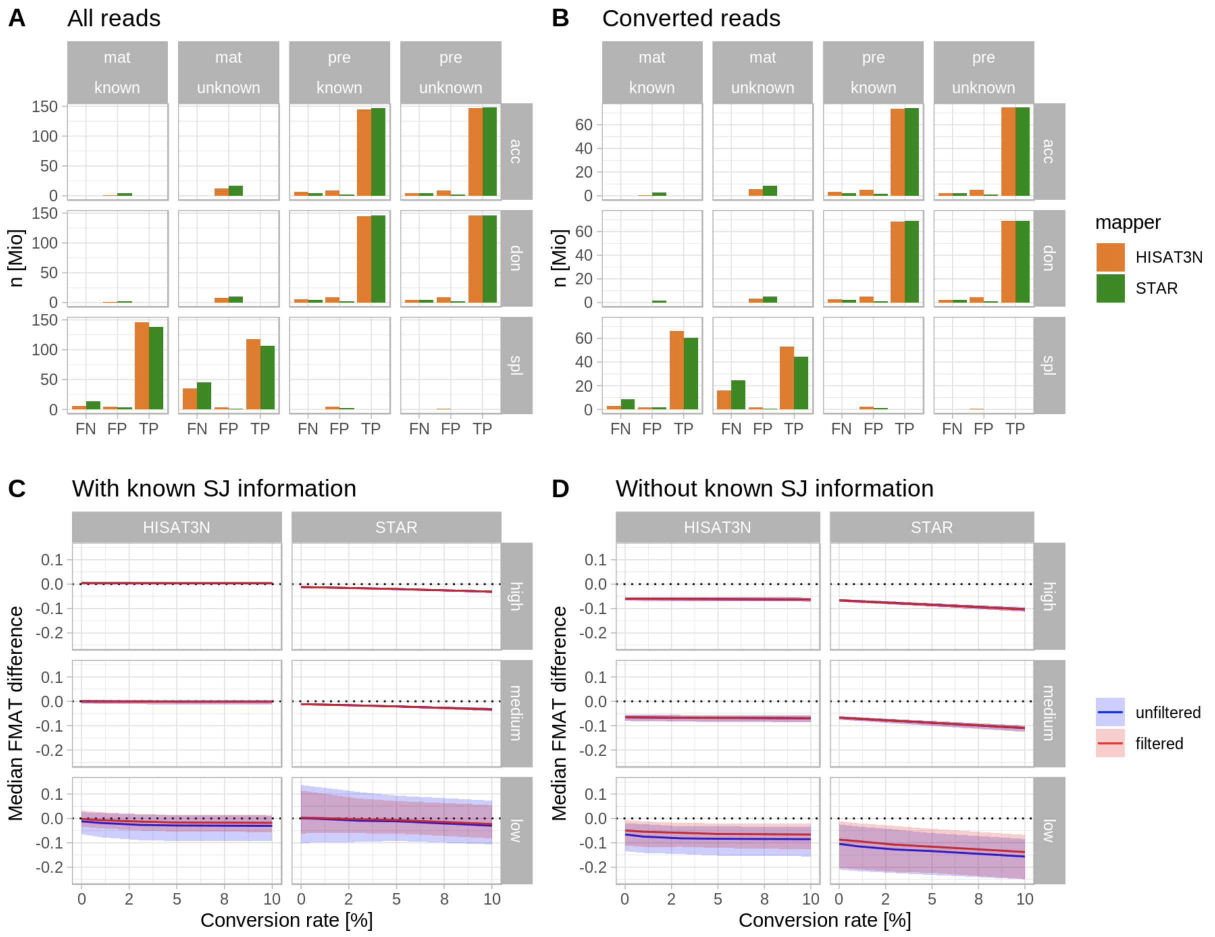

Fig. S14: **Supply of known splice-junctions strongly improves read mapping accuracy and FMAT estimates. A** Numbers of acceptor spanning (*acc*), donor spanning (*don*) and spliced (*spl*) false negative (*FN*), false positive (*FP*) and true positive (*TP*) reads per read mapper, stratified by originating isoform (*mat*: mature, *pre*: premature) and derived from alignments created by either passing (*'known'*) or not passing (*'unknown'*) known SJ coordinates to the respective read mapper. Note that SJ spanning reads stemming from mature isoforms as well as spliced reads stemming from premature isoforms are by definition false positives. Lacking knowledge about true SJs considerably increases the number of false-negative spliced reads and increases false-positive donor/acceptor spanning reads stemming from mature isoforms. Overall, HISAT-3N shows more TP but also maps more FPs stemming from premature isoforms to the acc/don categories. STAR alignments resulted in more FP don/acc reads stemming from mature isoforms. **B** like panel A but considering only reads with at least one NC. **C+D** Median difference to simulated FMAT for unfiltered and intron-filtered data (as in Fig. 3A) with (C) and without (D) known SJ information. Lacking knowledge about true splice junctions leads to a considerable underestimation of true FMAT values for both mappers that can only partially be compensated by intron filtering (see Fig. 3 and discussion in main text).

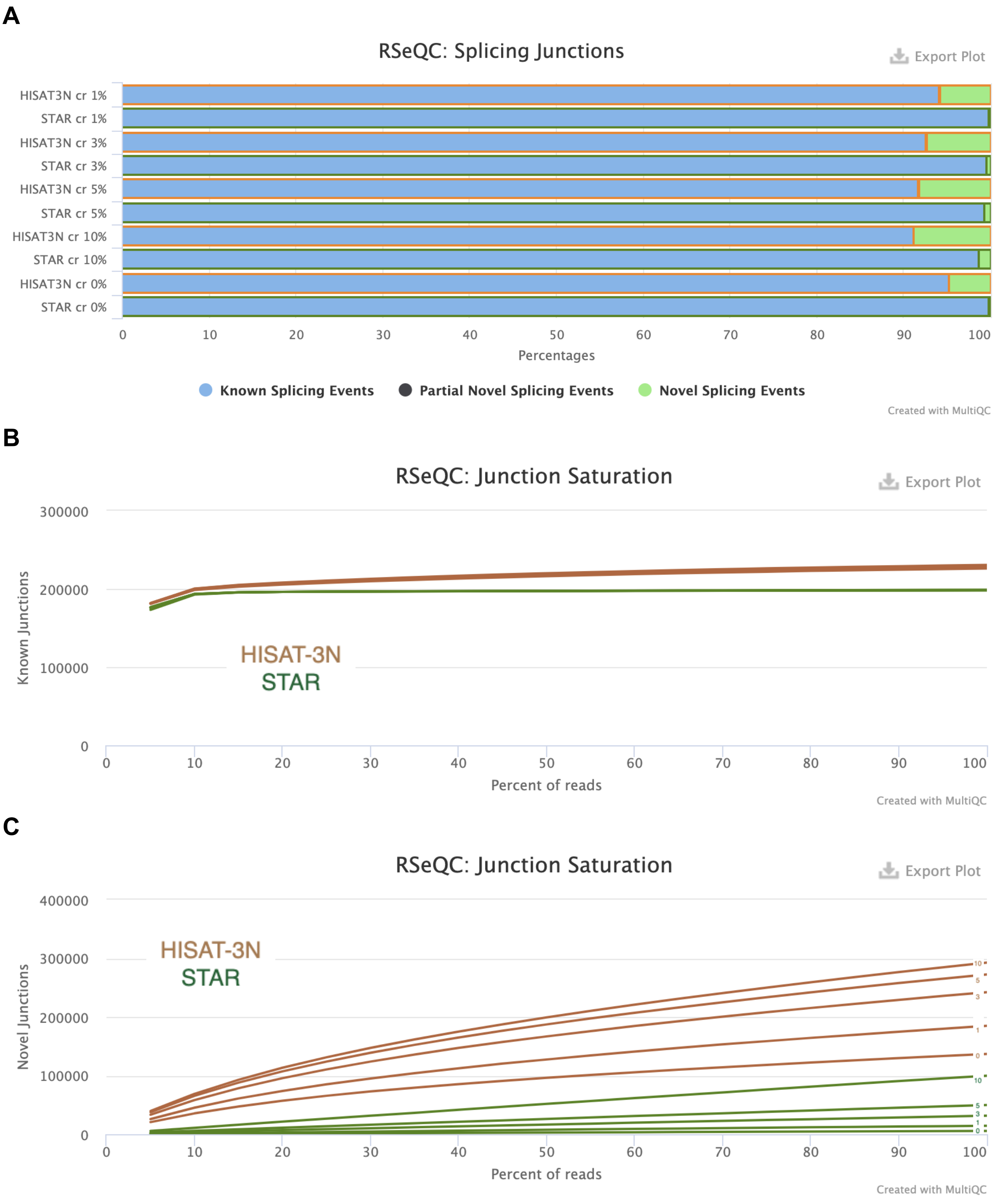

Fig. S15: **HISAT-3N recovers more known splice-junctions but also reports more novel false-positive splice-junctions. A** Junction event percentages as calculated by v2.6.5. [3] **B+C** Known and novel junction counts for HISAT-3N (orange) and STAR (green) as calculated by RSeQC. Note that novel splice junctions are by definition false positives in our dataset. Interestingly, the number of detected novel splice sites increased with the conversion rate (percentage numbers at right end of lines) for both mappers. Data calculated from one **m_big** replicate only.

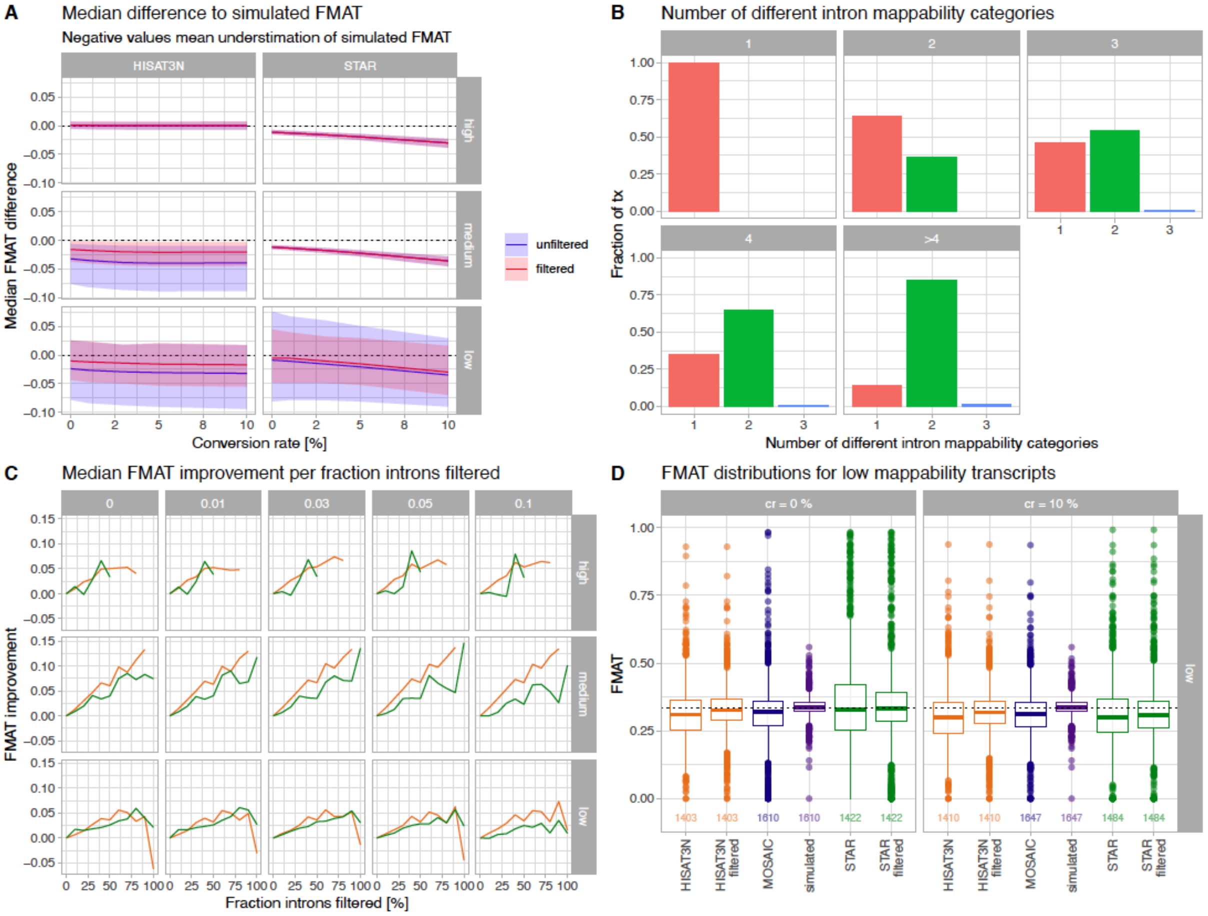

Fig. S16: **Application of FMAT filtering strategies to the human transcriptome leads to comparable FMAT reconstruction improvement.** FMAT reconstruction for ***h_big***, analogous to main Fig. 3.

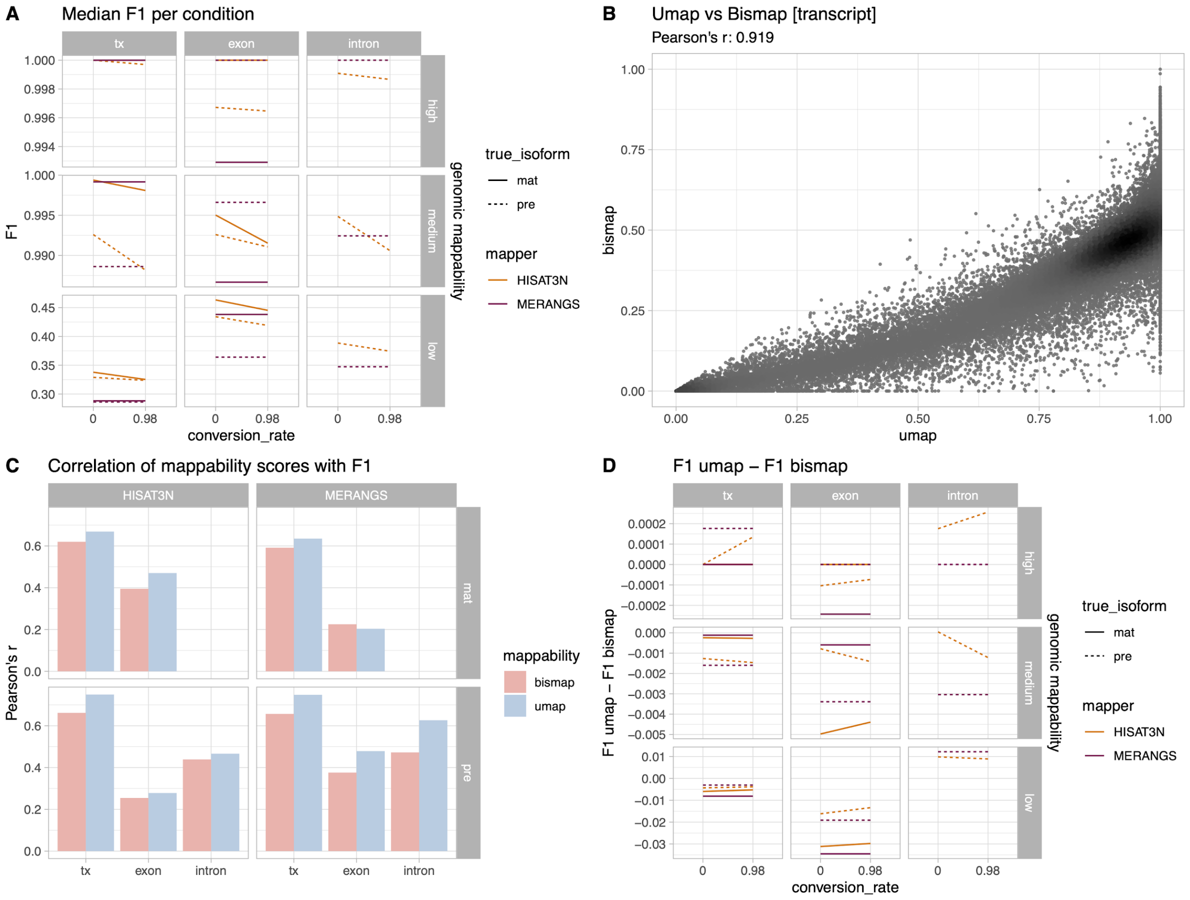

Fig. S17: **The comparable performance of BS-RNA-seq read mapping tools is most influenced by genomic mappability.** NC mapping accuracies for ***m_big_bs*** (mouse BS-RNA-seq data). **A** Mapping accuracy comparison of HISAT-3N and the BS-RNA-seq mapper meRan-Gs in the absence and presence of NC conversions (98% C-to-T conversions). **B** Correlation of umap and bismap mappability scores across all simulated transcripts. **C** Correlation of umap and bismap mappability scores with F_1 performance. **D** Delta F_1 scores between mappability classes stratified using umap mappability scores or bismap mappability scores.

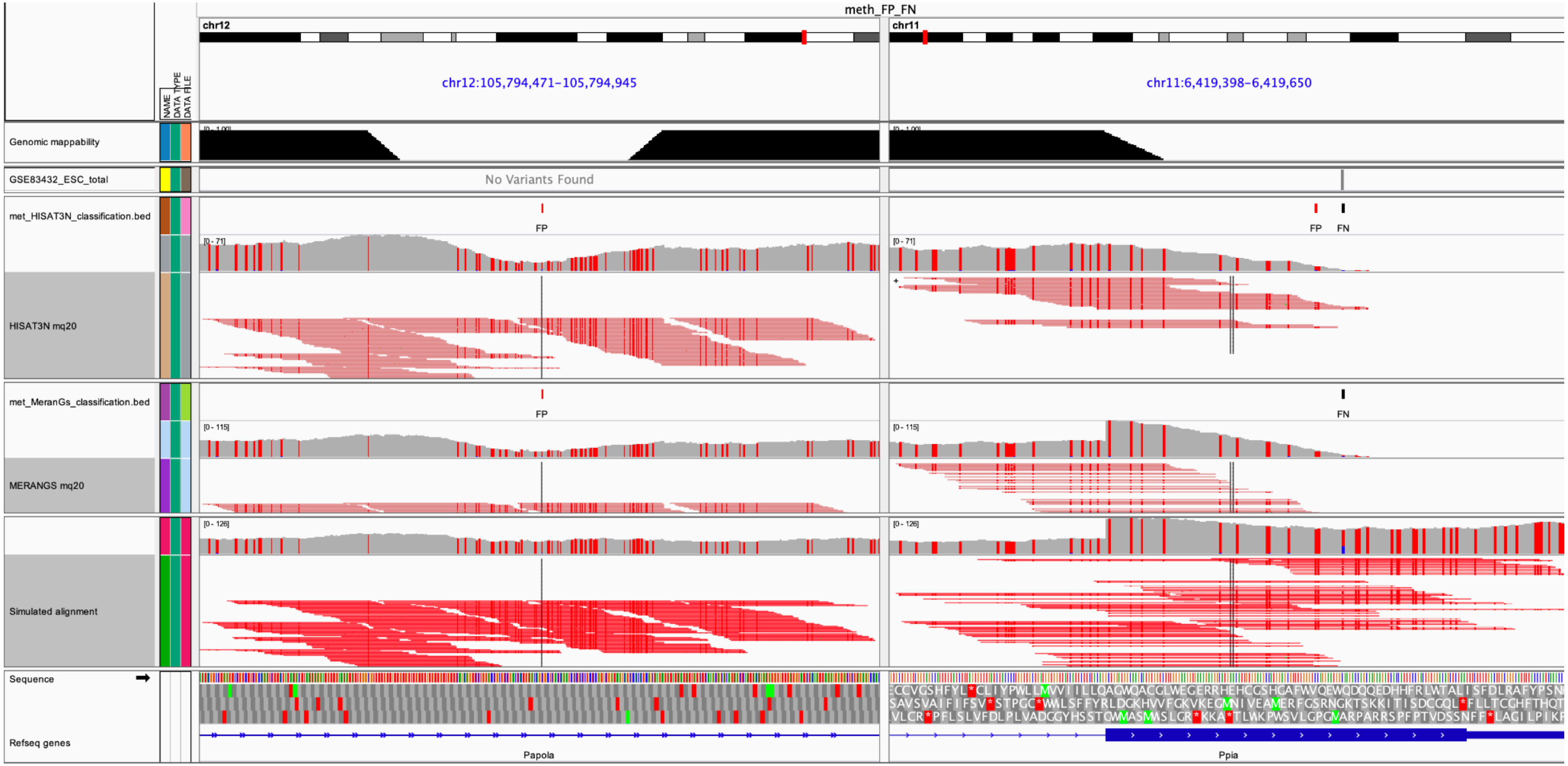

Fig. S18: Exemplary IGV screenshot demonstrating FP and FN methylation calls. Panel left: FP methylation calls. Panel right: FN methylation calls. Tracks (from top to bottom): mm10 genome mappability (black); methylation calls from GSE83432 (truth set); HISAT-3N and MeranGs alignments, filtered for low mapping quality (MAPQ>20). False-positive (red) and false-negative (black) calls are indicated; simulated alignment; gene annotations. FP/FN calls are a result of incomplete bisulfite conversion (conversion rate 98%) and missing (mismapped) reads in low-mappability regions.

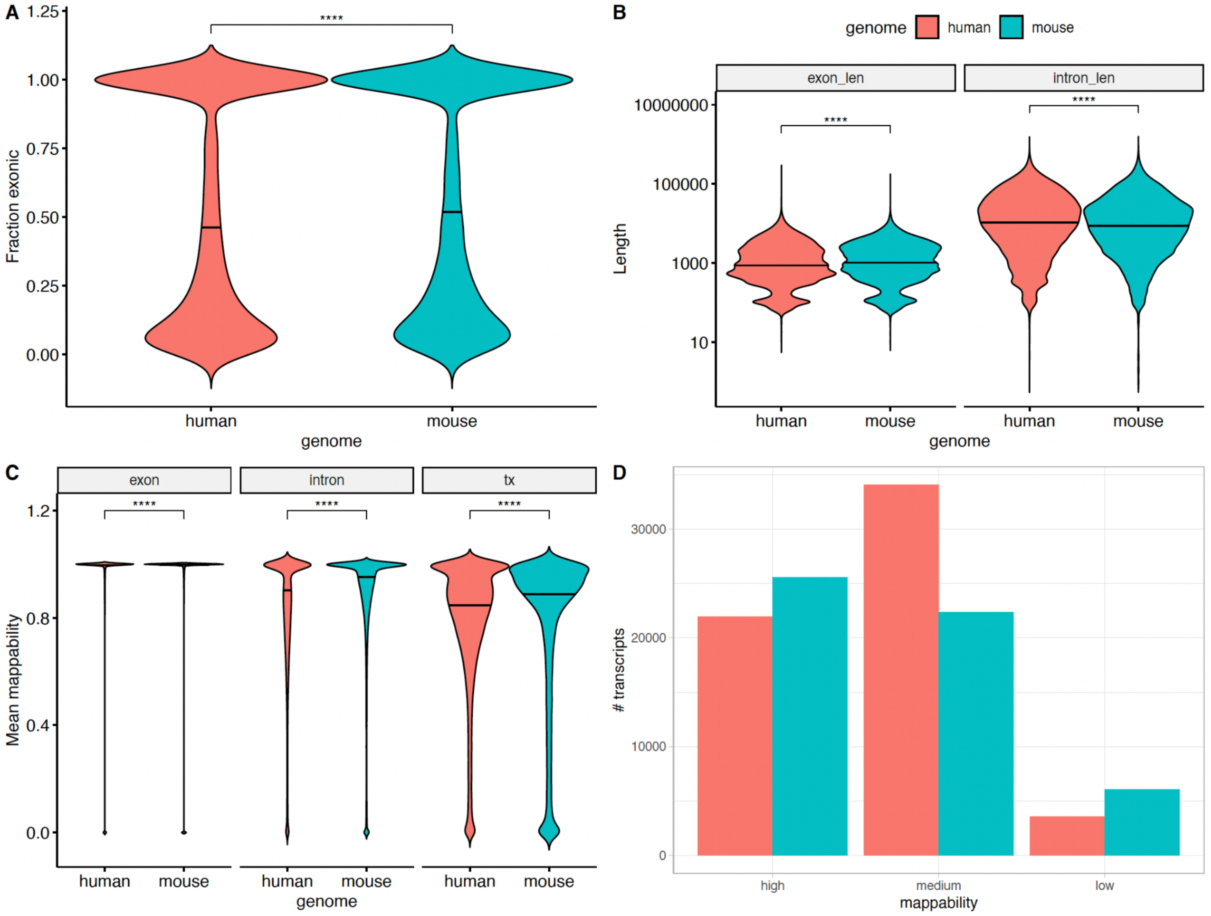

Fig. S19: **The human transcriptome contains a larger fraction of medium mappable transcripts. A** fraction of transcript sequences annotated as exons. **B** length of exons and introns. **C** mean mappability per genomic feature **D** Number of annotations per mappability category. Horizontal lines indicate medians, asterisks indicate significance levels from Mann-Whitney tests (Benjamini-Hochberg adjusted p values; ****=$p_{adj} \leq 0.0001$).

Splice_sim

Splice_sim is a novel, specialised RNA-seq simulation and evaluation framework implemented by Python and R scripts as well as 3rd party tools that are orchestrated by two main nextflow pipelines (simulation and evaluation). Fig. S20 depicts the core components and main result files of these pipelines. To increase reproducibility and usability the complete software stack is bundled in a Docker container.

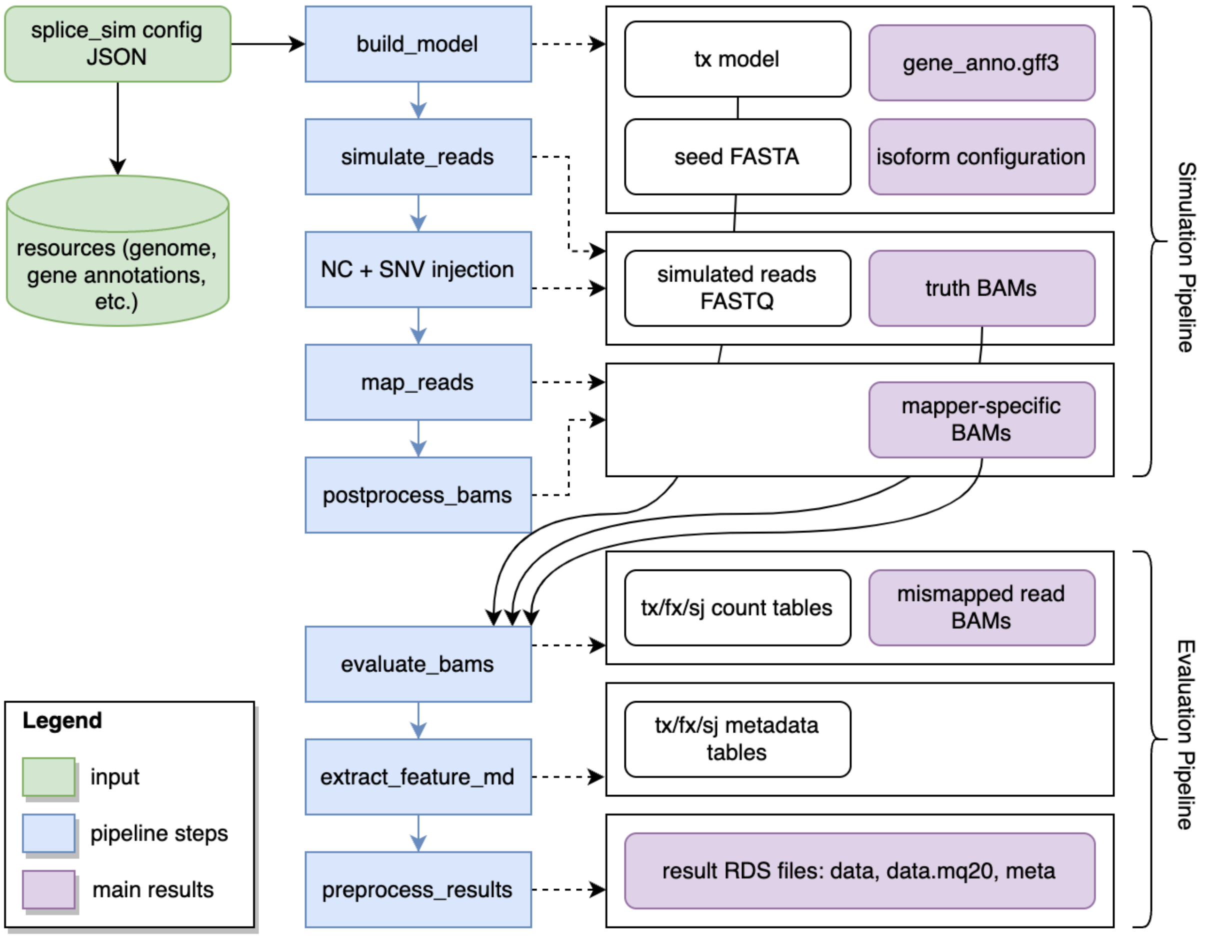

Fig. S20: **Block diagram depicting the core components and result files of *splice_sim*.** Two nextflow workflows (simulation and evaluation) are configured by a single JSON configuration file that links to external resources (e.g., genome sequence and gene annotations) and provides all necessary parameters, such as conversion rates or simulated read length. Briefly, the simulation pipeline builds an isoform model from gene annotations, simulates reads with realistic sequencing errors, calculates genome alignments for those reads and injects nucleotide conversions and single nucleotide variations (SNVs) as configured. Reads are then mapped by the configured read mappers and the resulting alignments are post-processed. The isoform model as well as true and mapped alignments are used by the evaluation pipeline to classify mapped reads as true positive (TP), false positive (FP) and false negative (FN) and to calculate grouped tables with counts per feature of interest (tx: transcripts, fx: exons + introns, sj: splice junctions). Additionally, mismapped (FP/FN) reads are written to BAM files for downstream analyses or visual inspection in a genome browser. Finally, the evaluation pipeline extracts feature-specific metadata from the isoform model and pre-processes all results into R-objects for subsequent analysis and interpretation.

Simulation pipeline

splice_sim is configured with a set of transcript (tx) ids for which a metadata model of exons and introns (isoform model) is built based on a provided gene annotation. Based on the passed configuration (including splicing status per intron), it calculates respective isoform sequences and writes *n* copies (seed sequences) to a FASTA file where *n* is calculated from the respective target coverages and isoform fractions. *Splice_sim* allows configuration of a fraction of these seed FASTA sequences that will not be subjected to nucleotide conversions in subsequent steps and encodes this information in the sequence name.

Isoform mix. In our evaluation, we configured *splice_sim* to either simulate a 1:1 mix between premature (unspliced) and mature (fully spliced) isoforms or, for the decay experiments, to simulate either only mature (decay_sim_nf4) or only premature (decay_sim_nf4_intron) isoforms and vary the fraction of unconverted seed sequences according to an exponential decay model across six timepoints (with arbitrary units). Users may, however, pass a predefined JSON file (e.g., derived from experimental data or bespoke modelling routines) to *splice_sim* that configures each transcript individually. Transcript specific configuration includes abundance and freely configurable isoform mixes where each isoform is defined by splicing status, fraction of total and labelling status. *Splice_sim* automatically adds a mature (fully spliced) isoform in case configured fractions do not add up to one.

Read simulation. Illumina short reads with a configurable read length (100bp in our simulations) are then simulated from this seed FASTA file using ART v2.5.8 and the following command line:

```
art_illumina \
       -ss HS25 \
       -i <seed_fasta> \
       -l 100 \
       -f <coverage> \
       -na --samout \
       -o <dataset_name> \
       -rs <random_seed>
```

As ART simulates reads from both strands, *splice_sim* then reverse-complements reads mapping to the wrong strand and calculates a genome alignment (‘truth BAM’). The following information is encoded in the read names:

- Originating tx id
- Tx strand
- Isoform id: ‘pre’ (premature) or ‘mat’ (mature) in our configuration
- Originating genomic start position
- CIGAR string
- Number of introduced sequencing errors
- Number of introduced nucleotide conversions
- A flag indicating whether the read stems from a converted or unconverted seed sequence

NC simulation. *Splice_sim* then creates converted BAM files from this truth BAM file by randomly introducing the specified nucleotide conversion type into converted read: *Splice_sim*

conducts Bernoulli trials with the respective conversion rate for each convertible base in each read that was simulated from a converted seed sequence. Finally, MD tags are added to all BAM files using samtools [4].

SNV injection. It is possible to pass a VCF file containing SNVs to *splice_sim*. Each SNV may contain an optional probability value in the INFO field (probability is 1 if omitted) that is used to conduct a Bernoulli trial for each read to determine whether the alternate allele will be injected at a particular site. We used this mechanism for our m^5C simulations.

Read mapping. The resulting read sequences/names are then written to FASTQ files (including an unconverted version) that are then used as input by the evaluated read mappers. We mapped to mouse/mm10 and human/GRCh38 genomes respectively with the following read mappers, using default settings unless indicated otherwise:

- STAR v2.7.1 command line:

```
STAR  --readFilesCommand zcat \
      --outFileNamePrefix <base_name> \
      --runThreadN <n_cpus> \
      --genomeDir <star_genome_idx> \
      --readFilesIn <simulated_fastq> \
      --outSAMattributes NH HI AS nM MD \
      --outSAMunmapped Within \
      --sjdbGTFfile <splice_site_gtf>
```

  SAM output files were then converted to BAM format, sorted, and indexed.
  (The splice-site configuration was omitted for the respective analysis as described below)

- HISAT-3N v2.2.1-3n command line:

```
HISAT-3N --base-change <REF>,<ALT> \
         --index <hisat3n_idx> \
         -U <simulated_fastq> \
         --threads <n_cpus> \
         --known-splicesite-infile <hisat3n_kss> \
         -S <base_name>.sam
```

  SAM output files were then converted to BAM format, sorted, and indexed.
  (The splice-site configuration was omitted for the respective analysis as described below)

- meRanTK-1.2.1b with STAR-2.6.1d (as newer STAR versions are not supported):

```
meRanGs align -o . \
        -f <simulated_fq> \
        -t <n_cpus> \
        -S <base_name>.sam \
        -un -ud <unaligned_reads> \
        -id ${params.mappers.MERANGS.merangs_genome_idx} \
        -mbp \
        -starcmd STAR-2.6.1d \
        -star_outSAMattributes NH HI AS nM MD \
        -star_sjdbGTFfile <splice_site_gtf>
```

  Unmapped read FASTQ files were then converted to BAM format and merged with the mapped reads

- Segemehl v0.3.4 command line:

```
segemehl \
       -i <segemehl_genome_idx> \
       -j <segemehl_second_genome_idx> \
       -d <genome_fasta> \
       -q <simulated_fastq> \
       -t <n_cpus> \
       -o <base_name>.sam \
       -F 2
```

  SAM output files were then converted to BAM format, sorted, and indexed.

In a post-processing step, secondary and supplementary alignments are removed from the resulting BAM files and 'YC' tags are added that enable read colouring based on different properties (e.g., originating isoform or read classification) in a genome browser. Read colours can be configured via the central JSON configuration file. Fig. S21 shows an exemplary IGV screenshot of the resulting (coloured) BAM files.

Transcript mappability calculation. As the precomputed *umap* mappability tracks cannot be reasonably subset to transcript sequences, we had to employ alternative methods to calculate transcript 3'end mappability. To this end, we used *genmap* [5] v1.3.0 to calculate mappability scores in a comparable fashion to *umap*.

```
genmap index -F <transcriptome fasta> -I genmap_index

genmap map -K 30 -E 2 -I genmap_index -O . -bg
```

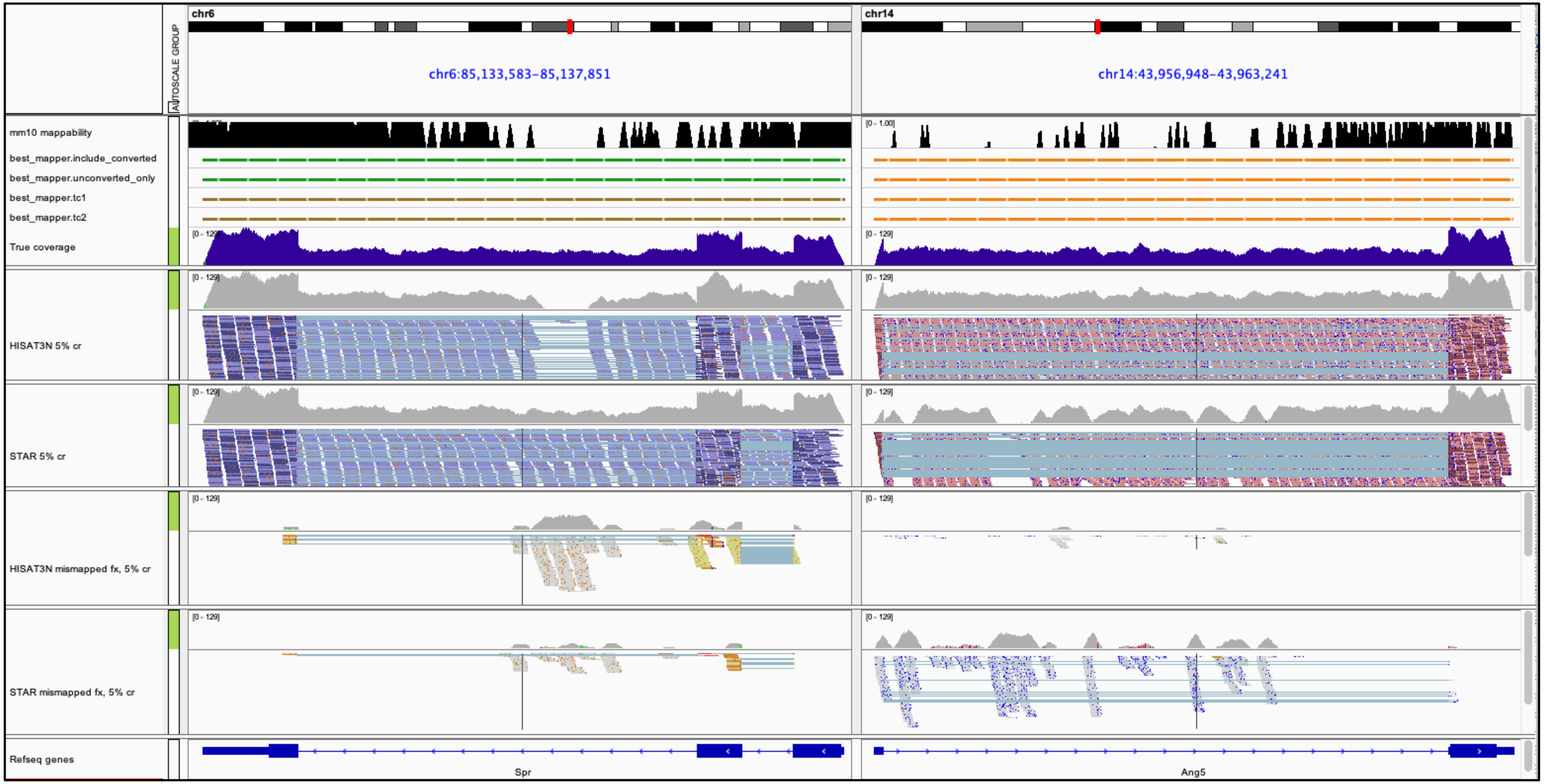

Fig. S21: Exemplified IGV screenshot showcases *splice_sim*'s fine-granular mapper recommendation. IGV screenshot showing two genes for which STAR (left panel, Spr) respectively HISAT-3N (right panel, Ang5) were chosen as best mapper. Tracks (from top to bottom): mm10 genome mappability (black); 4 bed files highlighting the best chosen mapper from our analysis (green: STAR, orange: HISAT-3N, brown: both mappers work reasonable well) based on (1) all reads, (2) unconverted reads only, (3) only converted (tc1: at least 1 NC) or (4) only strongly converted (tc2: at least 2 NC) reads; coverage of the simulated alignment (blue); HISAT-3N alignment; STAR alignment; mismapped HISAT-3N reads; mismapped STAR reads; gene model. The left panel shows a higher number of mismapped false-positive (orange/yellow colour) and false-negative (grey) HISAT-3N reads. The right panel reveals a high number of false-negative reads in the STAR alignment. Alignment files from the first **m_big** replicate with conversion rate 5%. Note that all coverage tracks were group auto-scaled (light-green bars). Also note that, for Spl, the STAR advantage is gone when considering only converted reads (brown tc1/tc2 tracks).

Evaluation pipeline

In a separate second stage, *splice_sim* analyses mapper-specific BAM files and quantifies read counts for the following genomic feature categories:

- Tx: whole transcript counts
- Fx: intronic/exonic counts
- Sj: counts for donor/acceptor overlapping and spliced reads per intron

Splice_sim reconstructs the originating (simulated) read based on information parsed from the read name, compares it to the mapped read and adds counts to the respective (overlapping and/or originating) annotation features:

- True positive (TP): mapped read aligns (at least 1bp) to the true feature(s) / strand(s). For category SJ it is checked whether the read overlaps the splice donor/acceptor site or whether the read splices the true intron (allowing for a maximum difference of 5bp for the overlap between intron and N-block in the read CIGAR string).

- False negative (FN): Mapped read does not satisfy the above-mentioned criteria and is counted as false-negative for the true feature(s).

- False positive (FP): Mapped read overlaps with other features and is counted as false-positive (FP_raw) for every overlapping feature. To avoid an imbalance between TP/TN and FP counts, we then additionally split these counts over all overlapping features, i.e., a read contributes a fraction count 1/n_overlapping_features to each feature.

Counts are grouped by the following categories in the resulting count tables:

- Read mapper
- Conversion rate
- Feature/transcript
- Originating isoform
- Reads with at least 1/at least 2 NCs
- Reads with at least 1/at least 2 simulated sequencing errors

The columns of the resulting count tables are described in Table S1. *Splice_sim* creates two versions of these tables, one considering all reads and a second one considering only reads with a mapping quality>20. Finally, *splice_sim* pre-processes these data tables using an R script and exports the respective data object as RDS files for subsequent analysis in R. Additionally it writes mismapped (FP and FN) reads per feature category (tx, fx and sj) to BAM files ready for visual inspection in a genome browser or downstream processing by other tools.

Column	Description	Notes
mapper	Name of the respective read mapper	
conversion_rate	Conversion rate between 0 and 1.0	
fid	feature/transcript id	
true_isoform	name of the isoform (as configured) this read originates from	
cv1	1 if read contains at least one NC or 0 otherwise	
cv2	1 if read contains at least two NC or 0 otherwise	
se1	1 if read contains at least one simulated sequencing error or 0 otherwise	
se2	1 if read contains at least two simulated sequencing errors or 0 otherwise	
classification	Read classification: TP: true positive, FN: false negative: FP_raw: false-positive/not normalised, FP: false-positive/normalised	
count	read count. FP classified rows may include fractions	
class_type	read type: acc: acceptor spanning, don: donor spanning, spl: spliced read	SJ only
mq_fil	1 if alignments were filtered for mapping quality>20, 0 otherwise	
tid	transcript id	
fid	feature id (intron or exon id)	FX+SJ only
ftype	Feature type: tx, fx,don, acc or spl	
rnk	Rank. For transcripts this is the #exons, for introns/exons it is the rank from the tx 5'-end	
chromosome	Chromosome of the annotation	
start / end	Genomic start/end position of the annotation	
strand	Strand of the annotation	
A/C/T/G	Number of A/C/T/G bases in the annotation sequence	
mean_map	Mean mappability for the annotation. Calculated from the configured mappability bedgraph file.	
tx_rnk	Rank of the transcript	FX+SJ only
num_exons	Number of exons in transcript; 1,2,3,4,5,>5	
tx_mappability	transcript mappability, factor w levels: low, medium, high	FX+SJ only
len	length of annotation	
mappability	annotation mappability, factor w levels: low, medium, high	
GC	Fraction of G/C for annotation	
frac_convertible	Fraction of convertible bases for annotation	
convertibility	Convertibility, factor w levels: low, medium, high	
don_ex_A/C/T/G	Number of A/C/T/G bases in exonic part of donor window (genomic window centred on splice donor site with size: 2xreadlen+1)	SJ only
don_in_A/C/T/G	Number of A/C/T/G bases in intronic part of donor window	SJ only
don_win_map	mean mappability of donor window	SJ only
don_mappability	donor window mappability, factor w levels: low, medium, high	SJ only
don_ex_fc	Fraction of convertible bases in exonic part of donor window	SJ only
don_in_fc	Fraction of convertible bases in intronic part of donor window	SJ only
acc_*	Analogous to the splice donor columns above, but for splice acceptor site	SJ only

Table S1: ***splice_sim* quantitative output description.** Result table columns for count (green rows) and metadata tables (blue rows).

Counting of T-to-C conversions. To count T-to-C conversions per read in mapper specific read alignments (e.g., for FCR estimation in decay experiments) we used a custom python script that added respective BAM tags. Note that genomic positions with >80% T-to-C conversions were ignored (masked) by this script as this would likely be done in a realistic scenario to avoid false-positives due to SNPs.

For the decay experiment FCR calculations we extracted reads containing at least one (observed) T-to-C conversion to new ('labelled') BAM files. We counted reads in complete and labelled BAMs using featureCounts [6] v2.0.1 using the command lines below, loaded the result tables into R and calculated FCR per annotation.

```
featureCounts -s 1 -f -M --fraction -t transcript -g ID -a ${gff} -o tx_counts.tsv ${bams}

featureCounts -s 1 -f -M --fraction -t intron -g ID -a ${gff} -o intron_counts.tsv ${bams}
```

Evaluation Datasets

We simulated several *splice_sim* datasets (Table S2), evaluated the read mappers under investigation and calculated NC mapping accuracy tables for mouse and human data. Our main dataset is ***m_big***, containing >50k mm10 transcripts and we calculated three replicates using different random seeds.

Dataset	Genome	#tx	Type	Replicates	Conversion rates %	Time points	Read mappers	Isoform mix	Description	Approx. depth per replicate
**m_big**	mm10	54,920	metabolic labelling	3	0,1,3,5,10	1	STAR, HISAT-3N	1:1	GENCODE vM21 tx; 1 tx per gene with max support in mESC	100X
**m_small**	mm10	2,291	metabolic labelling	1	0,1,3,5,10	1	STAR, HISAT-3N	1:1	Actively transcribed mESC tx	100X
**m_small_150**	mm10	2,291	metabolic labelling	1	0,1,3,5,10	1	STAR, HISAT-3N	1:1	As **m_small**, but read_len=150bp	100X
**m_decay_tx**	mm10	2,291	metabolic labelling	1	0, 5	6	STAR, HISAT-3N	decay mix, mat	decay time course, tx as in m_small	100X
**m_decay_intron**	mm10	2,291	metabolic labelling	1	0, 5	6	STAR, HISAT-3N	decay mix, pre	decay time course, tx as in m_small	100X
**m3_trans**	mm10	54,920	metabolic labelling	1	0,1,3,5,10	1	STAR, HISAT-3N	1:1	simulate from, map to and evaluate transcript 3’ends only	100X
**m3_trans_txnoise**	mm10	54,920	metabolic labelling	1	0,1,3,5,10	1	STAR, HISAT-3N	1:1	simulate and map to full transcript, evaluate transcript 3’ends only	100X
**m3_genomic**	mm10	54,920	metabolic labelling	1	0,1,3,5,10	1	STAR, HISAT-3N	1:1	simulate from 3’ends, map to genome and evaluate genomic 3’end intervals only	100X
**m3_genomic_txnoise**	mm10	54,920	metabolic labelling	1	0,1,3,5,10	1	STAR, HISAT-3N	1:1	simulate from full transcript, map to genome, and evaluate genomic 3’end intervals only	100X
**h_big**	GRCh38	61,487	metabolic labelling	1	0,1,3,5,10	1	STAR, HISAT-3N	1:1	human dataset; all canonical Ensembl tx	100X
**m_big_bs**	mm10	54,920	RNA-BS-seq	1	0, 98	1	STAR, HISAT-3N, MeranGs	1:1	GENCODE vM21 tx; 1 tx per gene with max support in mESC	100X
**m_small_bs**	mm10	1,910	RNA-BS-seq	1	0, 98	1	HISAT-3N, MeranGs, Segemehl	1:1	GSE83432 overlapping tx	100X

Table S2: Evaluation datasets of this study.

Mouse nucleotide labelling datasets

For our main dataset ***m_big***, we used GENCODE vM21 transcript (tx) annotations and selected one isoform per gene. Isoform selection was performed with the following algorithm:

- We counted spliced reads for each annotated tx in an internal reference mESC Ribo-zero RNA-seq dataset and chose the tx with maximum counts in order to select the isoform that is most expressed in this cell type.
- If there were multiple candidates with equal counts (or, e.g., if the gene was unexpressed), we chose the annotated canonical tx where possible (annotations from the UCSC/knownCanonical table).
- Otherwise, we chose the longest tx.

Overall, the annotated canonical tx was chosen in the majority of cases (>94%) while we made sure that the most expressed transcript in our mESC data was chosen. In our final datasets, we recovered 52,115 of 54,920 tx. The filtered transcripts/isoform annotations were either shorter than our read length or contained introns that were longer than 100,000 bp (which we filtered for performance reasons). For ***m_big***, we simulated three replicates with matching configuration with a total of ~7 billion reads. We also created a smaller version of this dataset (***m_small***) for which we selected a subset (n=2,291) of tx that were expressed with TPM>10 in our mESC reference dataset (data not shown). These tx were also selected for the decay experiments (see below). Correlation plots between the simulated mouse datasets are shown in Figures S22+S23. Fig. S24 shows that false-positive reads that change their labelling status are a minor problem in our datasets (cf. Discussion in main manuscript). Fig. S25 depicts the effect of filtering aligned reads by mapping quality (as calculated by the respective mapper) on mapping accuracy as discussed in the Methods section of the main manuscript. We have also created a second version of this dataset (***m_small_150***) that was used to study the influence of read length on mapping accuracies (Fig. S29).

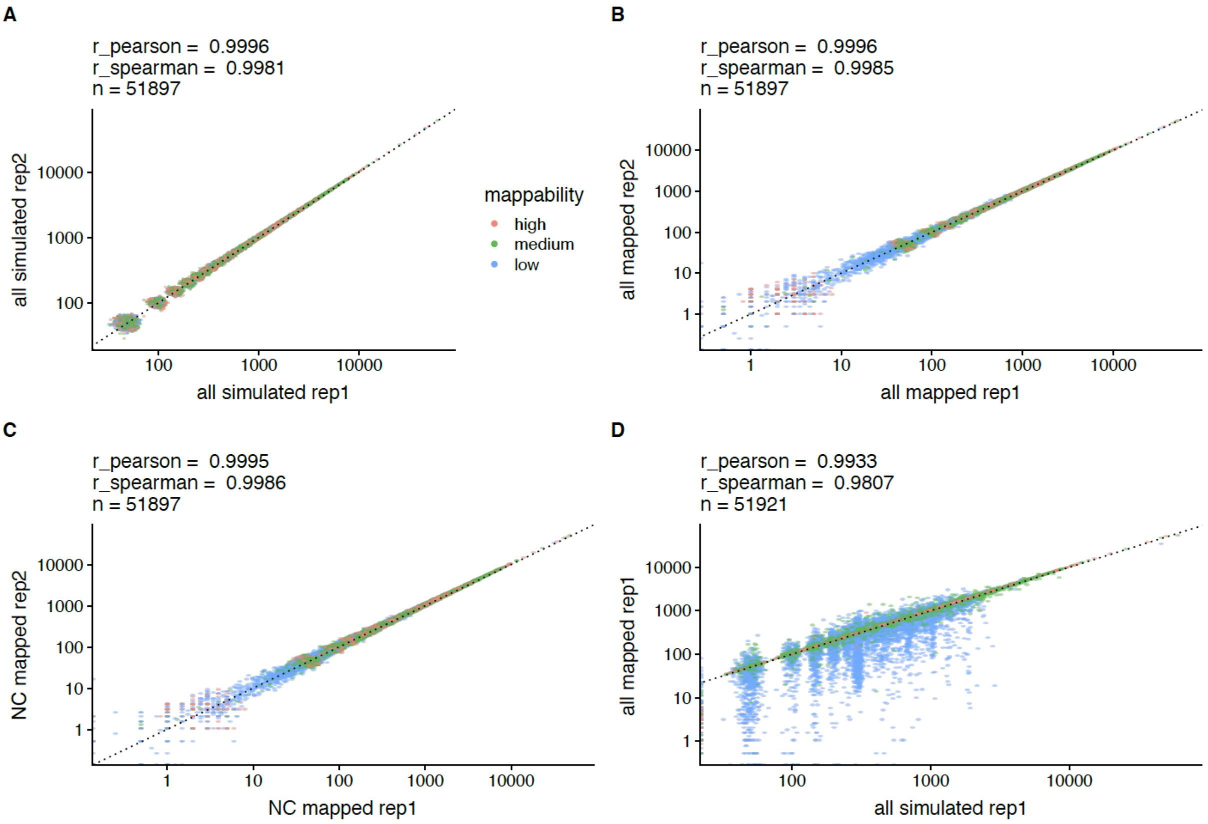

Fig. S22: **Simulation via *splice_sim* does not suffer from stochastic biases across replicates.** Correlation plots of mature transcript read counts from simulated and mapped replicates (mean values of STAR + HISAT-3N counts are plotted) of **m_big** data with 10% conversion rate. Transcripts are coloured by genome mappability. Overall, correlation between replicates and respective mapper specific alignments is very high. **A+B** High correlation between read counts from simulated (A) and mapped (B) alignments for two replicates. **C** Correlation of counts from NC reads (at least one T-to-C conversion) for two replicates. **D** Correlation of read counts from simulated and mapped alignments of the first replicate. Low mappability transcript counts are clearly underestimated.

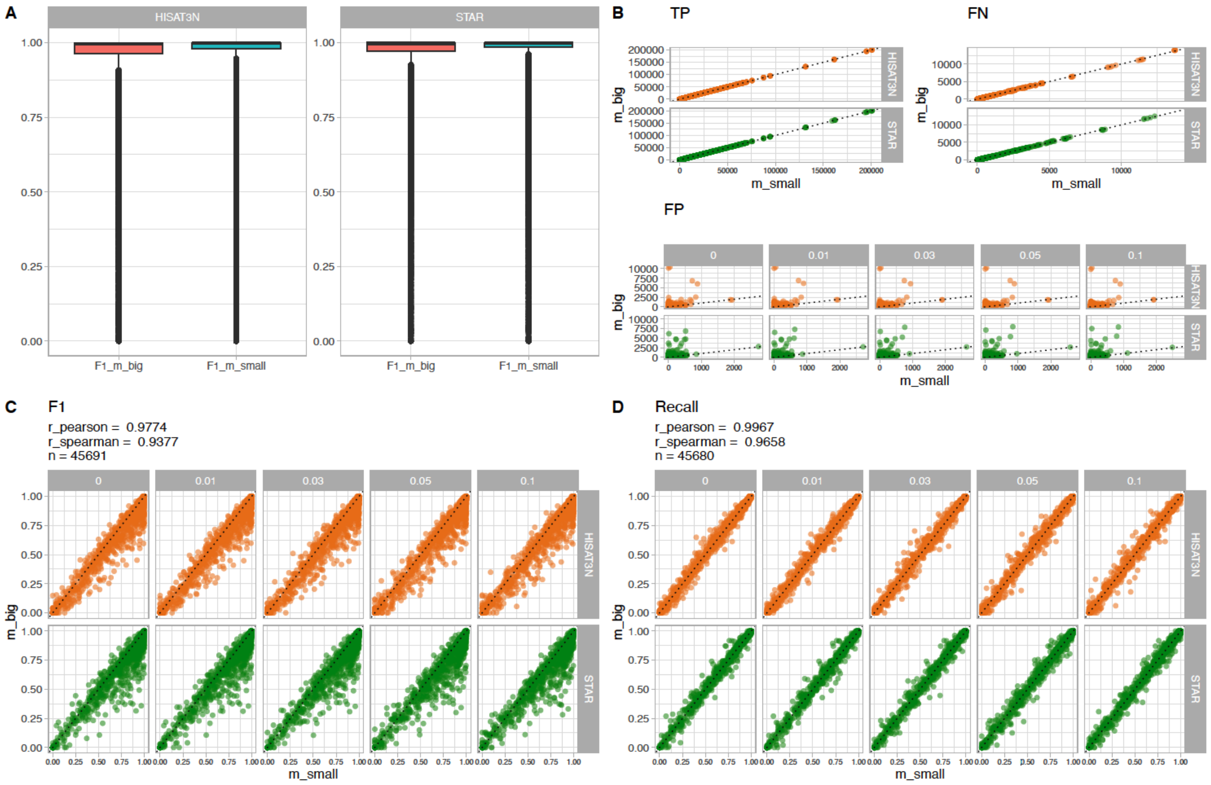

Fig. S23: **Increasing the number of expressed genes inflates FP read mapping while leaving the overall correlation intact.** This figure shows a comparison between our simulation datasets ***m_big*** (>50k mouse genes simulated) and ***m_small*** (~2.3k mESC expressed genes simulated). **A** Boxplots showing ***m_small*** and ***m_big*** F_1 values for all m_small genes. ***M_big*** F_1 values are slightly smaller due to FP inflation as discussed in the main manuscript. **B** Correlation plots between TP, FN and FP (per conversion rate) confirm the FP inflation effect. **C** High F_1 correlation between the datasets despite clearly visible FP inflation effect. **D** Very high recall correlation between datasets. Recall calculation is, by definition, not affected by FPs.

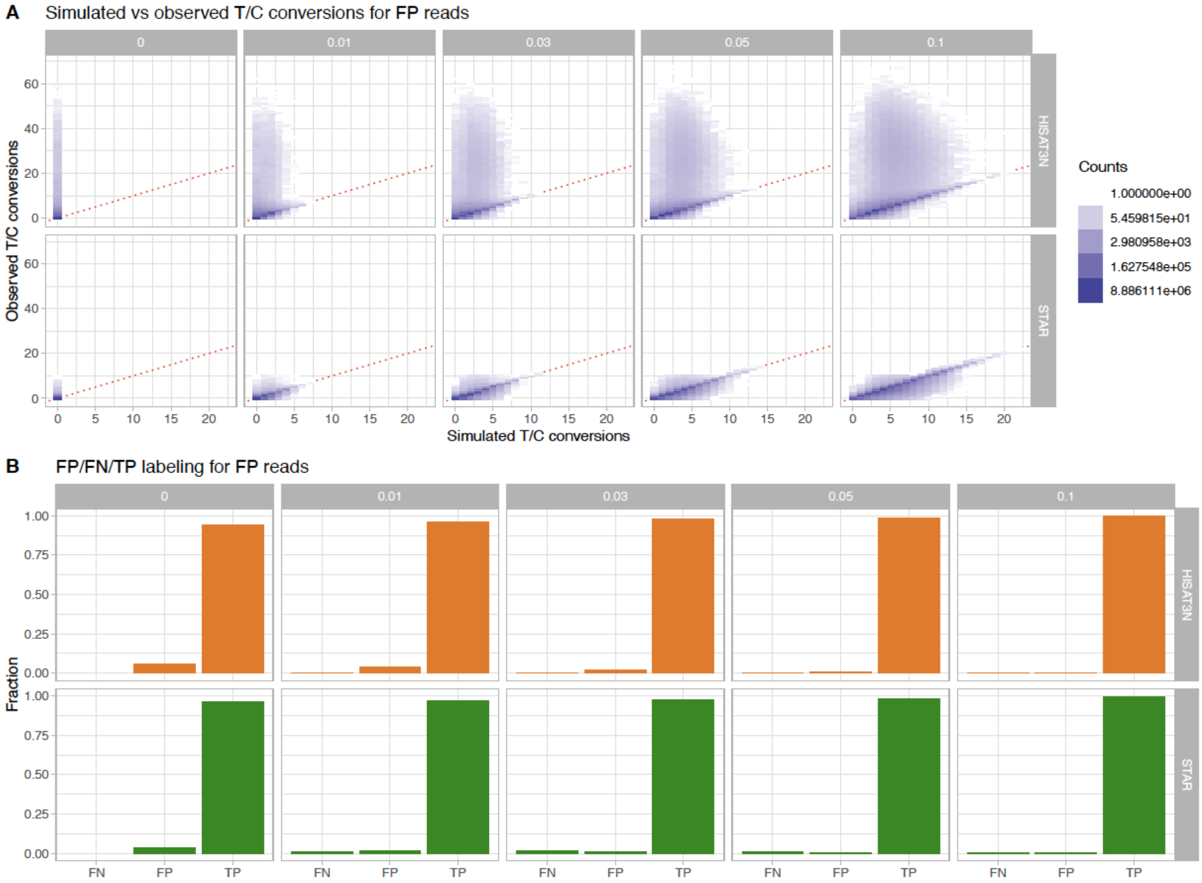

Fig. S24: **Misassignment of reads to the labelled and unlabelled read fraction due to mismapping is a negligible factor in the simulated read set.** Comparison of simulated and detected T-to-C conversions in ***m_big*** false-positive reads *(1st replicate).* Mismapped (false-positive) reads may, depending on read and target sequence, change labelling status from (originally) unlabelled (no T-to-C conversion) to labelled (one or more conversions) and *vice versa*. To assess the dimension of this problem we counted T-to-C conversions in false-positive (mismapped) alignments using a custom python script (see Supplementary methods) and compared them to the simulated values. **A** Simulated *versus* observed T-to-C conversions, red dotted lines indicate equal numbers. Data is stratified by conversion rate and mapper. Note that HISAT-3N produces some alignments with high numbers of conversions as such mismatches are not penalised by the 3N alignment approach. **B** Fractions of false positive reads that were falsely observed to be unlabelled (FN), falsely observed to be labelled (FP) or did not change status (TP). Overall, we observed only few false-positive reads that changed their labelling status.

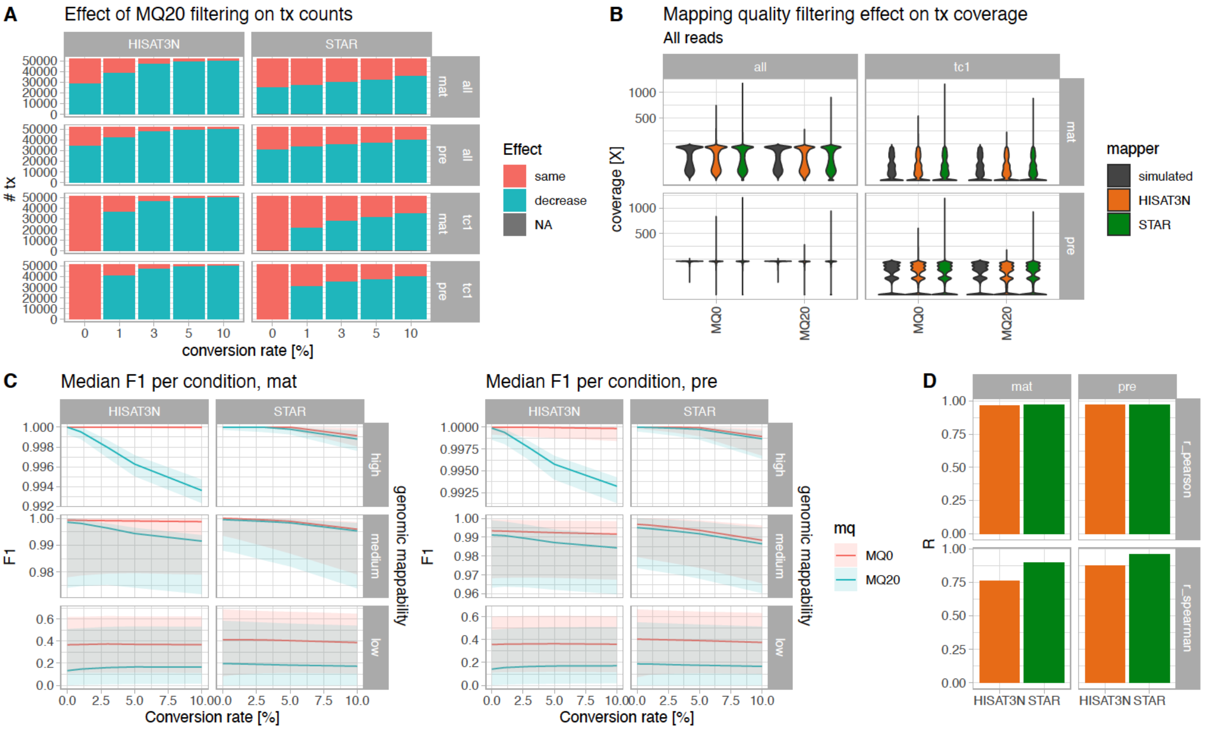

Fig. S25: **Mapping quality filtering decreases mapping accuracies. A** Number of transcripts (tx) with same or decreased counts after mapping quality filtering (MQ>20) per mapper, isoform and NC status (tc1: reads with at least one T-to-C conversion). Note that there are no reads with NC for conversion rate 0 and that the fraction of tx with decreased counts increased with higher conversion rates. **B** MQ20 filtered and unfiltered tx coverage, stratified by NC status and isoform. Overall coverage distributions are very similar. **C** Median tx mapping accuracy (F_1) for MQ20 filtered and unfiltered data stratified by mapper and mappability for mature and premature isoforms (note different y-axis scales when comparing to Fig. 1). IQR is depicted by shaded areas. Overall F_1 is dropping because filtered reads are treated as FN by *splice_sim* and effect is strongest in the low mappability segment as expected. **D** Pearson and Spearman correlation coefficients calculated between MQ20 filtered and unfiltered data.

RNA-BS-seq analysis

For our RNA-BS-seq analysis, we simulated a big dataset **m_big_bs** containing the same transcripts as **m_big** and a conversion rate of 98%. This full dataset contained no methylated positions in order to evaluate mapping performance of BS converted reads only. We then simulated a smaller version (**m_small_bs**) with 1,910 tx that overlap with a set of published methylated m^5C sites called from mESC total polyA RNA-seq data (https://pubmed.ncbi.nlm.nih.gov/28077169, GEO project GSE83432 [7]).

To introduce these methylated sites into our simulated data, we created a VCF file with the respective positions and set the C-to-T conversion rate for these genomic positions according to the published methylation rates (which were called with a 0.2 cutoff by meRanCall). Several m^5C sites could not be simulated as they were (i) located in low coverage regions at transcript ends, (ii) antisense to the overlapping transcript annotations, (iii) in transcripts filtered by *splice_sim* due to too-long introns or (iv) in intergenic regions (cf. main Fig. 4E). We did, however, recover 4,831/7,541 (64%) m^5C sites after applying meRanCall using the same parameters as in the originating study. The mapper alignments (HISAT-3N, MeranGs and Segemehl) were then filtered to remove reads with low mapping quality (MAPQ>20), m^5C sites were called with meRanCall and finally compared to the calls from the simulated alignments.

Decay simulations

We simulated two nucleotide labelling pulse-chase datasets (selecting the same transcripts as for ***m_small***) for evaluating the effects of NC mappability on the reconstruction of decay half-lives for mature transcripts (***m_decay_tx***) and introns (***m_decay_intron***). In such an experiment, cells are typically exposed to 4-thiouridine (4sU) for a considerable time span (e.g., 12h) to ensure a fully labelled RNA population after which 4sU is washed out and RNA is extracted, exposed to nucleotide conversion chemistry followed by cDNA library preparation, and sequenced at multiple consecutive timepoints. The ratio between labelled and unlabelled RNA molecules (i.e., RNA transcribed after the washing time point) is interpreted as a quantification of RNA stability.

Each of our two chase datasets consists of simulation data for six timepoints for which we simulated reads for 2,291 tx with a T-to-C conversion rate of 5%. The fraction of labelled vs unlabelled RNA was configured in a way that the resulting FCR follows an exponential decay curve (cf. main Fig. 2A). For ***m_decay_tx*** we simulated only mature isoforms, for ***m_decay_intron*** only premature ones.

After simulation and read mapping, we calculated the FCR for transcript (***m_decay_tx***) and intron annotations (***m_decay_intron***) per time point (see Evaluation section for a detailed description of this process), fitted the decay model to these data and reconstructed half-lives. Finally, these reconstructed half-life values were compared to the simulated and configured data.

Note that half-life values reconstructed from simulated data are systematically higher than the configured (theoretical) values. This is because for this analysis we considered only reads with at least one NC as being converted, ignoring a considerable share of reads stemming from the converted fraction that have zero NC by chance (i.e., none of the Bernoulli trials conducted for these reads returned a ‘success’ result). Although we could have resorted to the true read origin in our simulated data, we decided to treat simulated and mapped data the same way to keep them comparable and to simulate a realistic analysis scenario in which the origin of reads (from converted or unconverted RNA molecules) is unknown.

3’end sequencing analysis

We simulated four nucleotide labelling experiments, as depicted in Fig. S7 and described in Table S2, using the same set of transcripts and the same conversion rate progression as in our ***m_big*** dataset to evaluate the impact of choosing a 3’end sequencing approach over full-length transcript sequencing. Briefly, 3’end datasets were simulated with two different ‘noise’ levels: (1) an ideal (noise-free) scenario without internal priming events on internal A-stretches (cf. main manuscript) where truly only the 3’ ends of transcripts are amplified and (2) a worst-case scenario where off-target priming is happening along the entire transcript and reads stem from all locations of the transcript (‘transcript noise’). We then mapped the simulated reads to (i) the genome (data with and without transcript noise), (ii) to the transcriptome (data with transcript noise) or (iii) to the 3’end sequences only (data without transcript noise). Finally, we calculated count tables by evaluating the 3’ends in all four scenarios and calculated mean mappability scores for the respective intervals (*umap* for genomic intervals, *genmap* for transcript intervals). As only few of the evaluated 200nt 3’ends span over an exon/intron boundary we abstained from calculating FMATs but focused this comparison on mapping performance and FCR estimation only.

Human nucleotide labelling dataset

For ***h_big***, we used GENCODE v39 transcript annotations and selected the canonical isoform of each gene indicated by the Ensembl canonical annotation tag. In our final datasets, we recovered 57,036 of 61,487 transcripts (92.7%). The filtered transcripts/isoform annotations were either shorter than our read length (100nt) or contained introns that were longer than 100,000 bp (which we filtered for performance reasons). We furthermore filtered 45 transcript ids from the pseudoautosomal regions (PAR) for which there were duplicated annotations in the GENCODE annotation file. Note that the mappability distribution is slightly different for the human genome with more transcripts in the medium mappability category (Fig. S19).

Results files

Based on our deep mouse and human datasets we have compiled tables of the analysed transcripts, their mapping accuracies, derived measures as analysed in this manuscript and the best evaluated read mapper according to the algorithm discussed in the Methods section. The following plot shows a summary of these results for **m_big**.

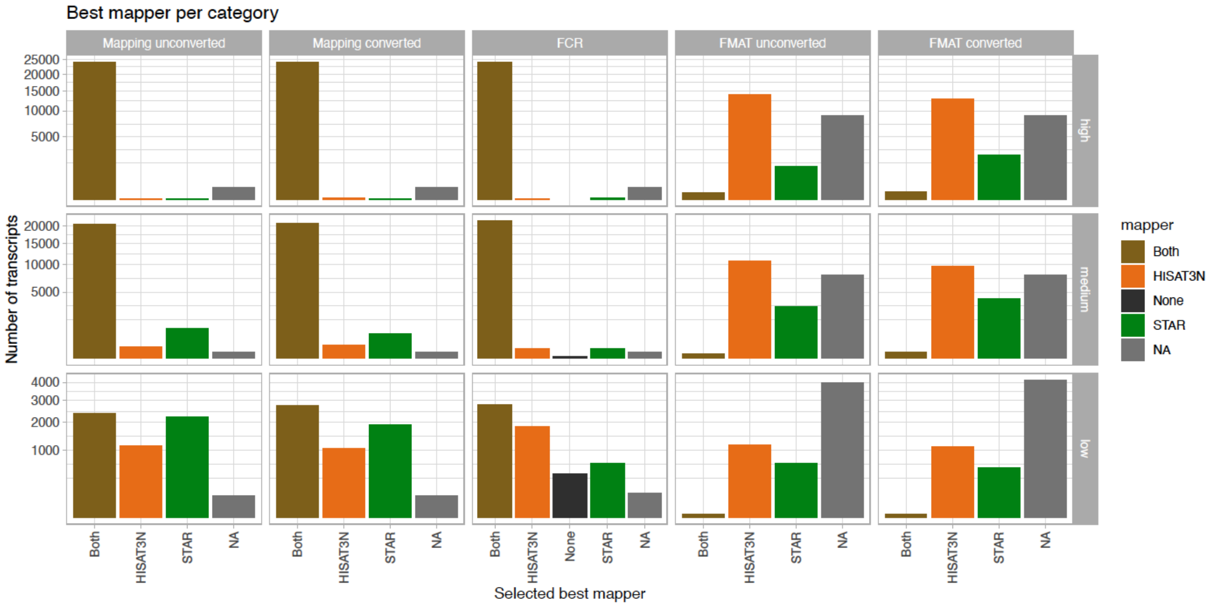

Fig. S26: **Mapping strategy selection guidelines for the mouse transcriptome.** Best evaluated mapper for various categories, stratified by genomic mappability. ***Mapping unconverted***: A mapper was selected if its derived F_1 score was 0.05 higher than the F_1 score from the other mapper. F_1 scores were calculated from unconverted reads (conversion rate 0) only. ***Mapping converted***: same as before but taking data from all conversion rates into account. ***FCR***: A mapper was selected when the calculated difference to the simulated FCR was small (<0.1) and smaller than for the other mapper. If both mappers, however, resulted in values close (<0.05) to the simulated data, both mappers were considered. ***FMAT unconverted***: A mapper was selected if the absolute difference between FMAT values calculated from simulated and mapped data is smaller than for the other mapper. In case of equal FMAT values, both mappers were chosen. If intron filtering improved a mapper's performance, then it was applied. Only unconverted reads (conversion rate 0) were considered. ***FMAT converted***: Same as before but calculated over all conversion rates. More details on the criteria for selecting the best mapper per category are provided in the main methods section.

Resource benchmarks

The following plots were extracted from the Nextflow execution reports (https://www.nextflow.io/docs/latest/tracing.html#execution-report) for the mouse nucleotide labelling and RNA-BS-seq datasets ***m_small***, ***m_big***, ***m_small_bs*** and ***m_big_bs*** to give a realistic estimate about the required computational resources and expected runtimes of our pipeline. Resource configurations to match these requirements are deployed with *splice_sim*.

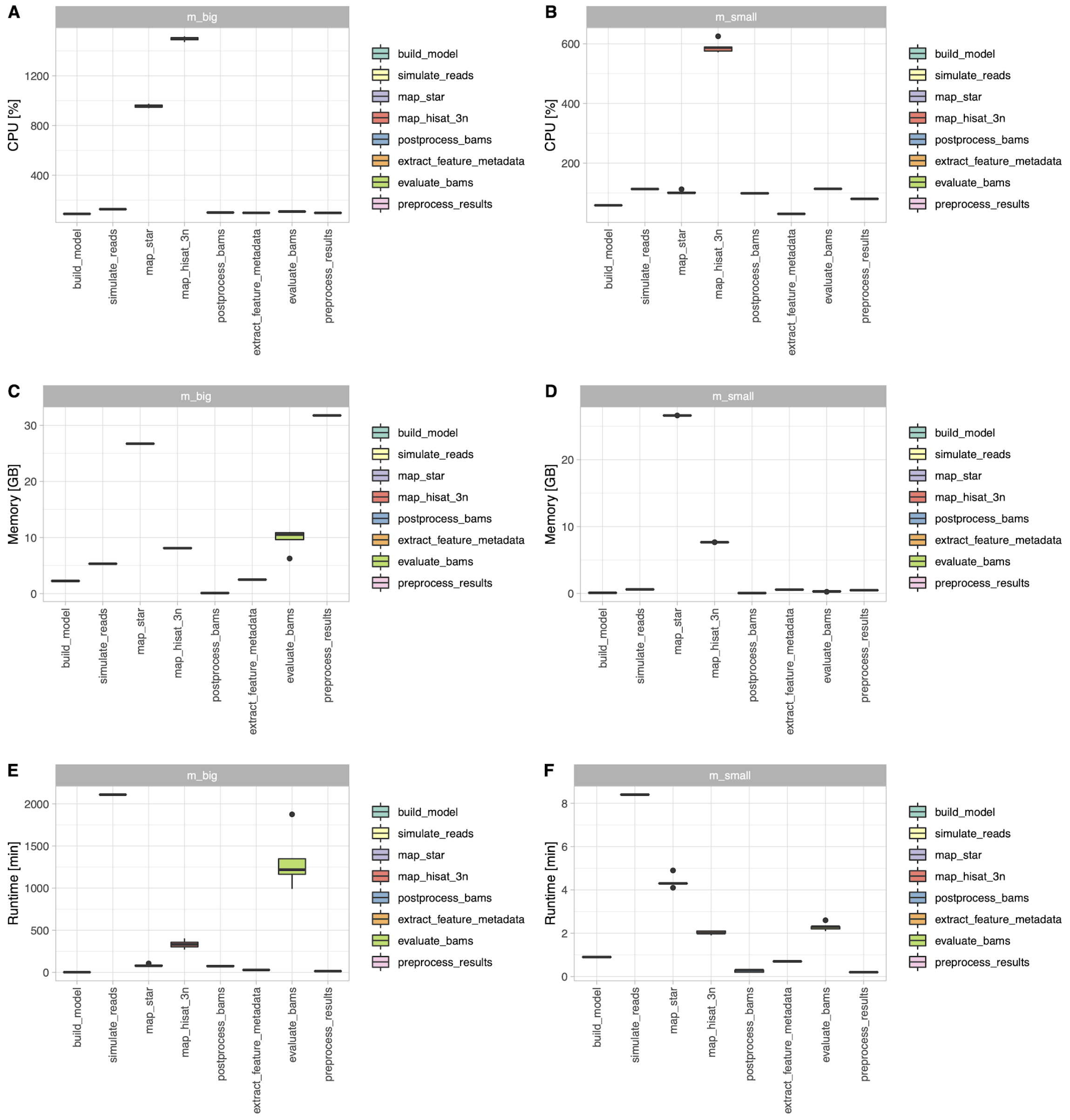

Fig. S27: Resource requirements for the mouse nucleotide labelling datasets. CPU resource consumption (%) for all *splice_sim* processes for ***m_big*** (**A**) and ***m_small*** (**B**); memory resources consumption (GB) for all *splice_sim* processes for ***m_big*** (**C**) and ***m_small*** (**D**); runtime duration (min) for all *splice_sim* processes for ***m_big*** (**E**) and ***m_small*** (**F**).

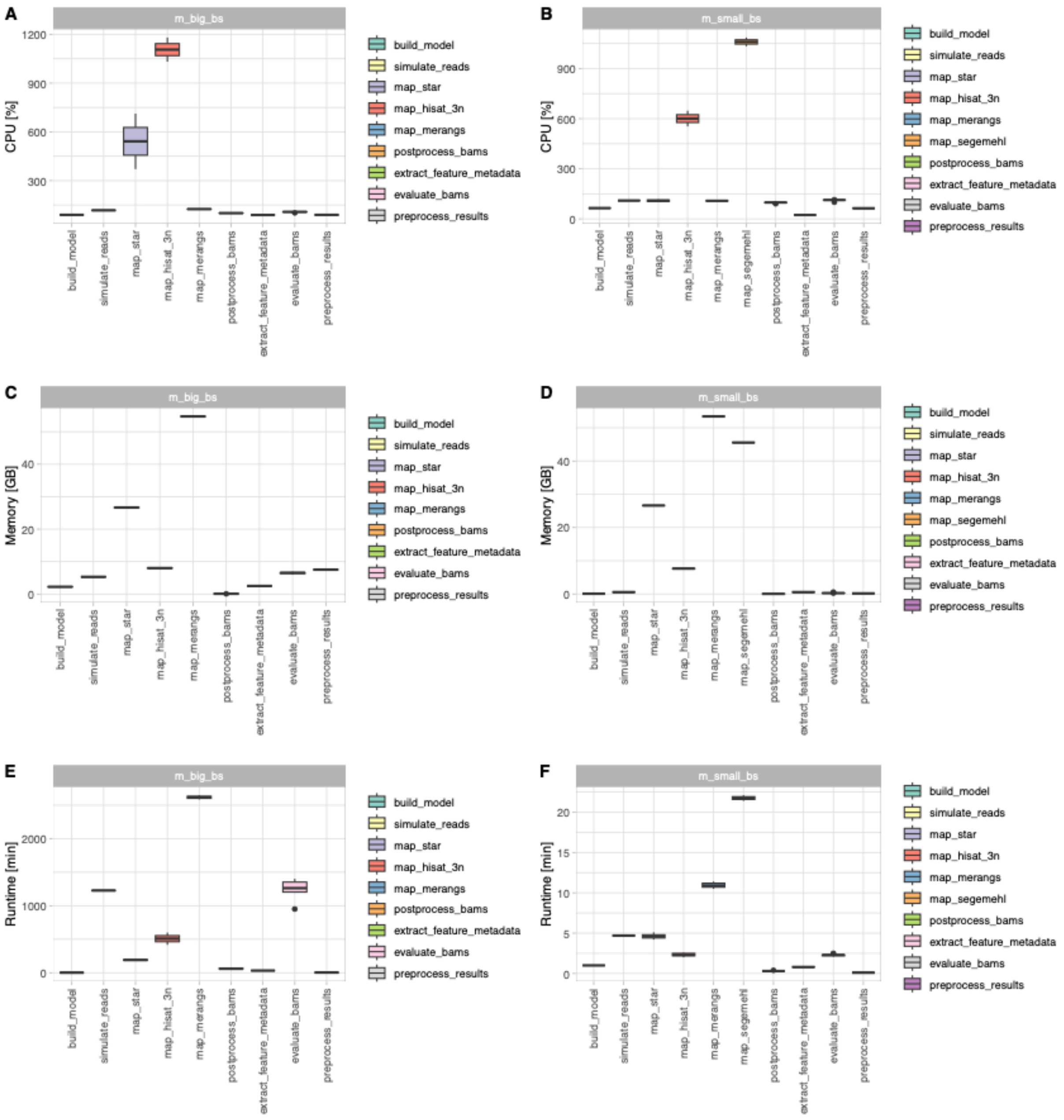

Fig. S28: **Resource requirements for the mouse RNA-BS-seq datasets.** CPU resource consumption (%) for all *splice_sim* processes for ***m_big_bs*** (**A**) and ***m_small_bs*** (**B**); memory resources consumption (GB) for all *splice_sim* processes for ***m_big_bs*** (**C**) and ***m_small_bs*** (**D**); runtime duration (min) for all *splice_sim* processes for ***m_big_bs*** (**E**) and ***m_small_bs*** (**F**).

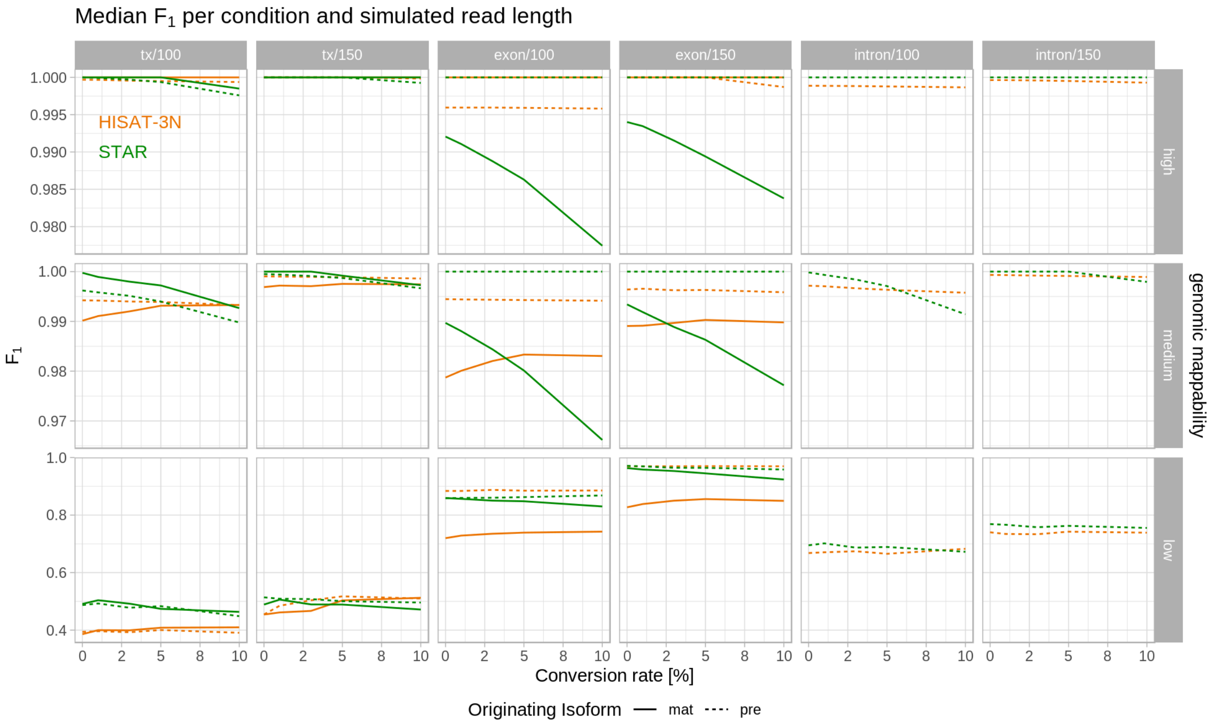

Fig. S29: Influence of read length on mapping accuracy. Median F1 measure per mapper and originating isoform (pre: premature, mat: mature) for different genomic annotations (tx: whole transcript), stratified by mappability and read length, analogous to main Fig. 1D. Here, we compare data from our small mouse SLAM-seq dataset ***m_small*** (read length=100bp) and a corresponding dataset with same parameters but longer reads (***m_small_150***, read length=150bp). Longer reads increase mappability along all categories but show the same characteristics as their shorter counterparts.

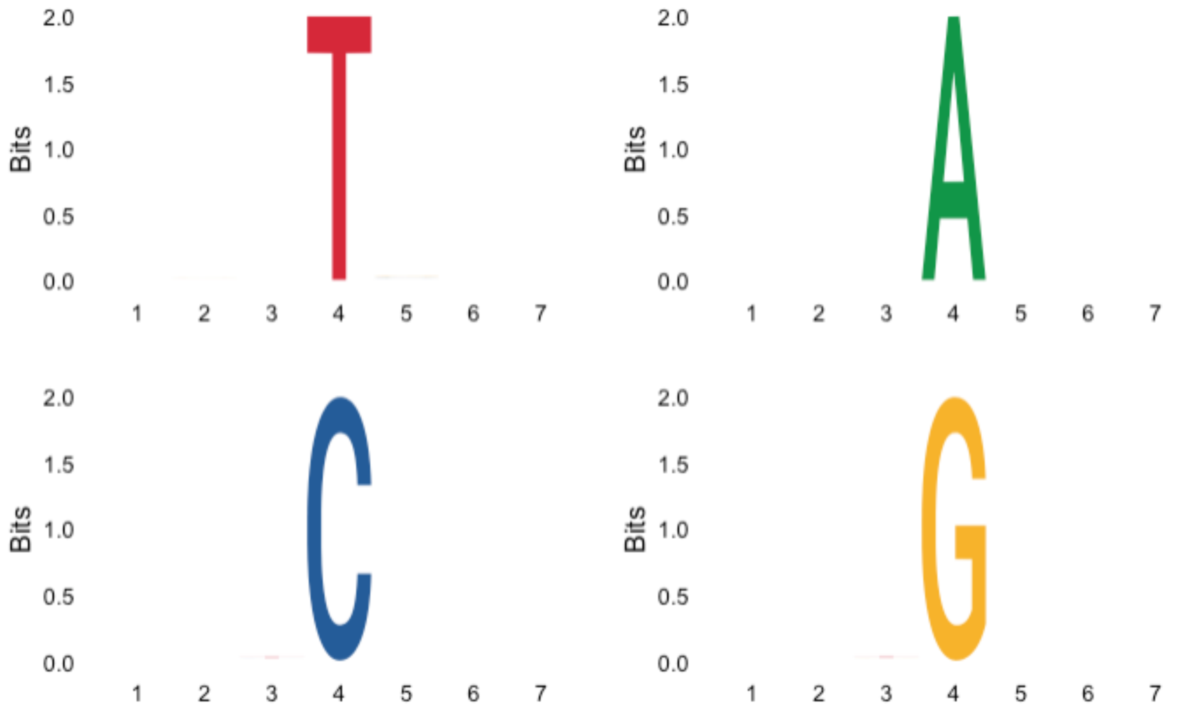

Fig. S30: Sequence context analysis of U vs 4SU positions in SLAM-seq reads. We counted 7-mers in a STAR-mapped SLAM-seq dataset (human HeLa wildtype cells treated with 5μm 4sU for 12h) that were centred at T (plus strand) or A (minus strand) reference bases, grouped the counts by the detected alternate base at this position and calculated sequence-logos with ggseqlogo [8]. For example, the lower-right 'G' sequence-logo was calculated from 7-kmers extracted from minus stand reads centred on a converted 4sU which resulted in an A-to-G mismatch in the readout. Overall, no significant sequence bias for 4sU positions was detected in this analysis which incorporated 2,242,645 reads and 35,922,779 kmers (~4% centred on a converted 4sU).

Supplementary references

[1] A. Liberzon, C. Birger, H. Thorvaldsdóttir, M. Ghandi, J. P. Mesirov and P. Tamayo, "The Molecular Signatures Database Hallmark Gene Set Collection," *Cell Systems,* vol. 1, no. 6, p. 417–425, December 2015.

[2] T. L. Bailey, J. Johnson, C. E. Grant and W. S. Noble, "The MEME Suite," *Nucleic Acids Research,* vol. 43, p. W39–W49, May 2015.

[3] L. Wang, S. Wang and W. Li, "RSeQC: quality control of RNA-seq experiments.," *Bioinformatics (Oxford, England),* vol. 28, no. 16, p. 2184–2185, August 2012.

[4] P. Danecek, J. K. Bonfield, J. Liddle, J. Marshall, V. Ohan, M. O. Pollard, A. Whitwham, T. Keane, S. A. McCarthy, R. M. Davies and H. Li, "Twelve years of SAMtools and BCFtools," *GigaScience,* vol. 10, no. 2, January 2021.

[5] C. Pockrandt, M. Alzamel, C. S. Iliopoulos and K. Reinert, "GenMap: ultra-fast computation of genome mappability," *Bioinformatics,* vol. 36, no. 12, p. 3687–3692, April 2020.

[6] Y. Liao, G. K. Smyth and W. Shi, "featureCounts: an efficient general purpose program for assigning sequence reads to genomic features," *Bioinformatics,* vol. 30, no. 7, p. 923–930, November 2013.

[7] T. Amort, D. Rieder, A. Wille, D. Khokhlova-Cubberley, C. Riml, L. Trixl, X.-Y. Jia, R. Micura and A. Lusser, "Distinct 5-methylcytosine profiles in poly(A) RNA from mouse embryonic stem cells and brain," *Genome Biology,* vol. 18, no. 1, January 2017.

[8] O. Wagih, "ggseqlogo: a versatile R package for drawing sequence logos," *Bioinformatics,* vol. 33, no. 22, pp. 3645-3647, 2017.
