# Supplementary material for: Splice_sim: a nucleotide conversion-enabled RNA-seq simulation and evaluation framework
Source: Genome Biol. 2024 Jun 25;25:166. doi: 10.1186/s13059-024-03313-8 (PMC11514792; doi:10.1186/s13059-024-03313-8)
Supplement: Supplementary file 2 — Additional file 2. Review History. [file 13059_2024_3313_MOESM2_ESM.docx]

**1^st^ round**

**Reviewer 1**

The authors present a simulation and evaluation workflow for the analyis of RNA-seq data that was subjected to nucleotide conversion, such as methylation.

As many such conversion protocols have been developed over time, this work addresses an interesting aspect. Given that short read sequencing has many biases, how are aligners and subsequent statistical estimates affected when the RNA sequence is modified? They present results for different modification protocols and alignment methods. They show that the aligners perform differently and that estimates are inaccurate in particular in regions of low mappability. The authors have created a well document software that is easy to install via Nextflow and containers and therefore useable to a large audience.

Major comments:

- In the introduction the authors mention that methods for bisulfite alignment use a three-nucleotide letter aligment strategy. This is not entirely correct, it has been shown that the alignment algorithms can be modified to directly solve the asymmetry of C/T to C alignment. It has been shown that this strategy improves on 3N alignments for bisulfite DNA data for bulk (LAST, Frith et al. NAR 2012, https://doi.org/10.1093/nar/gks275 OR Segemehl Otto et al. 2012 Bioinformatics https://doi.org/10.1093/bioinformatics/bts254 ) and single cell data (FAME, Fischer et al. Bioinformatics 2023 https://doi.org/10.1093/bioinformatics/btad386 ). It can be expected that the observed biases for RNA modifications investigated here, would also profit from RNA centered methods that correctly address the asymmetry. It would be great if the authors would discuss this, as this seems to be an open direction for future tool development for RNA aligners and modification estimation methods and could help to mitigate the biases detected by the authors.

- The study reveals that estimates of read conversion rates (FCR) are biased. As each mapper has its own biases, the authors suggest a mosaic approach, where gene regions that suffer from strong biases are removed. They also mention that the result from the two mappers may be combined to get the most reliable estimates. While this is a reasonable strategy to increase the accuracy of estimates based on their simulation, I wonder about the practicability of the idea? The authors mention that they report tables for which gene a particular mapper is problematic. How can one easily use these tables? Is there a downstream processing script as part of the software that one can use?

- Along those lines I am wondering whether the suggested removal of regions would generalize to other parameters of the sequencing experiments, such as read length? It would be great if the authors could address this.

Minor comments:

- Reference [19] appears to be incomplete

- page 7, lines 148,149 : the word strategies appears twice in the same sentence

-page 12, lines 266-271: the sentence is very long and hard to comprehend, shortening is advised

**Reviewer 2**

The paper evaluated mapping accuracies of the start-of-art spliced-read mappers for NC RNA-seq data. The design is over-simplified, not applicable to emerging techniques in the field. The impact will be limited.

1. There is no new method developed here. It is not appropriate to submit to METHOD.

2. Only site-specific conversion rate is considered. For chemical reaction with very high efficiency, like BS-seq, it is probably OK. However, enzyme based conversion efficiency rely on multiple factors, like local sequence motif. Other factors such as RNA secondary or tertiary structure will all impact the conversion. I don't think this process can be simulated by simply introducing some random conversion on the read level.

3. Essentially this paper is not evaluating NC RNA-seq techniques, but instead assessing the mapper's tolerance on mismatched reads.

4. What is the take-away conclusion? Any impact from the mismatches? Any impact on interpretation of NC RNA-seq results? Can we not use these tools? There are not clear at all.

**Authors’ response**

Reviewer 1

- In the introduction the authors mention that methods for bisulfite alignment use a three-nucleotide letter aligment strategy. This is not entirely correct, it has been shown that the alignment algorithms can be modified to directly solve the asymmetry of C/T to C alignment. It has been shown that this strategy improves on 3N alignments for bisulfite DNA data for bulk (LAST, Frith et al. NAR 2012 OR Segemehl Otto et al. 2012  and single cell data (FAME, Fischer et al. Bioinformatics 2023). It can be expected that the observed biases for RNA modifications investigated here, would also profit from RNA centered methods that correctly address the asymmetry. It would be great if the authors would discuss this, as this seems to be an open direction for future tool development for RNA aligners and modification estimation methods and could help to mitigate the biases detected by the authors.

We thank the Reviewer for these important remarks and agree that it would be interesting to include a hybrid mapping approach (combining a reduced-alphabet (3N) approach for seeding/kmer search with a bisulfite-aware alignment scoring scheme).

We have now extended *splice_sim* to additionally support Segemehl, a mapper that combines a 3N approach for its kmer (seed) search with a bisulfite-sensitive semi-global alignment. We have included Segemehl in our RNA-BS-seq analysis (Figure 4, pages 15ff), amended our introduction and have added a brief description of Segemehl’s hybrid mapping approach. We have also updated the main manuscript and the Supplement accordingly and have included Segemehl’s resource consumption in Figure S28.

- The study reveals that estimates of read conversion rates (FCR) are biased. As each mapper has its own biases, the authors suggest a mosaic approach, where gene regions that suffer from strong biases are removed. They also mention that the result from the two mappers may be combined to get the most reliable estimates. While this is a reasonable strategy to increase the accuracy of estimates based on their simulation, I wonder about the practicability of the idea? The authors mention that they report tables for which gene a particular mapper is problematic. How can one easily use these tables? Is there a downstream processing script as part of the software that one can use?

We thank the Reviewer for these important questions and agree that we have not explained how to apply our pre-calculated data tables to real-world data in sufficient detail.

We have now included an R Markdown (Rmd) script in *splice_sim* that demonstrates how to directly use the pre-calculated results for data filtering tasks and how to apply the described ‘mosaic’ approach to a user-defined dataset. A respective reference has been added to the Conclusions section of our Manuscript.

- Along those lines I am wondering whether the suggested removal of regions would generalize to other parameters of the sequencing experiments, such as read length? It would be great if the authors could address this.

We agree with the Reviewer that it would be interesting to apply *splice_sim* for an in-depth analysis of other sequencing parameters such as read length.

We have now simulated another metabolic labeling dataset ‘m_small_150’ with the same parameters as our ‘m_small’ dataset but with an increased read-length of SR150. When comparing these two datasets we found, as expected, that longer reads increase mappability along all categories but overall show the same characteristics as their shorter counterparts. We have now included a supplemental figure of this comparison (Figure S29) and added respective text to the main Manuscript and Supplement.

- Minor comments:
  - Reference [19] appears to be incomplete
  - page 7, lines 148,149 : the word strategies appears twice in the same sentence
  -page 12, lines 266-271: the sentence is very long and hard to comprehend, shortening is advised

We thank the Reviewer for spotting these errors. We have now updated reference [19] and revised the two mentioned sentences.

**Reviewer 2**

- There is no new method developed here. It is not appropriate to submit to METHOD.

Splice_sim distinguishes itself from previous RNA-seq simulators by its ability to simulate mixtures of regular and NC reads and its extensive evaluation module that provides users with detailed mapping accuracy assessments and additional resources (such as read highlighting and BAM files containing misaligned reads) that are useful for subsequent processing/analyses.

In the present paper we apply this new simulation and evaluation framework for a comprehensive analysis of read mapping accuracies and their impact on downstream data analysis tasks with direct biological interpretation that are under active investigation as demonstrated by the cited research.

We therefore believe that our manuscript qualifies as a method paper in Genome Biology.

- Only site-specific conversion rate is considered. For chemical reaction with very high efficiency, like BS-seq, it is probably OK. However, enzyme based conversion efficiency rely on multiple factors, like local sequence motif. Other factors such as RNA secondary or tertiary structure will all impact the conversion. I don't think this process can be simulated by simply introducing some random conversion on the read level.

We thank the reviewer for addressing this. Indeed, splice_sim simulates nucleotide conversions in reads based on Bernoulli processes with given conversion probabilities. We consider this a valid approach for the datasets we created:

- for BS-seq data with very high conversion rates (~98%), we agree with the reviewer opinion.
- For SLAM-seq data (with conversion rates of ~1-5%), no sequence motif biases were reported to the best of our knowledge. To confirm this, we conducted a kmer-analysis on a real SLAM-seq dataset and calculated sequence logos of kmers centred on U respectively (converted) 4sU positions (new Suppl. Fig S30). As expected, we could not detect any significantly enriched sequence motif.

We do, however, agree with the reviewer that a binomial distribution of NC would not be appropriate for all potential use-cases of *splice_sim*. In this regard we would like to point out that the NC simulation method of our open-source python implementation is encapsulated in a single method that can easily be customized for special use-cases (e.g., RNA-editing with a clear motif-bias). We have now added respective documentation to the README of the *splice_sim* implementation and respective text to the Discussion section of the main Manuscript.

- Essentially this paper is not evaluating NC RNA-seq techniques, but instead assessing the mapper's tolerance on mismatched reads. 
  What is the take-away conclusion? Any impact from the mismatches? Any impact on interpretation of NC RNA-seq results? Can we not use these tools? There are not clear at all.

Indeed, the goal of our paper was the comprehensive analysis of mapping accuracies of NC reads and our results should help with the interpretation of such data. Throughout the paper we demonstrate how minor differences in mapping accuracy between regular and nucleotide-converted reads that could easily be overlooked may lead to considerable numbers of outliers and false calls in downstream measures with direct biological interpretation, such as RNA stabilities or post-transcriptional methylation site calls as mentioned in the Discussion section and summarised in the Conclusions section.

We have now revised our Conclusion section to further emphasize the main take-aways from our study, including a list of potential use cases for *splice_sim. We have furthermore* added an R Markdown (Rmd) script to the *splice_sim* GitHub repository that demonstrates how to directly use the pre-calculated splice_sim results for data filtering and analysis tasks.

Regarding usability and availability of our software:

Splice_sim is publicly available and documented in detail at the provided GitHub link. We also provide a Docker container that contains all required 3rd party dependencies. Our pipelines are implemented as Nextflow workflows (<https://www.nextflow.io/>) which enable their executing in local as well as HPC environments. Our pre-calculated data tables containing mapping accuracies and additional meta-data are available in a publicly available Zenodo repository as described. These data tables can directly be used for data filtering tasks, and we have now added an R script to the splice_sim GitHub repository that demonstrates some common use-cases.

**2^nd^ round**

**Reviewer 1**

The authors have addressed my concerns and added a separate analysis script and extended their benchmark results. I think that the presented results are of general interest to the community.

**Reviewer 2**

It is a still overly-simplified model and a straightforward approach. For conversion in RNA, there are tons of factors, including expression level, allelic-specific expression, local nucleotide motif, accessibility of secondary structure and inhomogeneous enzymatic conversion rates. The author added some SLAM-seq data, but it is not very typical and representative. If their method is only improving RNA BS-seq, i don't think that is significant enough for Genome Biology. Even for BS-seq, there is no substantial conclusions on how much improvement will be gained.

*Based on these reports, we rejected the manuscript. The authors appealed*

**Authors’ response**

In your decision, you stated that “**Referee 2 still raises concerns about the simplicity of**

**the way errors are introduced for the RNA conversion data.**”, and reviewer #2 wrote

that “**It is a still overly-simplified model and a straightforward approach. For**

**conversion in RNA, there are tons of factors, including expression level, allelic-specific**

**expression, local nucleotide motif, accessibility of secondary structure and**

**inhomogeneous enzymatic conversion rates.**”

We believe that there is a **misunderstanding** of how nucleotide-conversion (NC) RNA

sequencing works. In current NC RNA-seq protocols (such as SLAM-seq, TimeLapse-seq,

TUC-seq, etc.), cells are incubated with a nucleotide analog (e.g., 4-thiouridine, 4sU),

eventually leading to a **mixture** of **‘regular’ nucleotides (U) and nucleotide analogs (4sU)**

**with a given ratio** in the nucleus. RNA polymerase then incorporates those into nascent

transcripts, **following** (to the best of our knowledge) **a Bernoulli random process** with a

‘success’ (i.e., 4sU incorporation) probability **determined by the present ratio between**

**4sU and U**. Eventually, after chemical modification and sequencing, 4sU is then read out

as cytosine, leading to T/C conversions in the readout. **This is exactly the simulation**

**approach we chose for the incorporation of nucleotide conversions in splice_sim** (see

Methods).

Incorporation of U or 4sU is **solely dependent on the 4sU/U ratio** and **no sequence motif**

**or other detectable biases** for the **incorporation of 4sU into nascent transcripts were**

**reported** in the literature. As reviewer #2 expressed these concerns already in the first

review round, we have additionally provided a **deep kmer analysis of a real SLAM-seq**

**dataset** and calculated sequence logos of kmers centred on U respectively (converted) 4sU

positions that showed **no enriched sequence motifs**. Also, in our **original SLAM-seq**

**paper** (Herzog et al., Nat Meth, 2017; https://www.nature.com/articles/nmeth.4435), **no**

**such biases were detected**.

For these reasons, we believe that the chosen simulation approach is **completely valid** for

the analysed data types (SLAM-seq as well as Bisulfite RNA sequencing (BS-RNA-seq) as

previously discussed in our revision correspondence) and is in no way an oversimplification.

We therefore speculate that reviewer #2’s concerns are **based on a different understanding**

**of how NC RNA-seq works**. Given his/her relatively vague statement (“For conversion in

RNA, there are tons of factors, including expression level, allelic-specific expression, local

nucleotide motif, accessibility of secondary structure and inhomogeneous enzymatic

conversion rates.”), however, we don’t know what NC methods he/she is referring to and

we would have liked the reviewer to provide some concrete examples in this regard.

We also want to make it totally clear that all of the above is **referring to protocol-induced**

**nucleotide conversions**, **not** sequencing errors.

**Reviewer 1**

I have read the authors appeal and I do second their opinion. Referee two's main concern is the "simplistic" simulation model. While from a pure biological and experimental standpoint I do agree that other factors such as inhomogenous conversion rates or allele-specific expression may additionally affect the conversion probabilities. However, from what the authors show I find that strong local nucleotide motifs of relevance are rather unlikely given. Therefore, in my point of view the argument by referee two is mainly to ask for a much more complex model, where it is unclear whether that model will make the simulation substantially more realistic, and more importantly, where it is unclear to how to train such a model.

I do agree that the approach of the authors is straightforward, but in my point of view I find this topic of simulation for these datasets novel and important for the community. In my experience often after a few years, when assays got established, scientists start to really look into the specific biases of experimental procedures and try to model them to come up with better analyses approaches. This work here is one of the first ones to address this generally for nucleotide conversion protocols on RNA level. Others will be inspired by this or use the simulators presented here and rethink some of the standards that are being used.
